# Diastereoselective synthesis of chiral 1,3-cyclohexadienals

**DOI:** 10.1371/journal.pone.0192113

**Published:** 2018-02-13

**Authors:** Aitor Urosa, Ignacio E. Tobal, Ángela P. de la Granja, M. Carmen Capitán, R. F. Moro, Isidro S. Marcos, Narciso M. Garrido, Francisca Sanz, Emilio Calle, David Díez

**Affiliations:** 1 Departamento de Química Orgánica, Facultad de Ciencias Químicas, Universidad de Salamanca, Salamanca, Spain; 2 Servicio de Difracción de Rayos X, Universidad de Salamanca, Salamanca, Spain; 3 Departamento de Química Física, Facultad de Ciencias Químicas, Universidad de Salamanca, Salamanca, Spain; Queen's University Belfast, UNITED KINGDOM

## Abstract

A novel approach to the production of chiral 1,3-cyclohexadienals has been developed. The organocatalysed asymmetric reaction of different β-disubstituted-α,β-unsaturated aldehydes with a chiral α,β-unsaturated aldehyde in the presence of a Jørgensen-Hayashi organocatalyst provides easy and stereocontrolled access to the cyclohexadienal backbone. This method allows for the synthesis of potential photoprotective chiral 1,3-cyclohexadienals and extra extended conjugation compounds in a simple manner.

## Introduction

**Fig 1:**
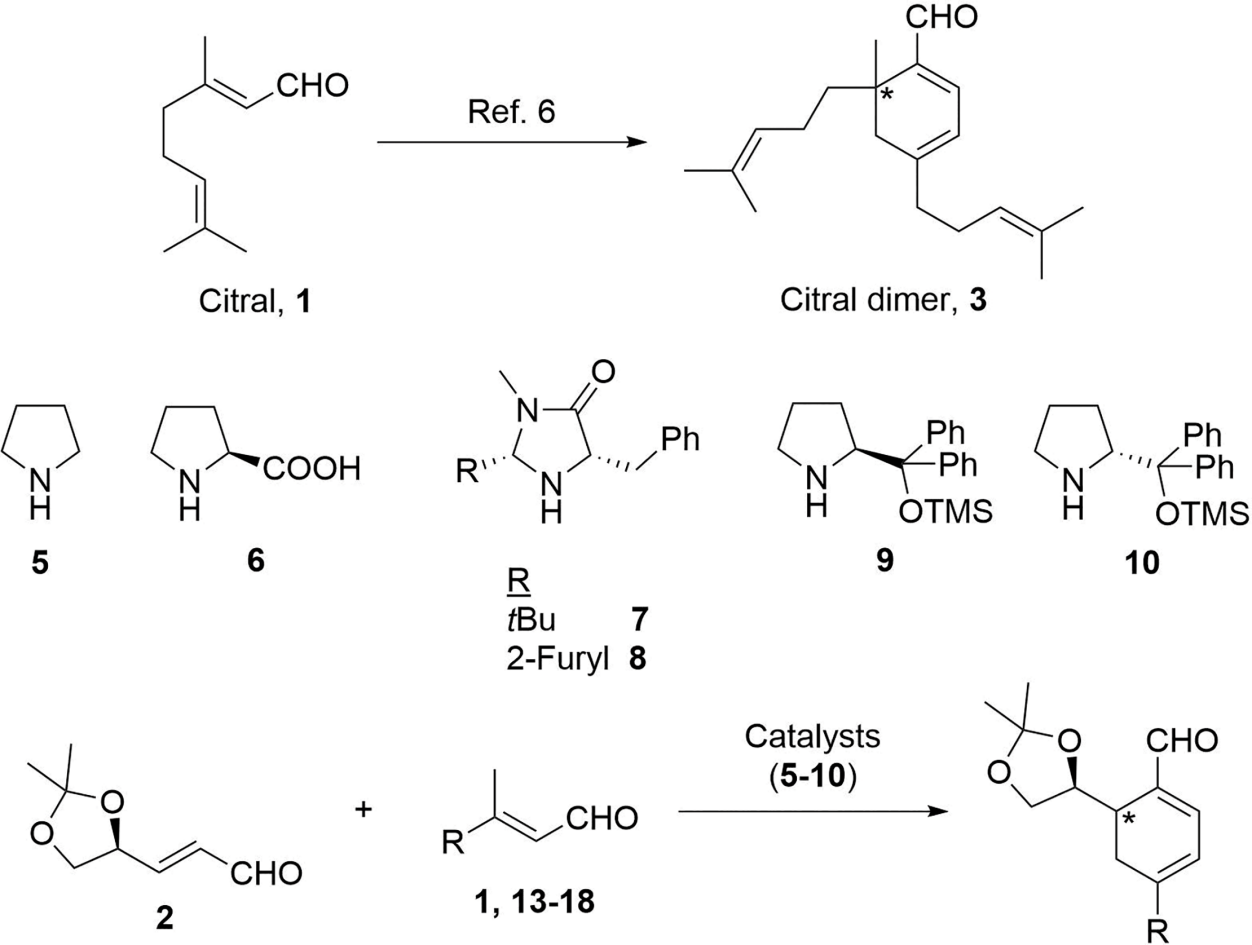
Proposal for the synthesis of new cyclohexadienal building blocks using different catalysts.

Organocatalysis is one of the fastest growing areas in organic chemistry [1–4]. The enantioselective organocatalytic Diels-Alder reaction from the seminal communication of Prof. MacMillan et al. [5] constitutes one of the most interesting research areas. The synthesis of enantiomerically enriched building blocks is an important task in organic synthesis, where cyclohexadienes [6–11] are of special interest due to their reactivity. Although the use of monosubstituted α,β-unsaturated aldehydes is more extended, in the last few years the use of β-disubstituted-α,β-unsaturated aldehydes has become more prevalent in this area. There are numerous examples of asymmetric synthesis by using organocatalysis, as shown by the work of Professor Serebryakov et al. in the synthesis of cyclohexa-1,3-dienes from prenal and unsaturated esters or derivatives, [12–16] Professor Hong et al. for the synthesis of aromatic aldehydes by organocatalytic [4+2] or [3+3] cycloaddition of α,β-unsaturated aldehydes [17–19] and Professor Watanabe et al. in citral, **1**, dimerization. [20–25] The cyclohexadienal scaffold has been shown to be bioactive in numerous cases throughout the literature. For example, the citral dimer shows antibiotic activity [26] and the retinal dimer could contribute to macular degeneration. [27] As chiral aldehyde **2** has been intensively used as a synthetic building block in the synthesis of bioactive natural products, [28–33] this study sought to obtain chiral cyclohexadienals using **2** in combination with different β-methyl disubstituted-α,β-unsaturated aldehydes in the presence of different catalysts (**5–10**), which avoid the dimerization of these compounds (Fig 1).

In the last few decades the potentially dangerous effects of UV radiation exposure have been extensively demonstrated [34–36]. While UVC light is filtered by the upper atmospheric layers, UVB and UVA light penetrate the upper layers of the atmosphere and reach the Earth’s surface. Photoprotection against this radiation can prevent skin damage and deleterious effects on DNA. However, it is important not to overdo protection against UVB as this can reduce the biosynthesis of vitamin D[37,38]. Therefore, photoprotective agents that selectively absorb UVB and UVA radiation are the UV-filters needed for developing effective and safe sunscreens.

**Fig 2:**
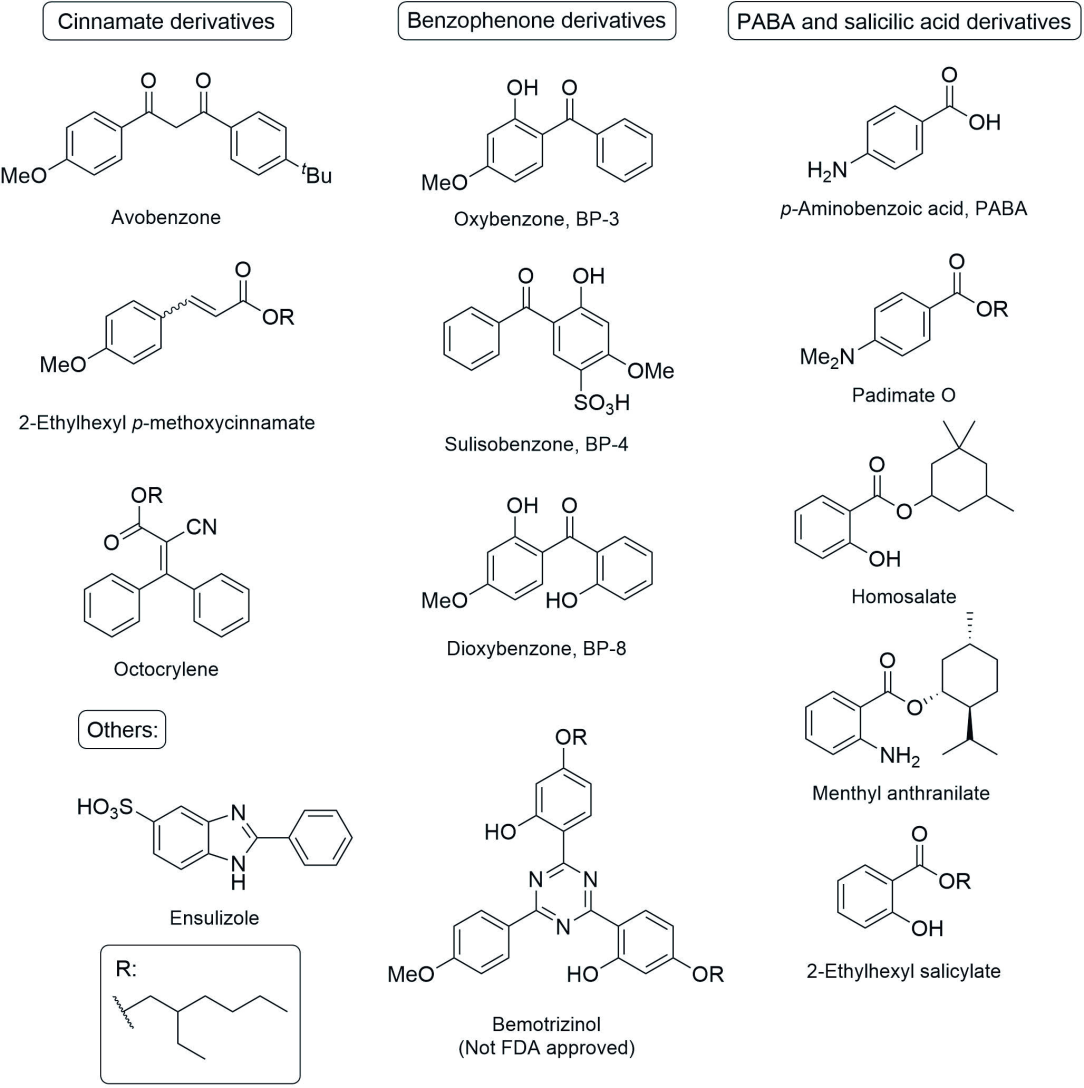
FDA approved UV-filters and bemotrizinol, approved only in Europe. The main UV filters structurally related to cinnamate esters, benzophenone, *p*-aminobenzoic acid (PABA) and salicilate derivatives, and two additional structures that can be found in ensulizole and bemotrizinol.

There are two groups of UV filters: inorganic and organic compounds. The inorganic filters scatter, reflect or absorb UV radiation, however, only TiO_2_ and ZnO are FDA approved. The organic UV filters consist of structurally simple aromatic molecules that absorb in UVA and UVB. The organic UV filters used in sunscreens, and approved by the FDA (Fig 2)[39] can be classified as cinnamates, benzophenones, PABA and salicilate derivatives and others. Despite their use in sunscreens, there are several studies regarding the toxicity, and especially the phototoxicity, of these compounds [40–46].

In this work, cyclohexadienals containing different substitutions have been synthesized as easily accessible high-conjugated compounds with interesting UV-Vis properties, making them suitable for use as photoprotective UV-filters.

## Materials and methods

All reactions were performed in oven-dried glassware under positive Ar pressure with magnetic stirring, unless otherwise noted. Air and moisture-sensitive liquids and solutions were transferred via a syringe or a stainless-steel cannula. TLC was performed on 0.25 mm E. Merck silica gel 60 F254 plates and visualized under UV light (λ = 254 nm) or by staining with potassium permanganate. Flash chromatography was performed on E. Merck 230–400 mesh silica gel 60. All reagents were purchased from commercial suppliers, and used without further purification unless otherwise noted. Solvents were distilled from suitable drying agents (CaH_2_ or Na wire) under an Ar atmosphere at 760 mmHg. All moisture- and/or oxygen-sensitive solids were handled and stored in a glove box under N_2_. The NMR spectra were recorded on Bruker AVANCE 400 MHz DRX and Varian Mercury 200 MHz using CDCl_3_ as solvent. NMR data is reported as follows: chemical shift (δ) (parts per million, ppm); multiplicity: s (singlet), d (doublet), t (triplet), q (quartet) and br (broad); coupling constants (J) are given in Hertz (Hz). ^1^H NMR chemical shifts were calibrated with respect to residual chloroform in CDCl_3_ centered at 7.26 ppm, whereas for ^13^C NMR, the center peak for CDCl_3_, centered at 77.0 ppm, was used for the calibration. The IR spectra were obtained on a Shimadzu IR Affinity-1 (film over NaCl). All NMR and IR spectra can be found in S1 File. The HRMS spectra were obtained on an Applied Biosystems QSTAR XL mass spectrometer. The optical rotation was performed on a Perkin-Elmer 241 digital polarimeter using cuvette with l = 1 dm and CHCl_3_ as the solvent. Absorbance measures were determined in 200–700 nm region using *i*PrOH as the solvent and an UV quartz cuvette (l = 1 cm) in a Shimadzu UV-2401PC spectrophotometer with thermostatic system at 20°C. The UV-Vis spectra can be found in S4 File.

## Results and discussion

**Fig 3:**
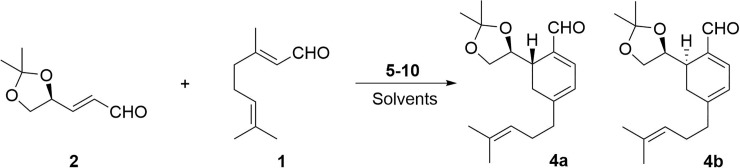
General reaction to obtain chiral cyclohexadienals.

First, the synthesis of chiral cyclohexadienals (Fig 3) with citral, **1**, and aldehyde, **2**, obtained from D-mannitol in the usual conditions was tested. [20–25].

The different experimental conditions of the catalyst, solvent and additives tested are shown in Table 1.

**Table 1:** Experimental optimization of synthesis of chiral cyclohexadienals (4a, 4b) from citral (1) and α,β-unsaturated aldehyde 2.

Entry	Cat.	Solvent^a^	Addit.^c^	Product^d^	d.r.^e^
1	**5**	CHCl_3_	**-**	**4a,b** (17)	50:50
2	**6**	**-**	**-**	**3** (8), **4a,b** (3)	n.d.
3	**6**	CHCl_3_	**-**	**3** (12), **4a,b** (10)	50:50
4	**7**	*i*PrOH	**-**	-	-
5	**8**	*i*PrOH	**-**	-	-
6	**9**	Hexane	**-**	**4a,b** (5)	80:20
7	**9**	Toluene	**-**	**4a,b** (20)	75:25
8	**9**	CHCl_3_^b^	**-**	**4a,b** (37)	85:15
9	**9**	DCM	**-**	**4a,b** (4)	n.d.
10	**9**	Et_2_O	**-**	**-**	-
11	**9**	THF	**-**	**-**	-
12	**9**	*i*PrOH	**-**	**-**	-
13	**9**	EtOH	**-**	**-**	-
14	**9**	MeOH	**-**	**-**	-
15	**9**	CHCl_3_	BzOH	**4a,b** (19)	60:40
16	**9**	CHCl_3_	*o*-NO_2_-BzOH	**4a,b** (27)	80:20
17	**9**	CHCl_3_	AcOH	-	-
18	**9**	CHCl_3_	TFA	-	-
19	**9**	CHCl_3_	TsOH	**4a,b** (2)	n.d.
20	**9**	CHCl_3_	(±) BINAP-OH	**4a,b** (4)	n.d.
21	**9**	CHCl_3_	DBU	**-**	-
22	**10**	CHCl_3_	**-**	**4a,b** (18)	33:66

^a^ All reactions were carried out with 0.5 equiv. of catalyst, solvent (0.2M), for 48 hours.

^b^ 20% and 30% of the catalyst produced lower yields in the same reaction time.

^c^ 0.2 equiv. of the additive were added and the reaction was carried out following the general procedure.

^d^In parentheses, the yield of the isolated mixture in %.

^e^The relation of the diastereoisomers was established by integrating ^1^H NMR in crude mixture.

When using a non-chiral organocatalyst, such as pyrrolidine, **5**, cyclohexadienal **4** was obtained in low yields, although without diastereoselectivity (entry 1). The use of L-proline, **6** (entries 2–3), using different solvents, or no solvent at all, gave the required cyclohexadienal **4** in very low yields and the citral dimer **3**, as a subproduct. Then, MacMillan´s organocatalysts **7** and **8** were tested, but no result was obtained (entries 4–5). In addition, the Jørgensen-Hayashi catalysts **9** and **10** were used in different solvent conditions, obtaining different results depending on the solvent used, ranging from moderate yields of cyclohexadienal **4** (entries 6–9 and 22) to no reaction at all (entries 10–14). As can be seen in Table 1, in some cases the reaction was carried out in presence of additives such as acids (BzOH, *o*-nitro-BzOH, AcOH, TsOH, (±)-1,19-binaphthyl-2,29-diyl hydrogenphosphate[(±) BINAP-OH] or TFA) and bases (DBU) (entries 15–21) with improved yields. The best result was obtained when the Jørgensen-Hayashi catalyst **9** in CHCl_3_ as the solvent, was used without any additional additive (entry 8) and produced a moderate yield and a good d.r.

### Determination of stereochemistry of stereocenter created by NMR

**Fig 4:**
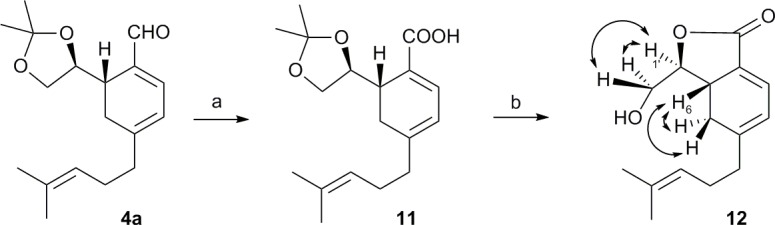
Synthesis of bicycle 12 from cyclohexadienal 4a. Reagents: a) NaH_2_PO_4_^.^H_2_O (2.2 equiv.), NaClO_2_ (5%, 2.2 equiv.), 2-methyl-2-butene, *t*BuOH, r.t., 2h, 99%; b) *p*-TsOH, MeOH, r.t., 30%.

An extra cycle was made to introduce more conformational rigidity (Fig 4), in order to establish the stereochemistry of cyclohexadienal **4**. Aldehyde **4a** was oxidized using the usual conditions [47] to obtain the acid **11**; deprotection of the acetonide gave the desired lactone ring, **12**. After studying the NOE (Nuclear Overhauser Effect) on this compound, the configuration of C-6 in compound **12** was established as *S*, because of NOE between H1’ and H6 did not appear. Later on, the absolute configuration was confirmed by X-Ray of an analogue (**24a**).

### Synthesis of chiral cyclohexadienals with different substituents

**Fig 5:**
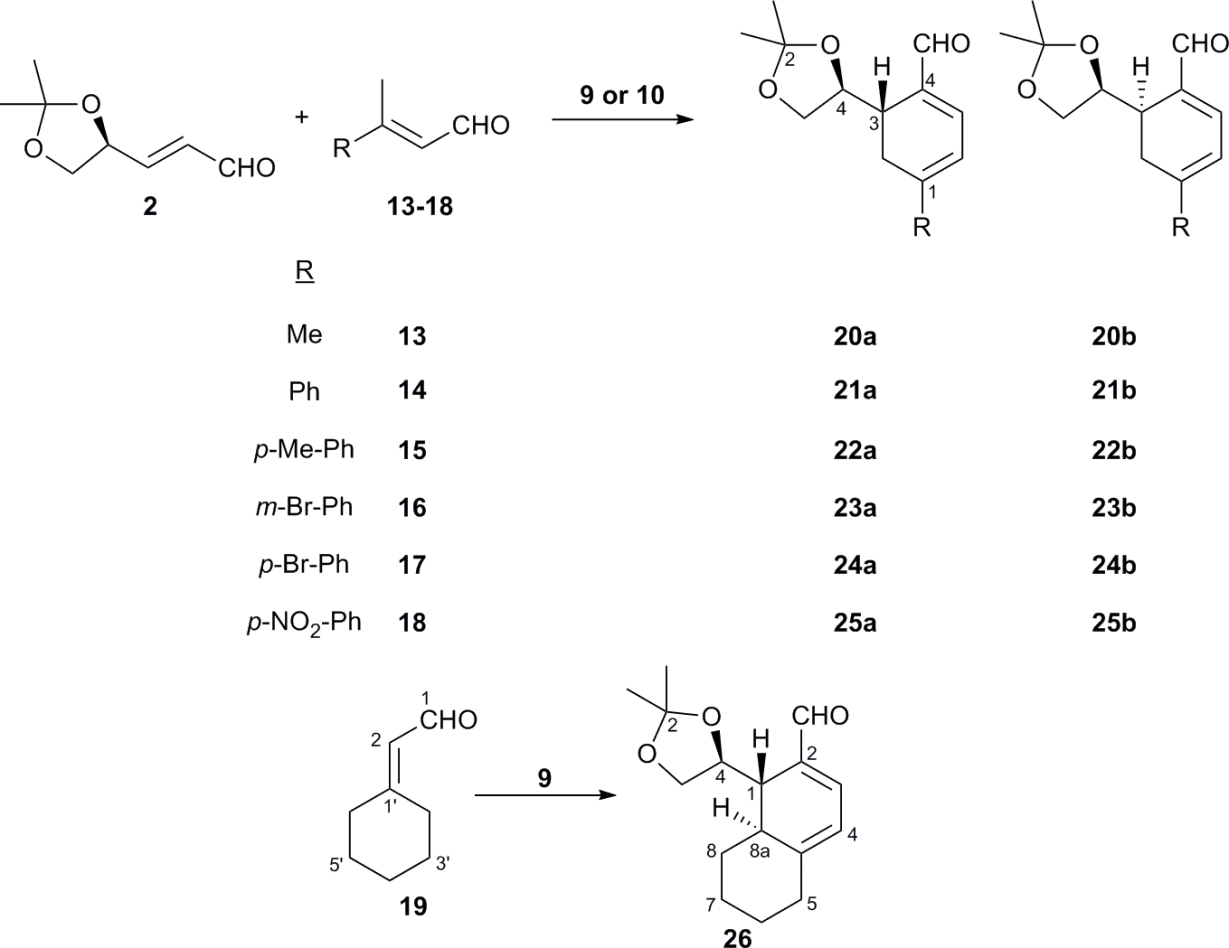
Synthesis of different chiral cyclohexadienals aromatic and non-aromatic compounds.

The mechanism could be understood by a Diels-Alder reaction, as suggested by Serebryakov et al. [12–16] and Watanabe et al. [20]. Similarly, this will would explain that the stereochemistry obtained in the final product does not depend on the Z or E stereochemistry of the α,β-unsaturation of the aldehyde used in the reaction. The same result was obtained with E-citral or a mixture E/Z-citral. E-citral was obtained from geraniol as described in the literature.[48] Once the conditions for the synthesis of cyclohexadienals were obtained, the generality of the reaction using different β-disubstituted-α,β-unsaturated aldehydes and **2** as starting materials was then observed, Fig 5 and Table 2.

**Table 2:** Synthesis of chiral cyclohexadienals (20a-26) from other β-disubstituted-α,β-unsaturated aldehydes (13–19)^a^.

Entry^b^	S.M.	Cat.	Product	Yield (%)^c^	d.r.^d^
1	**13**	**9**	**20a**	60	85:15
2	**13**	**10**	**20b**	52	>95
3	**14**	**9**	**21a**	72	90:10
4	**14**	**10**	**21b**	35	>95
5	**15**	**9**	**22a**	99	>95
6	**15**	**10**	**22b**	83	>95
7	**16**	**9**	**23a**	48	85:15
8	**16**	**10**	**23b**	50	>95
9	**17**	**9**	**24a**	45	90:10
10	**17**	**10**	**24b**	45	>95
11	**18**	**9**	**25a**	90	90:10
12	**18**	**10**	**25b**	88	>95
13	**19**	**9**	**26**	4	>95

^a^General procedure for the synthesis of **14–19** can be found in the S2 File.

^b^All reactions were carried out in CHCl_3_ (0.2M), 0.5 equiv. of catalyst, for 48 hours at r.t.

^c^ Isolated yield of major diastereomer.

^d^ Relation of the diastereoisomers was stablished by integrating ^1^H NMR in crude mixture.

The reaction was initiated using a simple α,β-unsaturated aldehyde such as **13**. When catalysts **9** or **10** were used, both produced a good yield and diastereoselection. When catalyst **10** was used, instead of **9**, the yield slightly decreased but diastereoselection remained complete. When using aromatic aldehydes, the reaction worked very well, especially with the *p-*methoxyphenyl group (entries 5–6) which produced excellent yields and diastereoselection with both catalysts **9** and **10**. When a bromophenyl group was used (entries 7–10), the yield and diastereoselection decreased but when a *p*-nitrophenyl group (entries 11–12) was used the yield increased with both catalysts and the diastereoselection was excellent, especially with catalyst **10**. When the reaction was run using an aliphatic cyclic aldehyde, such as catalyst **19**, the yield was very poor (entry 13) but diastereoselection was complete. As can be seen in Table 2, the reaction proceeded quite well, especially when using aromatic aldehydes.

### Crystallographic analysis of cyclohexadienal 24a

**Fig 6:**
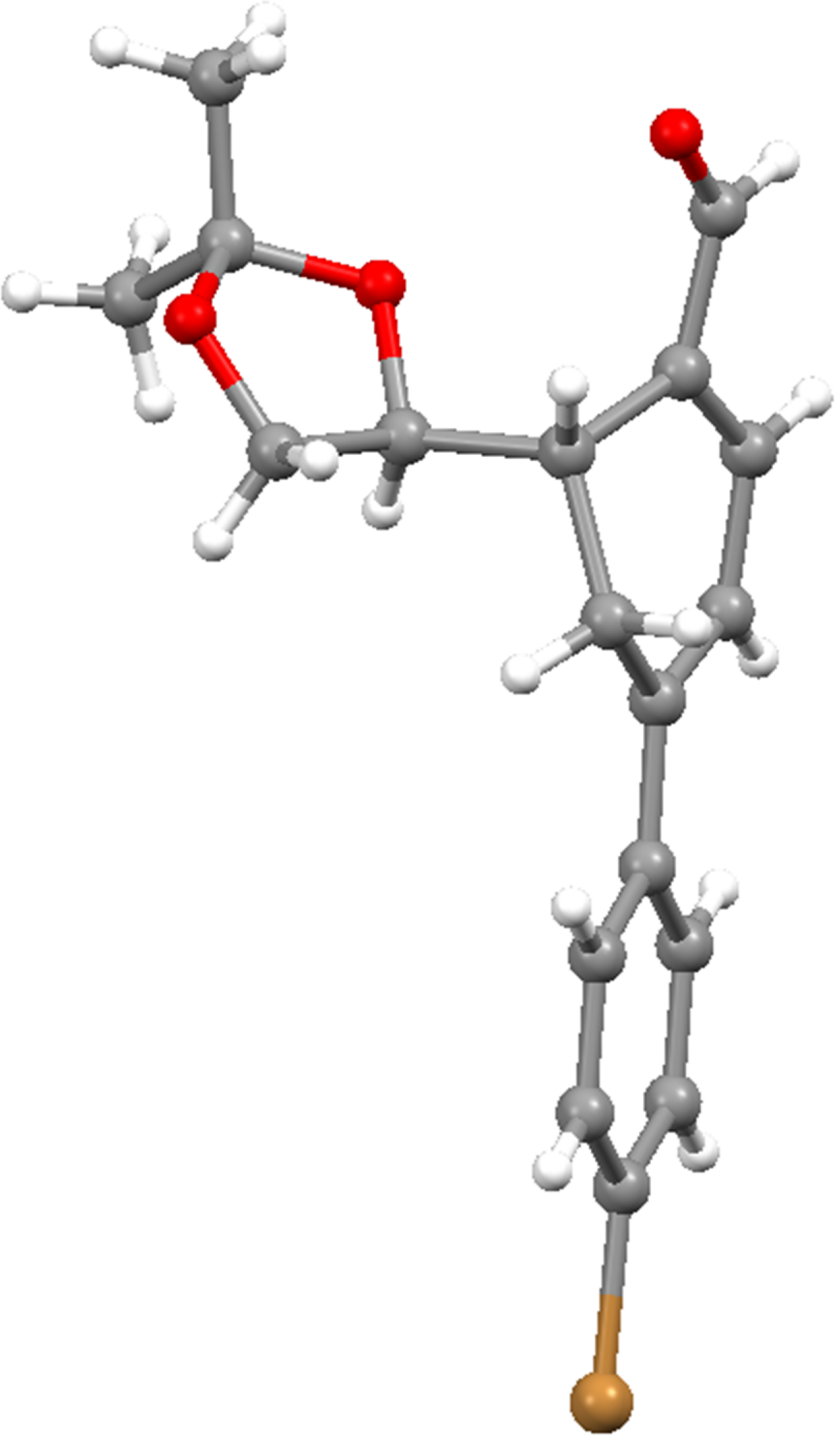
X-ray crystal structure of 24a. Displacement ellipsoids are drawn at the 30% probability level. Hydrogen atoms are shown as spheres of arbitrary radius (S3 File).

Compound **24a** was crystallized. In Fig 6, the X-ray crystal structure of compound **24a** [49] is shown and confirms the stereochemistry of compound **24a** at C-6. The stereochemistry of this compound was previously predicted by the NMR of compound **12**, and by analogy, the stereochemistry of compounds **20** to **26** was established.

### UV-Vis absorption analysis

The UV-Vis absorbance of different photostable cyclohexadienals was measured (Table 3 and S4 File) in order to test the possible application of these compounds as photoprotective agents.

**Table 3:** The area of regions UVA (315–400 nm) and UVB (280–315 nm) and molar extinction coefficient of some cyclohexadienals (4a, 20b, 21b, 22b, 23a, 23b) dissolved in *i*PrOH.

Entry	Product	Concentration (M/10^−6^)	λ_nm_ (ε M^-1^ cm^-1^)	AUC (UVA)^a^	λ_nm_ (ε M^-1^ cm^-1^)	AUC (UVB)
1	**4a**	1.8	-	0.305	-	0.318
2	**20b**	5.3	341.3 (3000)	1.034	-	0.510
3	**21b**	8.5	351.5 (13200)	7.013	283.0 (2000)	2.370
4	**22b**	4.2	360.3 (4300)	1.194	274.8 (8900)	0.594
5	**23a**	7.4	336.3 (8000)	3.708	282.3 (10900)	2.310
6	**23b**	1.7	341.3 (2000)	2.374	287.8 (34700)	1.838

^a^ Area Under Curve (AUC).

The majority of the compounds at concentrations in the order of 10^−6^ absorbed UVA and UVB. Compound **21b** exhibited values suitable for photoprotection against UVA owing to the higher area under the curve (AUC) at that particular wavelength region and its molar extinction coefficient (ε = 13200 M^-1^cm^-1^). The best results found in the UVB region were shown by compound **23b** which had an extinction coefficient of 34700 M^-1^cm^-1^ at 288nm. However, the compound that was able to better absorb UVA and UVB was **23a**, with molar extinction coefficients of 8000 M^-1^cm^-1^ in UVA and 10900 M^-1^cm^-1^ in UVB.

**Fig 7:**
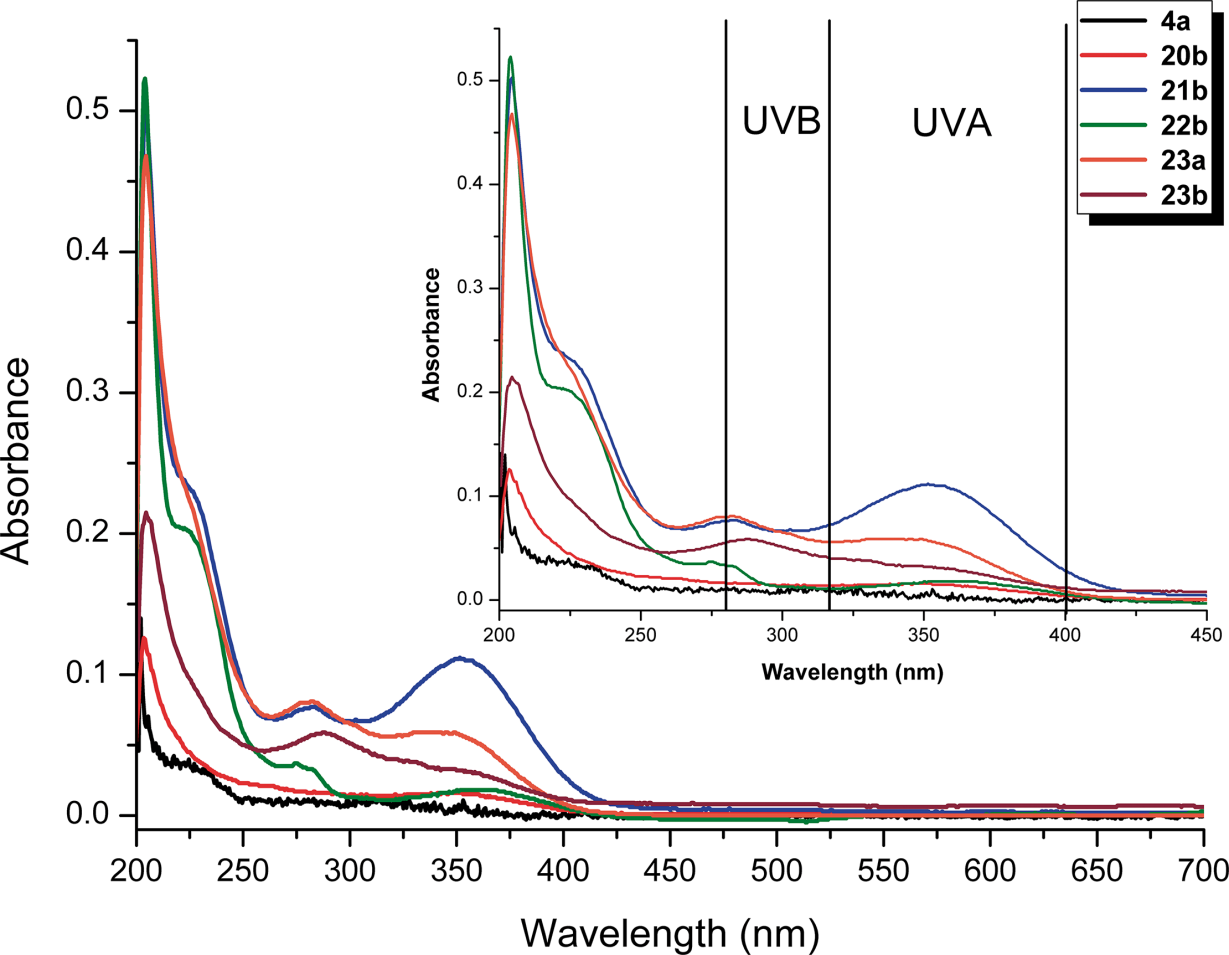
UV-Vis absorbance spectra at different λ of 4a, 20b, 21b, 22b, 23a, 23b. Amplification of the 200–450 nm region and the delimited UVA and UVB regions (ISO-21348).

A global view of UV absorption of this chiral aromatic cyclohexadienal can be seen in Fig 7.

### Synthesis

#### General procedure for the optimization of conditions for cyclohexadienals (4a,b)

Catalyst **5**–**10** (0.5 eq) were added to a solution containing **2** (0.3 mmol, 1 equiv.) and **1** (0.3 mmol, 1 equiv.) in solvent (1.5 mL, 0.2M) at r.t. The reaction mixture was stirred at r.t. for 48h. The solution was concentrated in and the residue was purified by flash column chromatography (EtAcO:hexane) to obtain cyclohexadienals **4a** and **4b** as a yellow oil and dimer **3** as a colourless oil.

Catalyst **9** (0.5 eq) was added to a solution containing **2** (0.15 mmol, 1 equiv.) and E-citral (0.15 mmol, 1 equiv.) in CHCl_3_ (0.75 mL, 0.2M) at r.t. The reaction mixture was stirred at r.t. for 48h. The solution was concentrated in vacuum and the residue was purified by flash column chromatography (EtAcO:hexane) to obtain a mixture of cyclohexadienals **4a** and **4b** as a yellow oil (yield 37%; d.r. 85:15).

6-Methyl-4,6-bis(4-methylpent-3-en-1-yl)cyclohexa-1,3-diencarbaldehyde(3).

^1^H NMR (200 MHz, CDCl_3_): δ = 9.41 (1H, s), 6.67 (1H, d, J = 5.5 Hz), 5.92 (1H, d, J = 5.5 Hz), 5.10–5.03 (2H, m), 2.38–2.33 (1H, m), 2.19–2.18 (4H, m), 2.04–1.77 (4H, m, H-5), 1.69 (3H, s), 1.65 (3H, s), 1.62 (3H, s), 1.55 (3H, s), 1.41–1.32 (1H, m), 1.19 (3H, s).

(*S*)-6-((*S*)-2,2-Dimethyl-1,3-dioxolan-4-yl)-4-(4-methylpent-3-en-1-yl)cyclohexa-1,3- dien-1-carbaldehyde(4a).

[α]_D_^25^ = -43.3 (c = 0.54, CHCl_3_).

IR (film): 2981, 2929, 1670, 1570, 1379, 1213, 1066, 842 cm^-1^.

^1^H NMR (200 MHz, CDCl_3_): δ = 9.47 (1H, s), 6.81 (1H, d, J = 5.7 Hz), 5.95–5.90 (1H, m), 5.10–5.00 (1H, m), 4.18 (1H, q, J = 6.4 Hz), 3.86 (1H, dd, J = 8.4, 6.4 Hz), 3.67 (1H, dd, J = 8.4, 6.4 Hz), 3.20–3.10 (1H, m), 2.44–2.37 (2H, m), 2.36–1.80 (4H, m), 1.68 (3H, s), 1.61 (3H, s), 1.42 (3H, s), 1.27 (3H, s).

^13^C NMR (50 MHz, CDCl_3_): δ = 192.4, 151.8, 145.2, 135.1, 132.8, 123.2, 118.7, 109.0, 75.9, 66.7, 38.0, 31.7, 28.8, 26.4, 25.9 (2), 25.5, 18.0.

HRMS (ESI): Calculated for C_18_H_26_O_3_Na ([M+Na]^+^): 313.1774; found 313.1775.

(*R*)-6-((*S*)-2,2-Dimethyl-1,3-dioxolan-4-yl)-4-(4-methylpent-3-en-1-yl)cyclohexa-1,3-dien-1-carbaldehyde(4b).

[α]_D_^25^ = 33.5 (c = 0.45, CHCl_3_).

IR (film): 2981, 2929, 1670, 1570, 1379, 1213, 1066, 842cm^-1^.

^1^H NMR (200 MHz, CDCl_3_): δ = 9.46 (1H, s), 6.84 (1H, d, J = 5.7 Hz), 6.00–5.97 (1H, m), 5.13–5.08 (1H, m), 3.92 (1H, q, J = 6.7 Hz), 3.75 (1H, dd, J = 15.6, 8.0 Hz), 3.72 (1H, dd, J = 15.6, 6.7 Hz), 2.94 (1H, t, J = 8.5 Hz), 2.66 (1H, d, J = 18.0), 2.37 (1H, dd, J = 18.0, 8.5 Hz), 2.33–2.10 (4H, m), 1.69 (3H, s), 1.62 (3H, s), 1.54 (3H, s), 1.30 (3H, s).

^13^C NMR (50 MHz, CDCl_3_): δ = 192.3, 152.6, 146.5, 134.9, 132.4, 123.1, 118.6, 108.6, 74.8, 68.0, 37.9, 32.2, 29.6, 26.8, 25.8, 25.7, 25.6, 17.7.

HRMS (ESI): Calculated for C_18_H_26_O_3_Na ([M+Na]^+^): 313.1774; found 313.1775.

6-((*S*)-2,2-dimethyl-1,3-dioxolan-4-yl)-4-(4-methylpent-3-en-1-yl)cyclohexa-1,3-dien-1-carboxilic acid(11).

2-methyl-2-butene (0.097 mL, 0.92 mmol), a 0.65M solution of NaH_2_PO_4_^.^H_2_O in H_2_O (0.97 mL, 0.81 mmol) and 5% NaClO_2_ in H_2_O (0.91 mL, 0.72 mmol) were added to a solution containing **4a** (105 mg, 0.36 mmol) in *t*BuOH (3.8 mL). The reaction mixture was stirred at r.t. for 22h. The reaction was quenched with H_2_O and 1M HCl was added until acid pH was reached. The reaction mixture was extracted with EtOAc (3x10 mL). The combined organic layers were washed with H_2_O until neutral pH was reached, dried over Na_2_SO_4_, filtered and concentrated under vacuum to obtain acid **11** (109 mg, 0.36 mmol, 99%).

[α]_D_^25^ = -63.0, (c = 0.684, CHCl_3_).

IR (film): 2984, 2930, 1678, 1582, 1422, 1260, 1217, 1070, 1049 cm^-1^.

^1^H NMR (200 MHz, CDCl_3_): δ = 7.20 (1H, d, J = 5.8 Hz), 5.82 (1H, d, J = 5.8 Hz), 5.07 (1H, bs), 4.27 (1H, q, J = 6,2 Hz), 3.92 (1H, dd, J = 8.4, 6.2 Hz), 3.72 (1H, dd, J = 8.4, 7.4 Hz), 3.06 (1H, t, J = 8.0 Hz), 2.45 (1H, d, J = 8.0 Hz), 2.37 (1H, bs), 2.17 (4H, bs), 1.68 (3H, s), 1.61 (3H, s), 1.41 (3H, s), 1.31 (3H, s).

^13^C NMR (50 MHz, CDCl_3_): δ = 172.6, 148.8, 137.6, 132.7, 132.7, 124.1, 118.3, 109.0, 76.4, 66.9, 37.7, 33.6, 28.8, 26.4, 25.9 (2), 25.5, 18.0.

HRMS (ESI): Calculated for C_18_H_27_O_4_ ([M+H]^+^): 307.1904; found 307.1908.

(3*S*,3a*R*)-3-(Hydroxymethyl)-5-(4-methylpent-3-en-yl)-3a,4-dihydroisobenzofuran-1(3H)-one(12).

*p*-TsOH (21 mg, 0.11 mmol) was added to a solution containing **11** (35mg, 0.11 mmol) and MeOH (1.5 mL). The reaction mixture was stirred at r.t. for 14h. The reaction was quenched with H_2_O. The crude mixture was extracted with EtOAc (3x10 mL). The combined organic layers were washed with H_2_O, sat. NaHCO_3_ solution and brine, dried over Na_2_SO_4_, filtered and concentrated under vacuum to yield **12** (8 mg, 0.033 mmol, 30%).

IR (film): 2959, 2924, 1749, 1217, 1030 cm^-1^.

^1^H NMR (400 MHz, CDCl_3_): δ = 6.94 (1H, dd, J = 5.4, 3.3 Hz), 6.02(1H, bs), 5.07 (1H, bs), 4.24 (1H, dt, J = 8.2, 3.9 Hz), 3.98 (1H, d, J = 12.6 Hz), 3.76 (1H, d, J = 12.6 Hz), 2.99 (1H, dtd, J = 17.6, 8.2, 3.9 Hz), 2.36 (2H, dd, J = 17.6, 8.2 Hz), 2.28–2.14 (5H, m), 1.69 (3H, s), 1,61 (3H, s).

^13^C NMR (50 MHz, CDCl_3_): δ = 169.3, 147.6, 132.9, 131.1, 124.3, 123.2, 120.3, 85.9, 63.0, 37.8, 35.0, 31.7, 26.3, 25.9, 18.0.

HRMS (ESI): Calculated for C_15_H_21_O_3_ ([M+H]^+^): 249.1485; found 249.1491.

#### General procedure for the synthesis of cyclohexadienals (20a,b-26)

Catalyst **9** or **10** (0.5 equiv.) was added to a solution of **2** (1 mmol, 1 equiv.) and aldehyde (1 mmol) in CHCl_3_ (5 mL) at r.t. The reaction mixture was stirred at r.t. for 48h. The solution was concentrated under vacuum and the residue was purified by flash column chromatography (EtOAc:hexane) to obtain cyclohexadienal as a yellow oil.

(*S*)-3-((*S*)-2,2-Dimethyl-1,3-dioxolan-4-yl)-1-methylcyclohexa-4,6-dien-4-carbaldehyde(20a).

Catalyst **9** used.

Yield: 60% (133 mg, 0.60 mmol).

[α]_D_^25^ = -114.5 (c = 0.53, CHCl_3_).

IR (film): 2916, 2848, 1672, 1059 cm^-1^.

^1^H NMR (200 MHz, CDCl_3_): δ = 9.48 (1H, s), 6.82 (1H, d, J = 5.6 Hz), 5.92 (1H, d, J = 5.6 Hz), 4.21 (1H, q, J = 6.4 Hz), 3.87 (1H, dd, J = 8.4, 6.4 Hz), 3.68 (1H, dd, J = 8.4, 6.4 Hz), 3.17 (1H, ddd, J = 8.4, 6.4, 3.4 Hz), 2.41 (2H, d, J = 3.4 Hz), 1.91 (3H, s), 1.43 (3H, s), 1.30 (3H, s).

^13^C NMR (50 MHz, CDCl3): δ = 192.4, 148.6, 145.5, 134.8, 119.3, 109.0, 76.1, 66.6, 31.7, 30.0, 26.4, 25.5, 24.2.

HRMS (ESI): Calculated for C_13_H_18_O_3_Na ([M+Na]^+^): 245.1148; found 245.1146.

(*R*)-3-((*S*)-2,2-Dimethyl-1,3-dioxolan-4-yl)-1-methylcyclohexa-4,6-dien-4-carbaldehyde(20b).

Catalyst **10** used.

Yield: 52% (116 mg, 0.52 mmol).

[α]_D_^25^ = +12.7 (c = 1.65, CHCl_3_).

IR (film): 2985, 2933,1666, 1573, 1192, 1155, 1066, 860 cm^-1^

^1^H NMR (200 MHz, CDCl_3_): δ = 9.46 (1H, s), 6.83 (1H, d, J = 5.5 Hz), 6.01–5.95 (1H, m), 4.03–3.88 (1H, m), 3.85–3.65 (2H, m), 2.94 (1H, dt, J = 8.4, 1.7 Hz), 2.61 (1H, dd, J = 18.4, 1.7 Hz), 2.38 (1H, dd, J = 18.4, 8.4 Hz), 1.95 (3H, s), 1.39 (3H, s), 1.31 (3H, s).

^13^C NMR (50 MHz, CDCl_3_): δ = 192.6, 149.3, 147.0, 136.5, 119.4, 108.9, 75.5, 68.0, 32.5, 31.0, 27.0, 25.9, 24.3.

HRMS (ESI): Calculated for C_13_H_19_O_3_Na ([M+Na]^+^): 223.1328; found 223.1326.

(*S*)-3-((*S*)-2,2-Dimethyl-1,3-dioxolan-4-yl)-2,3-dihydro-[1,1’-biphenyl]-4-carbaldehyde(21a).

Catalyst **9** used.

Yield: 72% (205 mg, 0.72 mmol).

[α]_D_^25^ = -34.1 (c = 0.16, CHCl_3_).

IR (film): 2983, 2931, 1668, 1554, 1172, 756 cm^-1^.

^1^H NMR (200 MHz, CDCl_3_): δ = 9.60 (1H, s), 7.53 (2H, dd, J = 8.0, 1.6 Hz), 7.39 (2H, d, J = 8.0 Hz), 7.41–7.37 (1H, m), 7.01 (1H, d, J = 6.0 Hz), 6.57 (1H, d, J = 6.0 Hz), 4.30 (1H, q, J = 6.4 Hz), 3.90 (1H, dd, J = 8.4, 6.4 Hz), 3.76 (1H, dd, J = 8.4, 6.4 Hz), 3.36–3.27 (1H, m), 2.95–2.86 (2H, m), 1.29 (3H, s), 1,40 (3H, s).

^13^C NMR (50 MHz, CDCl_3_): δ = 192.3, 146.4, 144.4, 139.3, 136.2, 129.4, 129.0 (2), 126.0 (2), 119.6, 109.2, 76.0, 66.8, 32.0, 27.3, 26.5, 25.6.

HRMS (ESI): Calculated for C_18_H_20_O_3_Na ([M+Na]^+^): 307.1305; found 307.1300.

(*R*)-3-((*S*)-2,2-Dimethyl-1,3-dioxolan-4-yl)-2,3-dihydro-[1,1’-biphenyl]-4-carbaldehyde(21b).

Catalyst **10** used.

Yield: 35% (100 mg, 0.35 mmol).

[α]_D_^25^ = -27.3 (c = 0.07, CHCl3).

IR (film): 2985, 2933, 1666, 1548, 1170, 756 cm^-1^.

^1^H NMR (200 MHz, CDCl_3_): δ = 9.56 (1H, s), 7.38 (2H, dd, J = 8.0 Hz, 1.6 Hz), 7.37–7.35 (1H, m), 7.20 (1H, d, J = 9.0 Hz), 7.19 (2H, dd, J = 8.0 Hz, 1.6 Hz), 6.59 (1H, dd, J = 9.0 Hz, 2.2 Hz), 4,07–3.97 (1H, m), 3.86–3.74 (2H, m), 3.25 (1H, d, J = 17.9 Hz), 3.05 (1H, dt, J = 8.4, 1.5 Hz), 2.68 (1H, ddd, J = 17.9, 8.4, 2.9 Hz, H-6), 1.36 (3H, s), 1.28 (3H, s).

^13^C NMR (50 MHz, CDCl_3_): δ = 197.3, 192.5, 146.2, 139.7, 136.1, 129.3,128.9 (2), 126.4 (2),119.8, 109.0,75.2, 68.1, 32.8, 27.1, 25.9, 21.4.

HRMS (ESI): Calculated for C_18_H_21_O_3_Na ([M+Na]^+^):285.1461; found 285.1485.

(*S*)-3-((*S*)-2,2-Dimethyl-1,3-dioxolan-4-yl)-4’-methyl-2,3-dihydro-[1,1’-biphenyl]-4-carbaldehyde(22a).

Catalyst **9** used.

Yield: 99% (295 mg, 0.99 mmol).

[α]_D_^25^ = - 51.2 (c = 0.16, CHCl_3_).

IR (film): 3030, 2985, 2873, 2720, 1675, 1170, 1061, 858, 810 cm^-1^

^1^H NMR (200 MHz, CDCl_3_): δ = 9.58 (1H, s), 7.44 (2H, d, J = 8.0 Hz), 7.21 (2H, d, J = 8.0 Hz), 6.99 (1H, d, J = 5.8 Hz), 6.53 (1H, dd, J = 5.8, 2.5 Hz), 4.28 (1H, q, J = 6.3 Hz), 3.89 (1H, dd, J = 8.2, 6.3 Hz), 3.73 (1H, dd, J = 8.2, 6.3 Hz), 3.31 (1H, ddd, J = 9.2, 6.0, 2.3 Hz), 3.11–2.68 (2H, m), 2.38 (3H, s), 1.40 (2H, s), 1.29 (3H, s).

^13^C NMR (50 MHz, CDCl3): δ = 192.0, 146.2, 144.4, 139.4, 136.1, 135.7, 129.5 (2), 125.7 (2), 118.5, 108.9, 75.8, 66.6, 31.8, 27.0, 26.3, 25.4, 21.3.

HRMS (ESI): Calculated for C_19_H_23_O_3_ ([M+H]^+^): 299.1642; found 299.1645.

(*R*)-3-((*S*)-2,2-Dimethyl-1,3-dioxolan-4-yl)-4’-methyl-2,3-dihydro-[1,1’-biphenyl]-4-carbaldehyde(22b).

Catalyst **10** used.

Yield: 83% (248 mg, 0.83 mmol).

[α]_D_^25^ = +23.1 (c = 0.08, CHCl_3_).

IR (film): 3030, 2984, 2873, 2717, 1668, 1170, 1067, 854, 810 cm^-1^.

^1^H NMR (200 MHz, CDCl_3_): δ = 9.55 (1H, s), 7.48 (2H, d, J = 8.0 Hz), 7.21 (2H, d, J = 8.0 Hz), 7.01 (1H, d, J = 5.9 Hz), 6.55 (1H, dd, J = 5.9, 2.8 Hz), 4.02 (1H, q, J = 7.5 Hz), 3.90–3.68 (2H, m), 3.28 (1H, d, J = 17.9 Hz), 3.10 (1H, dt, J = 8.2, 1.4 Hz), 2.71 (1H, ddd, J = 17.9, 8.2, 2.8 Hz), 2.38 (3H, s), 1.37 (3H, s), 1.27 (3H, s).

^13^C NMR (50 MHz, CDCl_3_): δ = 192.2, 147.2, 146.2, 139.3, 136.6, 135.6, 129.4 (2), 126.1 (2), 118.8, 108.8, 75.1, 67.9, 32.7, 28.1, 26.8, 25.7, 21.3.

HRMS (ESI): Calculated for C_19_H_23_O_3_ ([M+H]^+^): 299.1642; found 299.1642.

(*S*)-3’-bromo-3-((*S*)-2,2-Dimethyl-1,3-dioxolan-4-yl)-2,3-dihydro-[1,1’-biphenyl]-4-carbaldehyde(23a).

Catalyst **9** used.

Yield: 48% (174mg, 0.48 mmol).

[α]_D_^25^ = -35.4 (c = 0.38, CHCl_3_).

IR (film): 2984, 2876, 2814, 2718, 1670, 1551, 1211, 1173, 1069, 847, 781 cm^-1^.

^1^H NMR (200 MHz, CDCl_3_): δ = 9.55 (1H, s), 7.61 (1H, t, J = 1.9 Hz), 7.48–7.34 (2H, m), 7.20 (1H, d, J = 7.9 Hz), 6.95 (1H, d, J = 5.9 Hz), 6.50 (1H, dd, J = 5.9, 2.4 Hz), 4.23 (1H, q, J = 6.3 Hz), 3.86 (1H, dd, J = 8.4, 6.3 Hz), 3.68 (1H, dd, J = 8.4, 6.3 Hz), 3.25 (1H, ddd, J = 9.1, 6.0, 3.1 Hz), 2.95–2.65 (2H, m), 1.36 (3H, s), 1.24 (3H, s).

^13^C NMR (50 MHz, CDCl_3_): δ = 192.2, 144.5, 143.7, 141.5, 136.7, 132.0, 130.5, 129.0, 124.5, 123.2, 120.6, 109.1, 78.1, 77.5, 76.8, 76.0, 66.7, 31.9, 27.3, 26.5, 25.5.

HRMS (ESI): Calculated for C_18_H_19_O_3_NaBr ([M+Na]^+^): 385.0410 and 387.0389; found 385.0405 and 387.0384.

(*R*)-3’-bromo-3-((*S*)-2,2-Dimethyl-1,3-dioxolan-4-yl)-2,3-dihydro-[1,1’-biphenyl]-4-carbaldehyde(23b).

Catalyst **10** used.

Yield: 50% (192 mg, 0.50mmol).

[α]_D_^25^ = -5.7 (c = 0.07, CHCl_3_).

IR (film): 2984, 2934, 2878, 2815, 1670, 1549, 1169, 1067, 847, 782, 515 cm^-1^.

^1^H NMR (400 MHz, CDCl_3_): δ = 9.56 (1H,s), 7.67 (1H, t, J = 1.9 Hz), 7.46 (1H, dd, J = 8.2, 1.9 Hz), 7.25 (1H, t, J = 8.2 Hz), 7.00 (1H, d, J = 5.8 Hz), 6.55 (1H, dd, J = 5.8, 2.9 Hz), 3.98 (1H, dt, J = 8.4, 6.4 Hz), 3.89–3.70 (2H, m), 3.19 (1H, dd, J = 17.9, 1.5 Hz), 3.08 (1H, dt, J = 8.4, 1.5 Hz), 2.70 (1H, ddd, J = 17.9, 8.4, 2.9 Hz), 1.36 (3H, s), 1.27 (3H, s).

^13^C NMR (100 MHz, CDCl_3_): δ = 192.2, 145.3, 145.1, 141.6, 136.5, 131.7, 130.1, 129.1, 124.7, 122.9, 120.5, 108.9, 74.9, 67.8, 32.5, 28.0, 26.7, 25.6.

HRMS (ESI): Calculated for C_18_H_19_O_3_NaBr ([M+Na]^+^): 385.0410 and 387.0389; found 385.0405 and 387.0386.

(*S*)-4’-bromo-3-((*S*)-2,2-Dimethyl-1,3-dioxolan-4-yl)-2,3-dihydro-[1,1’-biphenyl]-4-carbaldehyde(24a).

Catalyst **9** used.

Yield: 45% (163 mg, 0.45 mmol).

[α]_D_^25^ = -23.1 (c = 1.10, CHCl_3_), this optical rotation was obtained from chromatographed fraction.

[α]_D_^25^ = -23.2 (c = 0.10, CHCl_3_), this optical rotation was obtained from a solution of crystals.

IR (film): 2987, 2875, 2718, 1668, 1171, 1072, 853, 813 cm^-1^.

^1^H NMR (200 MHz, CDCl_3_): δ = 9.60 (1H, s), 7.53 (2H, d, J = 8.6 Hz), 7.39 (2H, d, J = 8.6 Hz), 6.99 (1H, d, J = 6.0 Hz), 6.54 (1H, dd, J = 6.0, 2.3 Hz), 4.28 (1H, q, J = 6.3 Hz), 3.89 (1H, dd, J = 8.4, 6.3 Hz), 3.71 (1H, dd, J = 8.4, 6.3 Hz), 3.31 (1H, ddd, J = 9.1, 5.9, 3.2 Hz), 3.02–2.66 (2H, m), 1.39 (3H, s), 1.29 (3H, s).

^13^C NMR (50 MHz, CDCl_3_): δ = 192.2, 145.0, 144.0, 138.2, 136.5, 132.2, 127.5, 123.5, 119.9, 109.2, 76.0, 66.7, 32.0, 27.2, 26.5, 25.5.

HRMS (ESI): Calculated for C_18_H_20_O_3_Br ([M+H]^+^): 363.0590 and 365.0570; found 363.0596 and 365.0581.

(*R*)-4’-bromo-3-((*S*)-2,2-Dimethyl-1,3-dioxolan-4-yl)-2,3-dihydro-[1,1’-biphenyl]-4-carbaldehyde(24b).

Catalyst **10** used.

Yield: 45% (164 mg, 0.45 mmol).

[α]_D_^25^ = -27.3 (c = 0.02, CHCl_3_).

IR (film): 2984, 2872, 2718, 1668, 1169, 1072, 853, 815 cm^-1^.

^1^H NMR (200 MHz, CDCl_3_): δ = 9.56 (1H, s), 7.52 (2H, d, J = 8.6 Hz), 7.41 (2H, d, J = 8.6 Hz), 7.00 (1H, d, J = 5.8 Hz), 6.55 (1H, dd, J = 5.8, 2.8 Hz), 4.11–3.88 (1H, m), 3.79 (2H, dd, J = 6.3, 2.0 Hz), 3.20 (1H, d, J = 17.9 Hz), 3.08 (1H, t, J = 8.2 Hz), 2.70 (1H, ddd, J = 17.9, 8.2, 2.8 Hz), 1.35 (3H, s), 1.27 (3H, s).

^13^C NMR (50 MHz, CDCl_3_): δ = 192.5, 146.0, 145.7, 138.6, 136.5, 132.1 (2), 127.9 (2), 123.4, 120.1, 109.1, 75.2, 68.1, 32.8, 28.1, 27.0, 25.8.

HRMS (ESI): Calculated for C_18_H_20_O_3_Br ([M+H]^+^): 363.0590 and 365.0570; found 363.0594 and 365.0582.

(*S*)-3-((*S*)-2,2-Dimethyl-1,3-dioxolan-4-yl)-4’-nitro-2,3-dihydro-[1,1’-biphenyl]-4-carbaldehyde(25a).

Catalyst **9** used.

Yield: 90% (296 mg, 0.90 mmol).

[α]_D_^25^ = -19.5 (c = 0.02, CHCl_3_).

IR (film): 2983, 2931, 1668, 1554, 1172, 756 cm^-1^.

^1^H NMR (200 MHz, CDCl_3_): δ = 9.64 (1H, s), 8.25 (2H, d, J = 9.1 Hz), 7.66 (2H, d, J = 9.1 Hz), 7.03 (1H, d, J = 5.9 Hz), 6.67 (1H, d, J = 5.9 Hz), 4.33–4.24 (1H, m), 3.92 (1H, dd, J = 8.5, 6.4 Hz), 3.73 (1H, dd, J = 8.5, 6.4 Hz), 3.37–3.28 (1H, m), 2.94–2.90 (2H, m), 1.38 (3H, s), 1.28 (3H, s).

^13^C NMR (50 MHz, CDCl_3_): δ = 192.3, 147.9, 145.7, 143.5, 143.0, 137.7, 126.6 (2), 124.3 (2), 122.8, 109.4, 76.1, 66.7, 32.0, 27.4, 26.4, 25.3.

HRMS (ESI): Calculated for C_18_H_19_NO_5_Na ([M+Na]^+^): 352.1155; found 352.1151.

(*R*)-3-((*S*)-2,2-Dimethyl-1,3-dioxolan-4-yl)-4’-nitro-2,3-dihydro-[1,1’-biphenyl]-4-carbaldehyde(25b).

Catalyst **10** used.

Yield: 88% (290 mg, 0.88 mmol).

[α]_D_^25^ = -27.6 (c = 0.04, CHCl_3_).

IR (film): 2983, 2931, 1668, 1554, 1172, 756 cm^-1^.

^1^H NMR (200 MHz, CDCl_3_): δ = 9.61 (1H, s), 8.25 (2H, d, J = 8.8 Hz), 7.69 (2H, d, J = 8.8 Hz), 7.04 (1H, d, J = 5.8 Hz), 6.69 (1H, dd, J = 5.8, 2.9 Hz), 4.06–3.92 (1H, m), 3.90–3.72 (2H, m), 3.25 (1H, dd, J = 17.7, 1.5 Hz), 3.12 (1H, td, J = 8.1, 1.5 Hz), 2.77 (1H, ddd, J = 17.7, 8.1, 3.0 Hz), 1.35 (3H, s), 1.27 (3H, s).

^13^C NMR (50 MHz, CDCl_3_): δ = 192.5, 147.8, 146.1, 144.7, 144.3, 137.7, 127.1 (2), 124.2 (2), 122.9, 109.2, 75.4, 68.0, 32.8, 28.0, 27.0, 25.7.

HRMS (ESI): Calculated for C_18_H_19_NO_5_Na ([M+Na]^+^): 352.1155; found 352.1150.

(1*S*)-1-((*S*)-2,2-Dimethyl-1,3-dioxolan-4-yl)-1,5,6,7,8,8a-hexahydronaftalen-2-carbaldehyde(26).

Catalyst **9** used.

Yield: 4% (11 mg, 0.04 mmol).

[α]_D_^25^ = -198.5 (c = 0.33, CHCl_3_).

IR (film): 2930, 2855, 1672, 1582, 1059 cm^-1^

^1^H NMR (200 MHz, CDCl_3_): δ = 9.45 (1H, s), 6.73 (1H, d, J = 5.8 Hz), 5.84 (1H, d, J = 5.8 Hz), 4.34–4.27 (1H, m), 3.83 (1H, dd, J = 8.6, 6.8 Hz), 3.67 (1H, dd, J = 8.6, 6.8 Hz), 2.95–2.85 (1H, m), 2.60–2.40 (1H, m), 2.30–1.27 (8H, m), 1,25 (6H, s).

^13^C NMR (50 MHz, CDCl_3_): δ = 193.0, 159.4, 144.9, 133.7, 114.6, 109.0, 66.3, 40.3, 38.5, 38.1, 37.1, 32.0, 29.9, 27.4, 26.4, 25.3.

HRMS (ESI): Calculated for C_16_H_22_O_3_Na ([M+Na]^+^): 285.1461; found 285.1466.

## Conclusions

A new method for the synthesis of photoprotective chiral cyclohexadienals is described. The Jørgensen-Hayashi catalyst produced a good yield of these compounds by using a chiral α,β-unsaturated aldehyde, **2**. Further reactivity of the corresponding cyclohexadienals is under study.

According to the UV-Vis spectra of **4a**, **20b**, **21b**, **22b**, **22a** and **23b** it can be concluded that the cyclohexadienals containing systems with upper conjugation (**21b**, **22b**, **23a** and **23b**) present better absorbance properties than low conjugation cyclohexadienals **4a**, **20b**. In addition, the influence of the aryl substituent provides an important tool for modulating maximum absorbance. In this work, the influence of *p*-methylphenyl, *m*-bromophenyl and phenyl substituent on the cyclohexadienal backbone is shown, where the phenyl and *m*-bromophenyl substituents prove to be the best choice for UVA-filters and UVB-filters, respectively.

## Supporting information

S1 File NMR and IR data. (DOCX)

S2 File Experimental procedure for the synthesis of α,β-aldehyde intermediates. (DOCX)

S3 File X-Ray crystallographic data. (DOCX)

S4 File UV-Vis spectra. (DOCX)

## References

[1] Bertelsen S, Jorgensen KA. Organocatalysis-after the gold rush. Chem Soc Rev. 2009;38(8):2178–89. doi: 10.1039/b903816g 19623342 10.1039/b903816g

[2] Mukherjee S, Yang JW, Hoffmann S, List B. Asymmetric Enamine Catalysis. Chem Rev. 2007;107(12):5471–569. doi: 10.1021/cr0684016 18072803 10.1021/cr0684016

[3] Scheffler U, Mahrwald R. Recent Advances in Organocatalytic Methods for Asymmetric C-C Bond Formation. Chem Eur J. 2013;19(43):14346–96. doi: 10.1002/chem.201301996 24115407 10.1002/chem.201301996

[4] Maruoka K, List B, Yamamoto H, Gong L-Z. Organocatalysis: a web collection. Chem Commun. 2012;48(87):10703– and following articles. 10.1039/c2cc90327j 23019573

[5] Ahrendt KA, Borths CJ, MacMillan DWC. New Strategies for Organic Catalysis: The First Highly Enantioselective Organocatalytic Diels−Alder Reaction. J Am Chem Soc. 2000;122(17):4243–4.

[6] Pezzati B, Chellat MF, Murphy JJ, Besnard C, Reginato G, Stephens JC, et al Organocatalytic Asymmetric Annulation of 1,3-Bis(alkoxycarbonyl)buta-1,3-dienes and Aldehydes. Org Lett. 2013;15(12):2950–3. doi: 10.1021/ol401042b 23731393 10.1021/ol401042b

[7] Murphy JJ, Quintard A, McArdle P, Alexakis A, Stephens JC. Asymmetric Organocatalytic 1,6-Conjugate Addition of Aldehydes to Dienic Sulfones. Angew Chem Int Ed. 2011;50(22):5095–8. 10.1002/anie.201100804 21538747

[8] Ye L-W, Wang S-B, Wang Q-G, Sun X-L, Tang Y, Zhou Y-G. Asymmetric tandem Michael addition-ylide olefination reaction for the synthesis of optically active cyclohexa-1,3-diene derivatives. Chem Commun. 2009(21):3092–4. 10.1039/b900048h 19462097

[9] Chang M-Y, Chan C-K, Lin S-Y, Wu M-H. One-pot synthesis of multifunctionalized m-terphenyls. Tetrahedron. 2013;69(46):9616–24.

[10] Wang Z-Y, Wong W-T, Yang D. Organocatalyzed Asymmetric Synthesis of Dihydrodibenzofurans Based on a Dienamine Process. Organic Letters. 2013;15(19):4980–3. doi: 10.1021/ol402288y 24040890 10.1021/ol402288y

[11] Loupy A, Maurel F, Sabatié-Gogová A. Improvements in Diels–Alder cycloadditions with some acetylenic compounds under solvent-free microwave-assisted conditions: experimental results and theoretical approaches. Tetrahedron. 2004;60(7):1683–91.

[12] Serebryakov EP, Shcherbakov MA, Gamalevich GD, Struchkova MI. New ways to determine the absolute configurations of alkyl 6-R-cyclohexa-1,3-dienecarboxylates. Russ Chem Bull. 2003;52(3):734–9.

[13] Serebryakov EP, Nigmatov AG, Shcherbakov MA, Struchkova MI. The effects of the nature of catalyst and of the solvent on the stereoselectivity in amine-catalyzed asymmetric synthesis of substituted cyclohexa-1,3-dienes from prenal and monoesters of ylidenemalonic acids. Russ Chem Bull. 1998;47(1):82.

[14] Nigmatov AG, Serebryakov EP. Catalytic asymmetric synthesis of polysubstituted cyclohexa-1,3-dienes from β-branched α,β-alkenals. Russ Chem Bull. 1996;45(3):623–9.

[15] Nigmatov AG, Kornilova IN, Serebryakov EP. Synthesis of polysubstituted 1,3-cyclohexadienes from β-branched α,β-alkenals and monoesters of ylidenemalonic acids. Russ Chem Bull. 1996;45(1):144–52.

[16] Nigmatov AG, Serebryakov EP. Catalytic asymmetric synthesis of 6-substituted derivatives of 1,3-cyclohexadienecarboxylic acid. Russ Chem Bull. 1993;42(1):213–.

[17] Hong B-C, Wu M-F, Tseng H-C, Huang G-F, Su C-F, Liao J-H. Organocatalytic Asymmetric Robinson Annulation of α,β-Unsaturated Aldehydes: Applications to the Total Synthesis of (+)-Palitantin. J Org Chem. 2007;72(22):8459–71. doi: 10.1021/jo701477v 17919000 10.1021/jo701477v

[18] Hong B-C, Tseng H-C, Chen S-H. Synthesis of aromatic aldehydes by organocatalytic [4+2] and [3+3] cycloaddition of α,β-unsaturated aldehydes. Tetrahedron. 2007;63(13):2840–50.

[19] Hong B-C, Wu M-F, Tseng H-C, Liao J-H. Enantioselective Organocatalytic Formal [3 + 3]-Cycloaddition of α,β-Unsaturated Aldehydes and Application to the Asymmetric Synthesis of (−)-Isopulegol Hydrate and (−)-Cubebaol. Org Lett. 2006;8(11):2217–20. doi: 10.1021/ol060486+ 16706490 10.1021/ol060486+

[20] Bench BJ, Liu C, Evett CR, Watanabe CMH. Proline Promoted Synthesis of Ring-Fused Homodimers: Self-Condensation of α,β-Unsaturated Aldehydes. J Org Chem. 2006;71(25):9458–63. doi: 10.1021/jo061763t 17137373 10.1021/jo061763t

[21] Bench BJ, Tichy SE, Perez LM, Benson J, Watanabe CMH. Synthesis and cellular effects of cycloterpenals: Cyclohexadienal-based activators of neurite outgrowth. Bioorg Med Chem. 2008;16(16):7573–81. doi: 10.1016/j.bmc.2008.07.030 18678500 10.1016/j.bmc.2008.07.030

[22] Yamada S-i, Shibasaki M, Terashima S. A biogenetic-type asymmetric cyclization syntheses of optically active α-cyclocitral and trans-α-damascone. Tetrahedron Lett. 1973;14(5):381–4.

[23] Asato AE, Watanabe C, Li X-Y, Liu RSH. The proline and β-lactoglobulin mediated asymmetric self-condensation of β-ionylideneacetaldehyde, retinal and related compounds. Tetrahedron Lett. 1992;33(22):3105–8.

[24] Coran M. H. Watanabe, Bench BJ, inventorsSubstituted Cyclohexadienals—Syntheses and Applications. USA patent US 20070232813 A1. 2007 4 Oct 2007.

[25] Pena J, Moro RF, Basabe P, Marcos IS, Diez D. Solvent free l-proline-catalysed domino Knoevenagel/6[small pi]-electrocyclization for the synthesis of highly functionalised 2H-pyrans. RSC Advances. 2012;2(21):8041–9.

[26] Peters L, Wright AD, Kehraus S, Gündisch D, Tilotta MC, König GM. Prenylated Indole Alkaloids from Flustra foliacea with Subtype Specific Binding on NAChRs. Planta Med. 2004;70(10):883–6. doi: 10.1055/s-2004-832610 15490312 10.1055/s-2004-832610

[27] Fishkin NE, Sparrow JR, Allikmets R, Nakanishi K. Isolation and characterization of a retinal pigment epithelial cell fluorophore: An all-trans-retinal dimer conjugate. PNAS. 2005;102(20):7091–6. doi: 10.1073/pnas.0501266102 15870200 10.1073/pnas.0501266102 PMC1129110

[28] Buchanan GS, Dai H, Hsung RP, Gerasyuto AI, Scheinebeck CM. Asymmetric Aza-[3 + 3] Annulation in the Synthesis of Indolizidines: An Unexpected Reversal of Regiochemistry. Org Lett. 2011;13(16):4402–5. doi: 10.1021/ol2017438 21786757 10.1021/ol2017438 PMC3155627

[29] Sydorenko N, Hsung RP, Vera EL. Torquoselective 6π-Electron Electrocyclic Ring Closure of 1-Azatrienes Containing Acyclic Chirality at the C-Terminus. Org Lett. 2006;8(12):2611–4. doi: 10.1021/ol060932t 16737326 10.1021/ol060932t

[30] Deng Y, Salomon RG. Total Synthesis of γ-Hydroxy-α,β-Unsaturated Aldehydic Esters of Cholesterol and 2-Lysophosphatidylcholine. J Org Chem. 1998;63(22):7789–94.

[31] Gu X, Sun M, Gugiu B, Hazen S, Crabb JW, Salomon RG. Oxidatively Truncated Docosahexaenoate Phospholipids: Total Synthesis, Generation, and Peptide Adduction Chemistry. J Org Chem. 2003;68(10):3749–61. doi: 10.1021/jo026721t 12737551 10.1021/jo026721t

[32] Dunlap NK, Mergo W, Jones JM, Carrick JD. A general procedure for a one-pot oxidative cleavage/Wittig reaction of glycols. Tetrahedron Lett. 2002;43(21):3923–5.

[33] Díez Martin D, San Feliciano SG, Marcos IS, Basabe P, Garrido NM, Urones JG. On the Stereoselectivity of the Synthesis of 1-Hydroxymethyl-4-phenylsulfonylbuta-1,3-dienes from β,γ-Unsaturated Sulfones. Synthesis. 2001;2001(07):1069–75.

[34] Schuch AP, Menck CFM. The genotoxic effects of DNA lesions induced by artificial UV-radiation and sunlight. J Photochem Photobiol B. 2010;99(3):111–6. doi: 10.1016/j.jphotobiol.2010.03.004 20371188 10.1016/j.jphotobiol.2010.03.004

[35] Maciel E, Felgueiras J, Silva EMP, Ricardo F, Moreira ASP, Melo T, et al Lipid remodelling in human melanoma cells in response to UVA exposure. Photochem Photobiol Sci. 2017;16(5):744–52. doi: 10.1039/c7pp00025a 28304067 10.1039/c7pp00025a

[36] He J, Long C, Huang Z, Zhou X, Kuang X, Liu L, et al PTEN Reduced UVB-Mediated Apoptosis in Retinal Pigment Epithelium Cells. Biomed Res Int. 2017;2017:11. 10.1155/2017/3681707 PMC5340936 28321407

[37] Girigalavicius M, Iani V, Juzeniene A. Layer Thickness of SPF 30 Sunscreen and Formation of Pre-vitamin D. Anticancer Res. 2016;36(3):1409–15. 26977044

[38] Osterwalder U, Herzog B. Sun protection factors: world wide confusion. Br J Dermatol. 2009;161:13–24. doi: 10.1111/j.1365-2133.2009.09506.x 19775352 10.1111/j.1365-2133.2009.09506.x

[39] Food and Drugs, Vol. 5 (Rep. num.: 21CRF352), 2016, FDA.

[40] Freitas JV, Praça FSG, Bentley MVLB, Gaspar LR. Trans-resveratrol and beta-carotene from sunscreens penetrate viable skin layers and reduce cutaneous penetration of UV-filters. Int J Pharm. 2015;484(1):131–7. 25724133 10.1016/j.ijpharm.2015.02.062

[41] Bos JD, Meinardi MMHM. The 500 Dalton rule for the skin penetration of chemical compounds and drugs. Exp Dermatol. 2000;9(3):165–9. 10839713 10.1034/j.1600-0625.2000.009003165.x

[42] Gustavsson Gonzalez H, Farbrot A, Larkö O. Percutaneous absorption of benzophenone-3, a common component of topical sunscreens. Clin Exp Dermatol. 2002;27(8):691–4. 12472548 10.1046/j.1365-2230.2002.01095.x

[43] Gonzalez H. Percutaneous absorption with emphasis on sunscreens. Photochem Photobiol Sci. 2010;9(4):482–8. doi: 10.1039/b9pp00149b 20354641 10.1039/b9pp00149b

[44] Chrétien MN, Heafey E, Scaiano JC. Reducing Adverse Effects from UV Sunscreens by Zeolite Encapsulation: Comparison of Oxybenzone in Solution and in Zeolites. Photochem Photobiol. 2010;86(1):153–61. doi: 10.1111/j.1751-1097.2009.00644.x 19930122 10.1111/j.1751-1097.2009.00644.x

[45] Krause M, Klit A, Blomberg Jensen M, Søeborg T, Frederiksen H, Schlumpf M, et al Sunscreens: are they beneficial for health? An overview of endocrine disrupting properties of UV-filters. Int J Androl. 2012;35(3):424–36. doi: 10.1111/j.1365-2605.2012.01280.x 22612478 10.1111/j.1365-2605.2012.01280.x

[46] Gong P, Yuan H, Zhai P, Xue Y, Li H, Dong W, et al Investigation on the degradation of benzophenone-3 by UV/H2O2 in aqueous solution. Chem Eng J. 2015;277:97–103.

[47] Urosa A, Marcos I, Díez D, Lithgow A, Plata G, Padrón J, et al Synthesis and Bioactivity of Luffarin I. Marine Drugs. 2015;13(4):2407 doi: 10.3390/md13042407 25903281 10.3390/md13042407 PMC4413218

[48] Zeng S, Kapur A, Patankar MS, Xiong MP. Formulation, Characterization, and Antitumor Properties of Trans- and Cis-Citral in the 4T1 Breast Cancer Xenograft Mouse Model. Pharm Res. 2015;32(8):2548–58. doi: 10.1007/s11095-015-1643-0 25673043 10.1007/s11095-015-1643-0 PMC4490114

[49] Crystal data for 24a: CCDC 976522. See https://www.ccdc.cam.ac.uk/services/structure_deposit/ for crystallographic data in .cif or other electronic format.

