# Supplementary material for: Diastereoselective synthesis of chiral 1,3-cyclohexadienals
Source: PLoS One. 2018 Feb 13;13(2):e0192113. doi: 10.1371/journal.pone.0192113 (PMC5810990; doi:10.1371/journal.pone.0192113)
Supplement: S1 File — (DOCX) [file pone.0192113.s001.docx]

**Diastereoselective Synthesis of Chiral 1,3-Cyclohexadienals**

Aitor Urosa^1¶^, Ignacio E. Tobal^1¶^, Ángela P. de la Granja^1^, M. Carmen Capitán^1^, R. F. Moro^1^, Isidro S. Marcos^1^, Narciso M. Garrido^1^, Francisca Sanz^2^, Emilio Calle^3^ and David Díez^1^*

^1^ Departamento de Química Orgánica, Facultad de Ciencias Químicas, Universidad de Salamanca, Salamanca. Spain.

^2^ Servicio de Difracción de Rayos X, Universidad de Salamanca, Salamanca, Spain.

^3^Departamento de Química Física, Facultad de Ciencias Químicas, Universidad de Salamanca, Salamanca. Spain.

*E-mail: [ddm@usal.es](mailto:ddm@usal.es)

¶ These authors contributed equally to this work.

**SUPPORTING INFORMATION 1**

**NMR and IR data**

**TABLE OF CONTENTS**

| **Content** | **Page** |
| --- | --- |
| ^1^H NMR of **3** | S4 |
| ^1^H NMR and ^13^C NMR of **4a** | S5 |
| IR of **4a** | S6 |
| ^1^H NMR and ^13^C NMR of **4b** | S7 |
| IR of **4b** | S8 |
| ^1^H NMR and ^13^C NMR of **11** | S9 |
| IR of **11** | S10 |
| ^1^H NMR and ^13^C NMR of **12** | S11 |
| IR of **12** | S12 |
| ROESY of **12** | S13 |
| ^1^H NMR of **14a** and **14b** | S14 |
| ^1^H NMR of **14** and **15a** | S15 |
| ^1^H NMR of **15b** and **15** | S16 |
| ^1^H NMR of **16a** and **16b** | S17 |
| ^1^H NMR of **16** and **17a** | S18 |
| ^1^H NMR of **17b** and **17** | S19 |
| ^1^H NMR of **18a** and **18b** | S20 |
| ^1^H NMR of **18** and **19a** | S21 |
| ^1^H NMR of **19b** and **19** | S22 |
| ^1^H NMR and ^13^C NMR of **20a** | S23 |
| IR of **20a** | S24 |
| ^1^H NMR and ^13^C NMR of **20b** | S25 |
| IR of **20b** | S26 |
| ^1^H NMR and ^13^C NMR of **21a** | S27 |
| IR of **21a** | S28 |
| ^1^H NMR and ^13^C NMR of **21b** | S29 |
| IR of **21b** | S30 |
| ^1^H NMR and ^13^C NMR of **22a** | S31 |
| IR of **22a** | S32 |
| ^1^H NMR and ^13^C NMR of **22b** | S33 |
| IR of **22b** | S34 |
| ^1^H NMR and ^13^C NMR of **23a** | S35 |
| IR of **23a** | S36 |
| ^1^H NMR and ^13^C NMR of **23b** | S37 |
| IR of **23b** | S38 |
| ^1^H NMR and ^13^C NMR of **24a** | S39 |
| IR of **24a** | S40 |
| ^1^H NMR and ^13^C NMR of **24b** | S41 |
| IR of **24b** | S42 |
| ^1^H NMR and ^13^C NMR of **25a** | S43 |
| IR of **25a** | S44 |
| ^1^H NMR and ^13^C NMR of **25b** | S45 |
| IR of **25b** | S46 |
| ^1^H NMR and ^13^C NMR of **26** | S47 |
| IR of **26** | S48 |

^1^H NMR of **3**

**
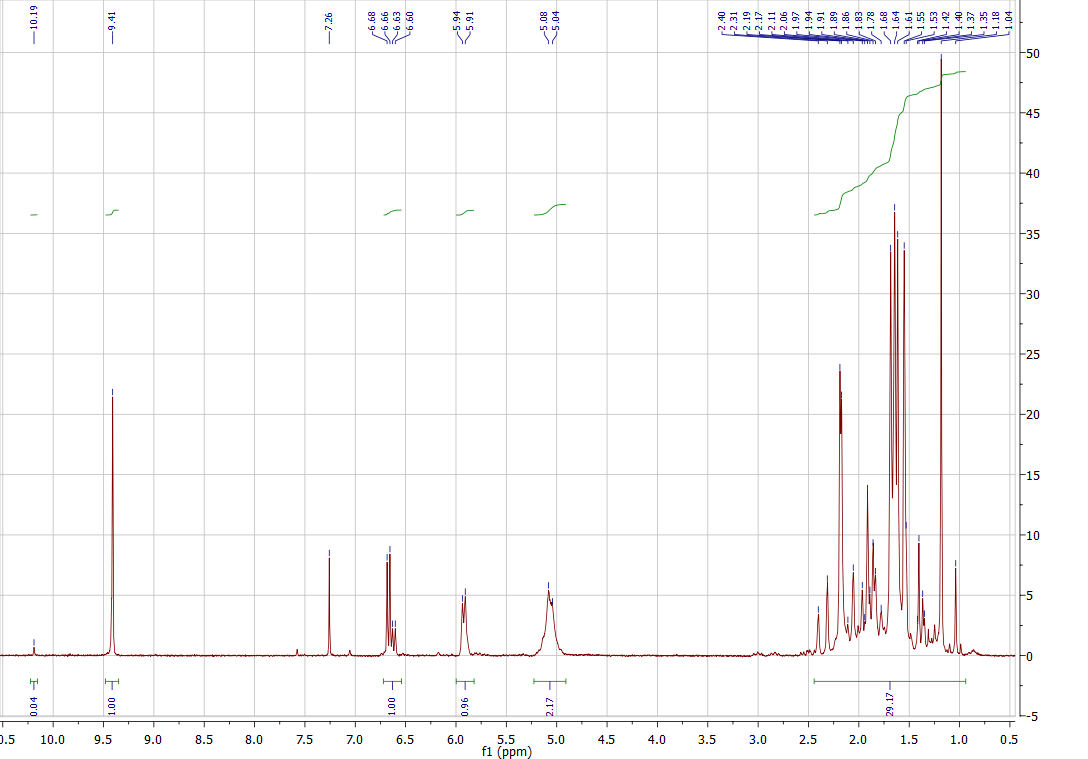
**

^1^H NMR and ^13^C NMR of **4a**

**
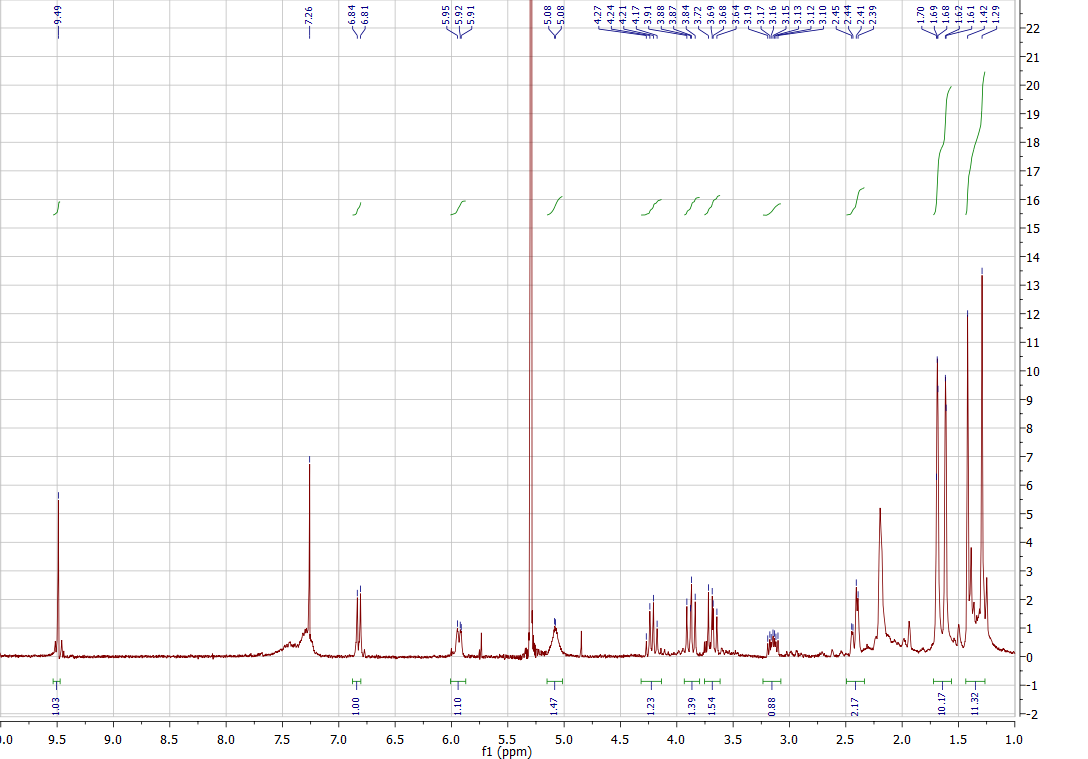
**

**
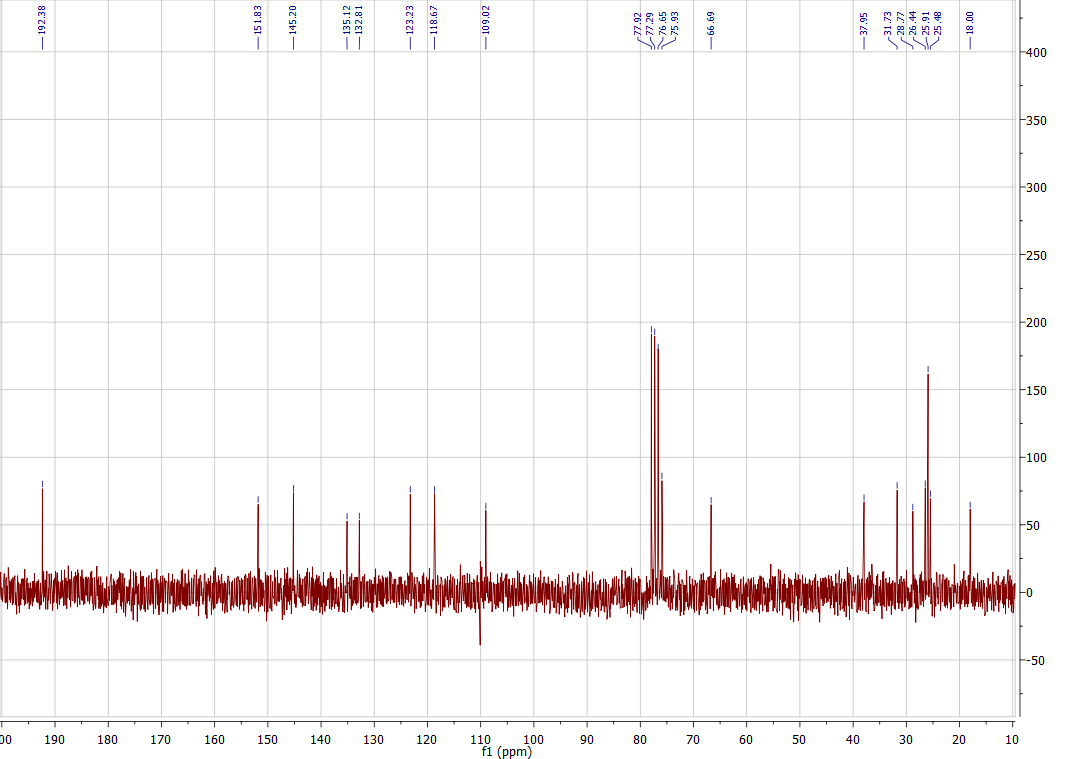
**

IR of **4a**

**
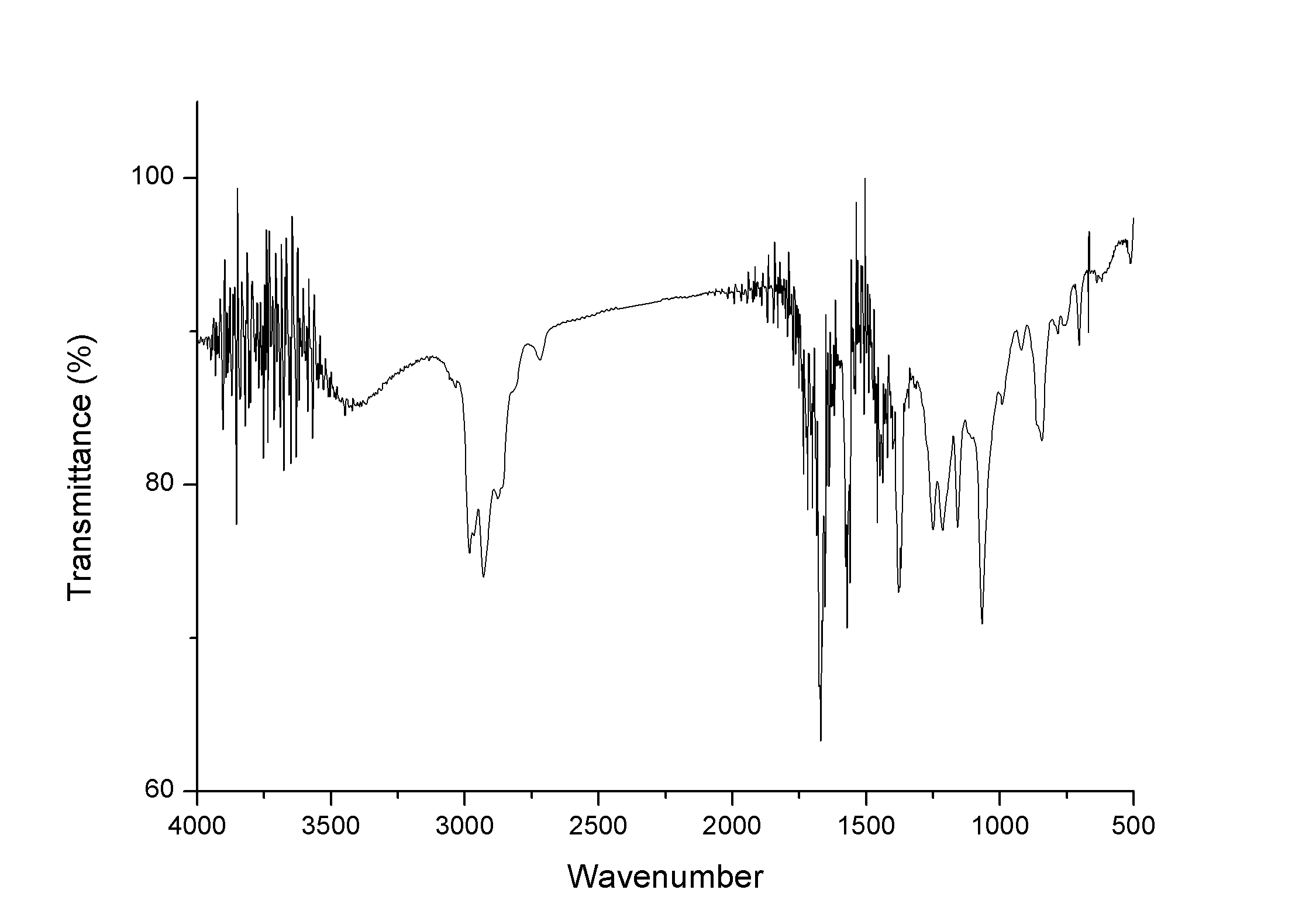
**

^1^H NMR and ^13^C NMR of **4b**

**
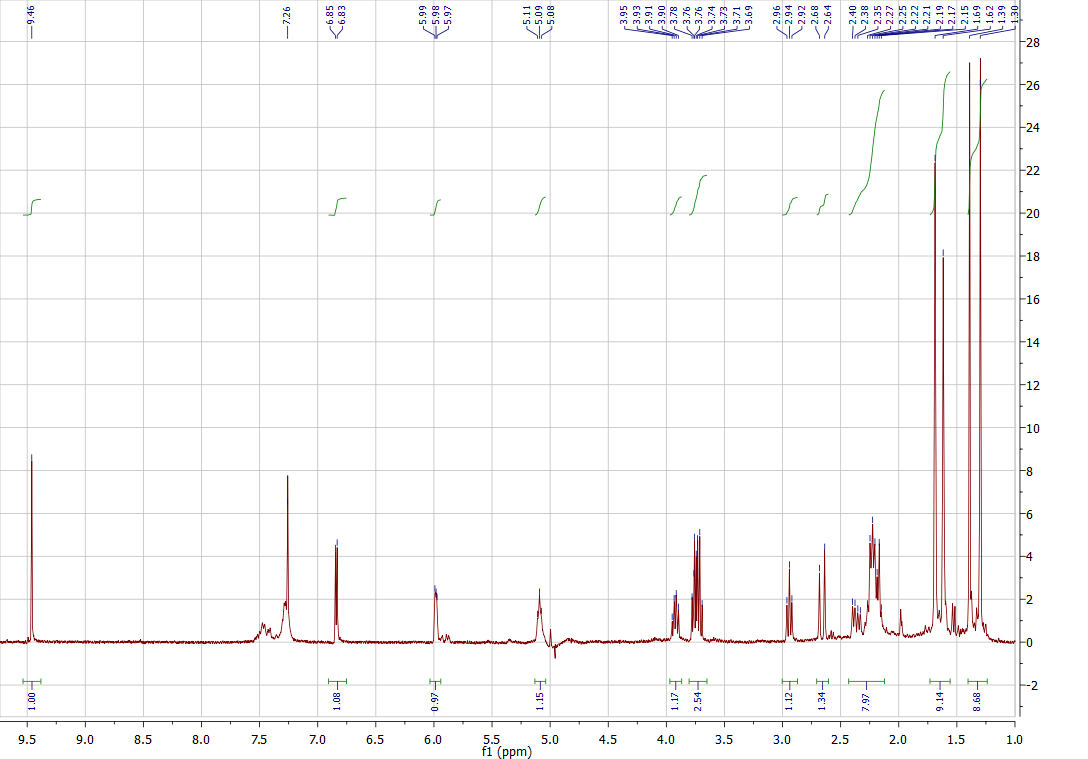
**

**
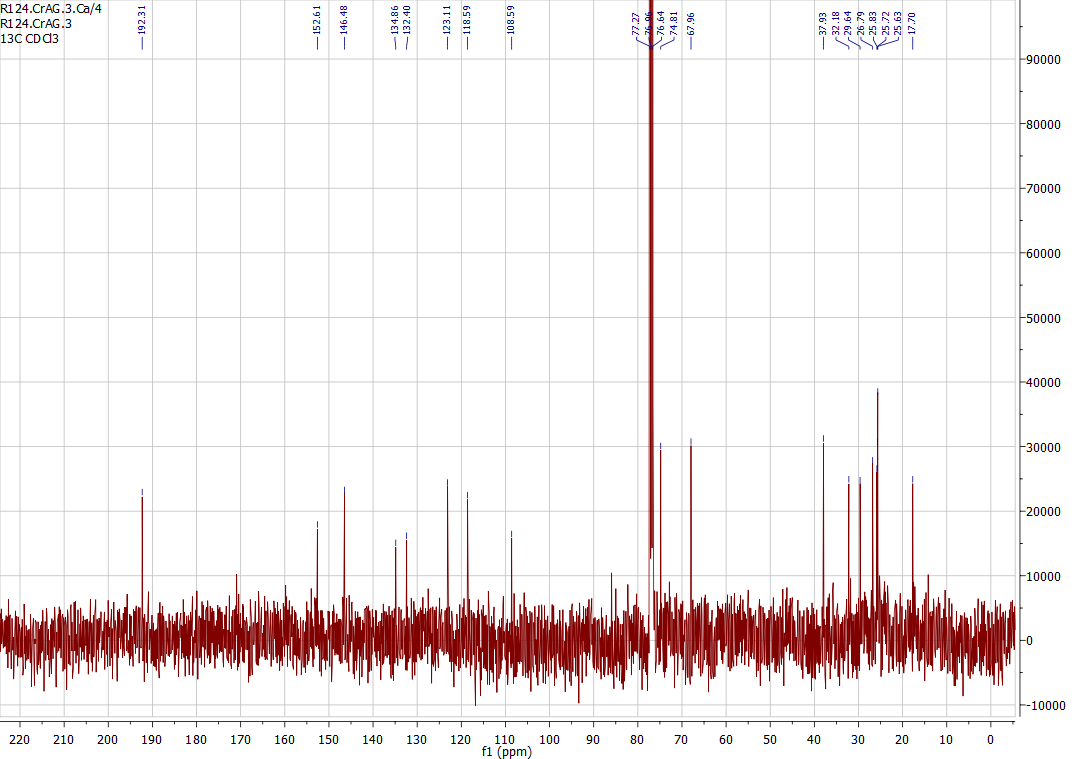
**

IR of **4b**

**
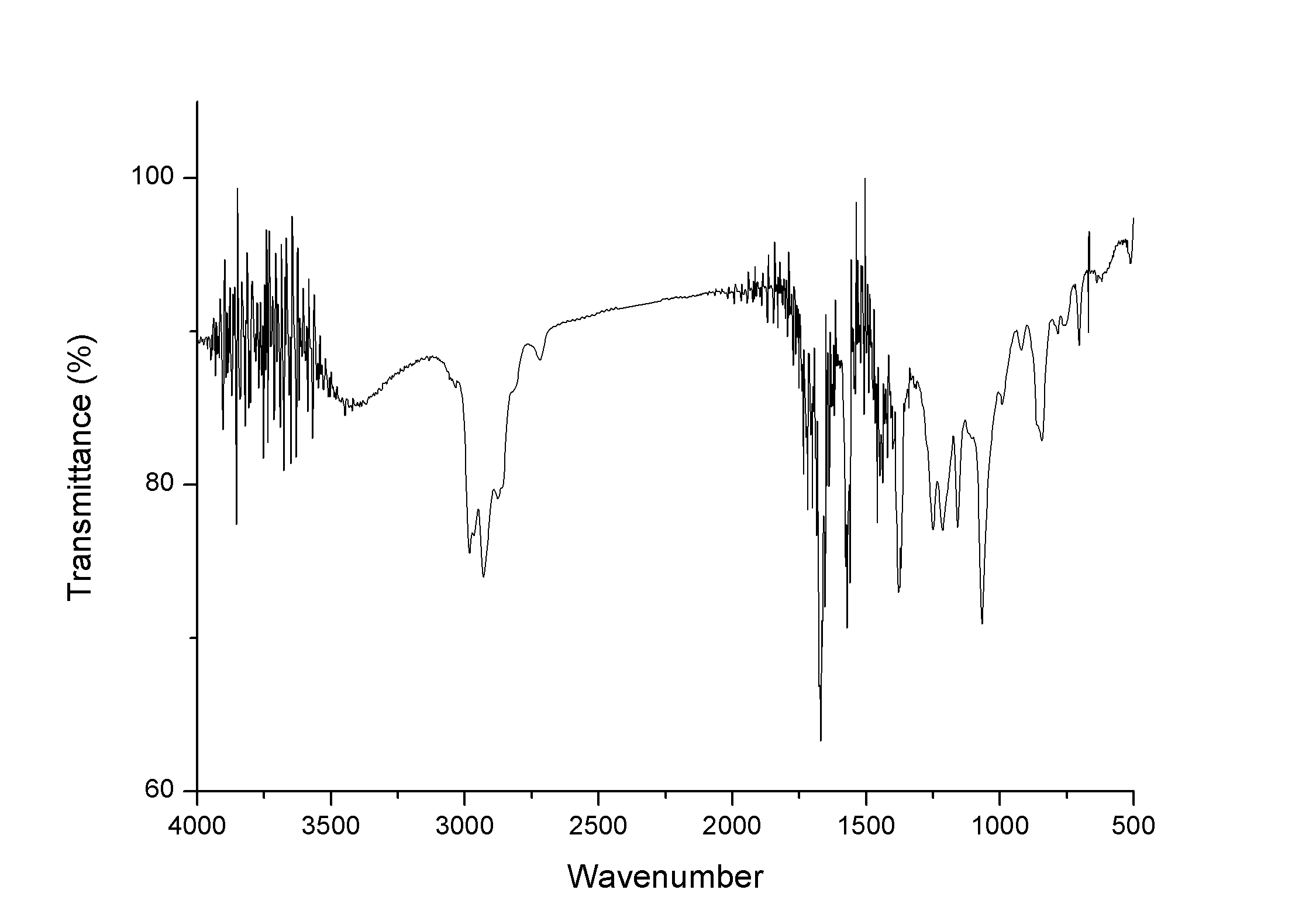
**

^1^H NMR and ^13^C NMR of **11**

**
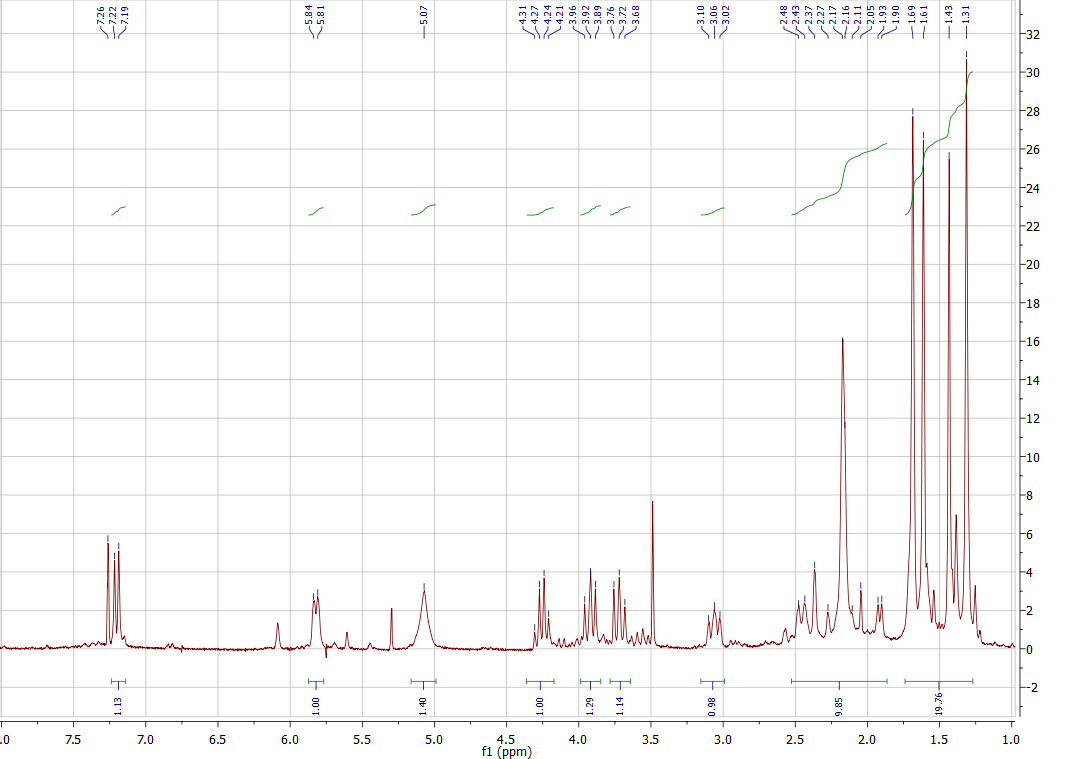
**

**
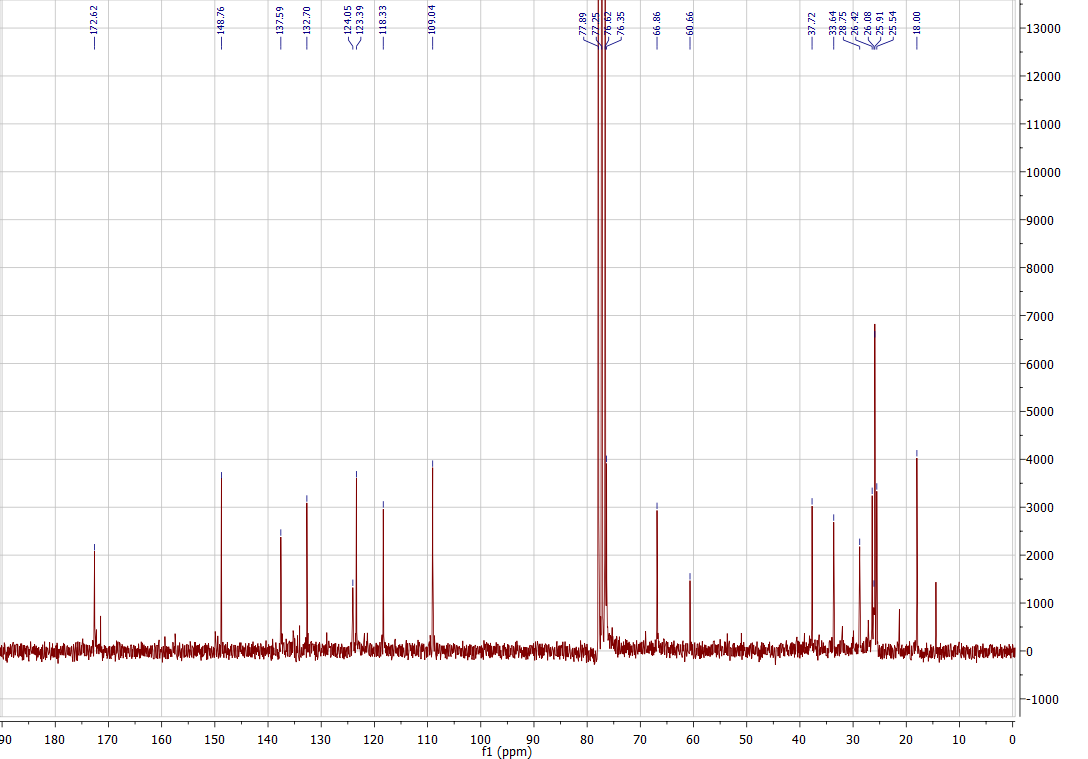
**

IR of **11**

**
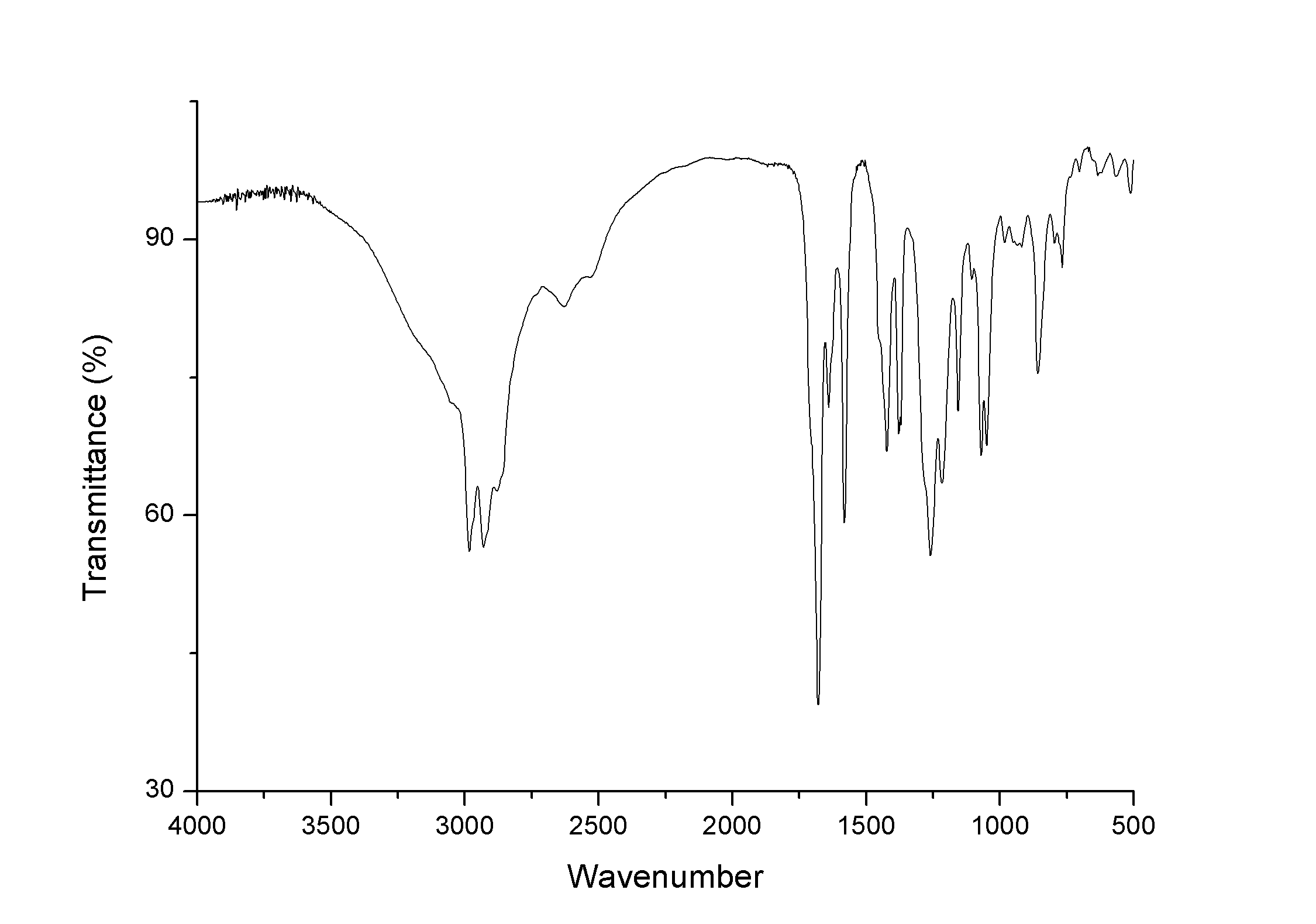
**

^1^H NMR and ^13^C NMR of **12**

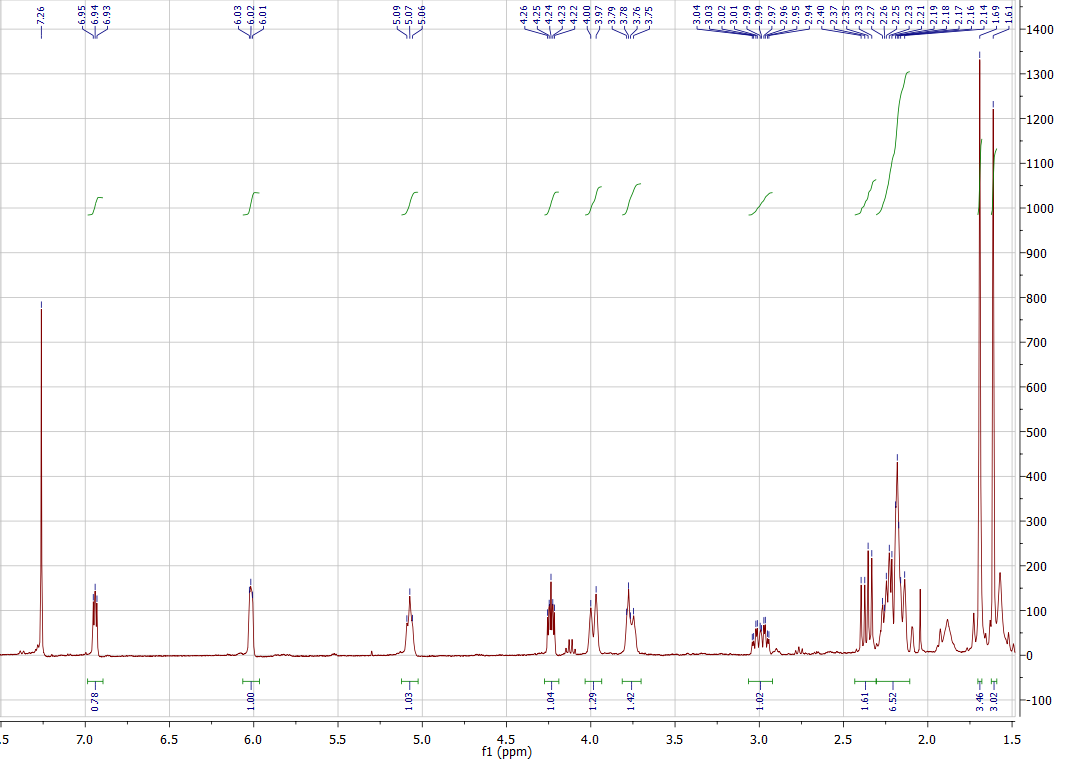


**
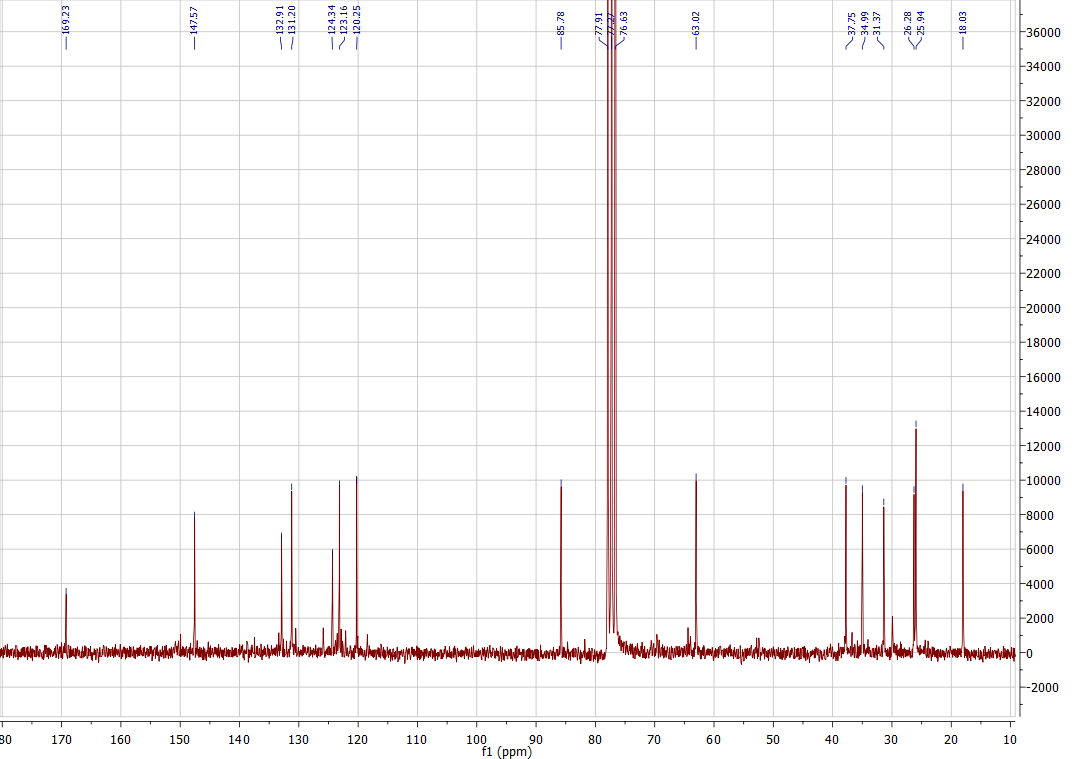
**

IR of **12**

**
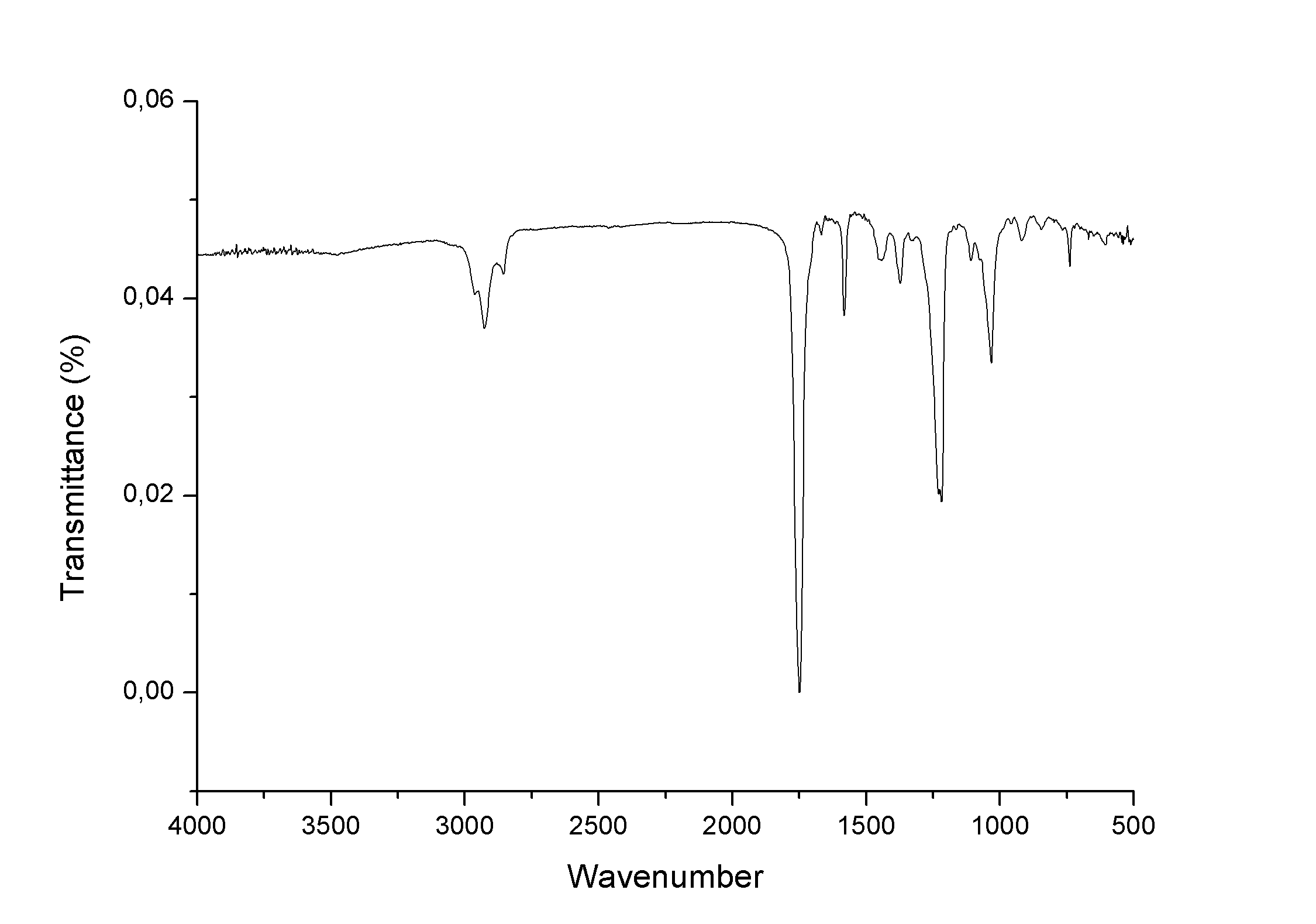
**

ROESY of **12**

**
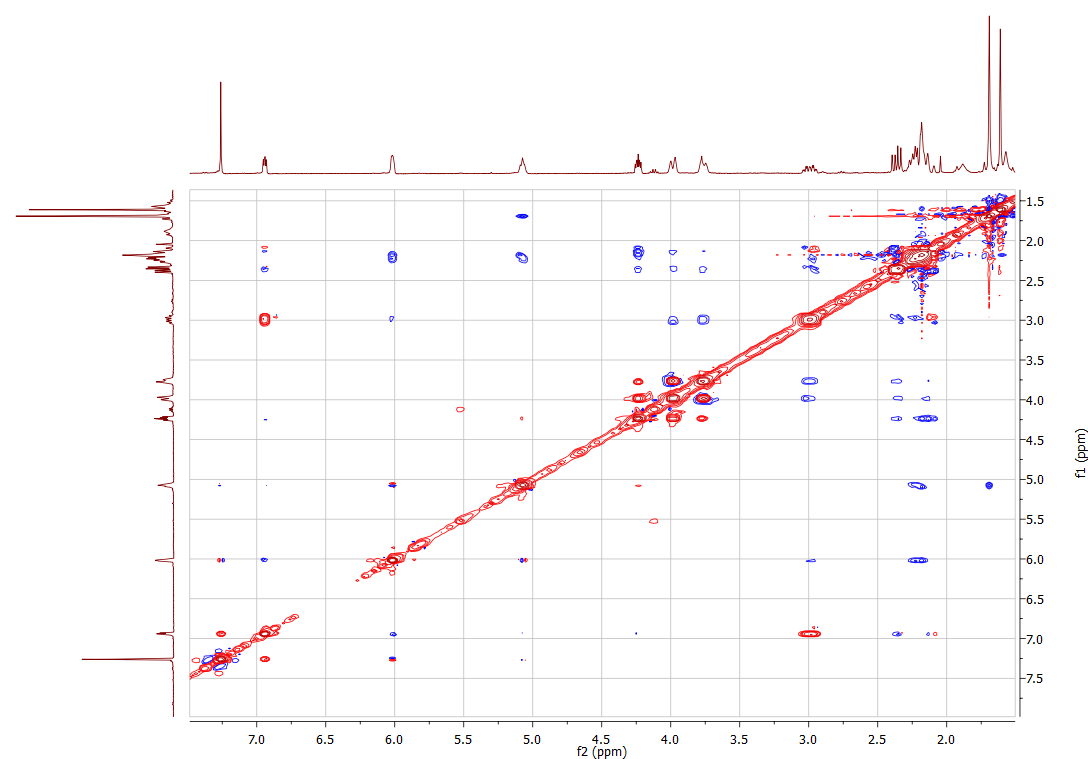
**

^1^H NMR of **14a**


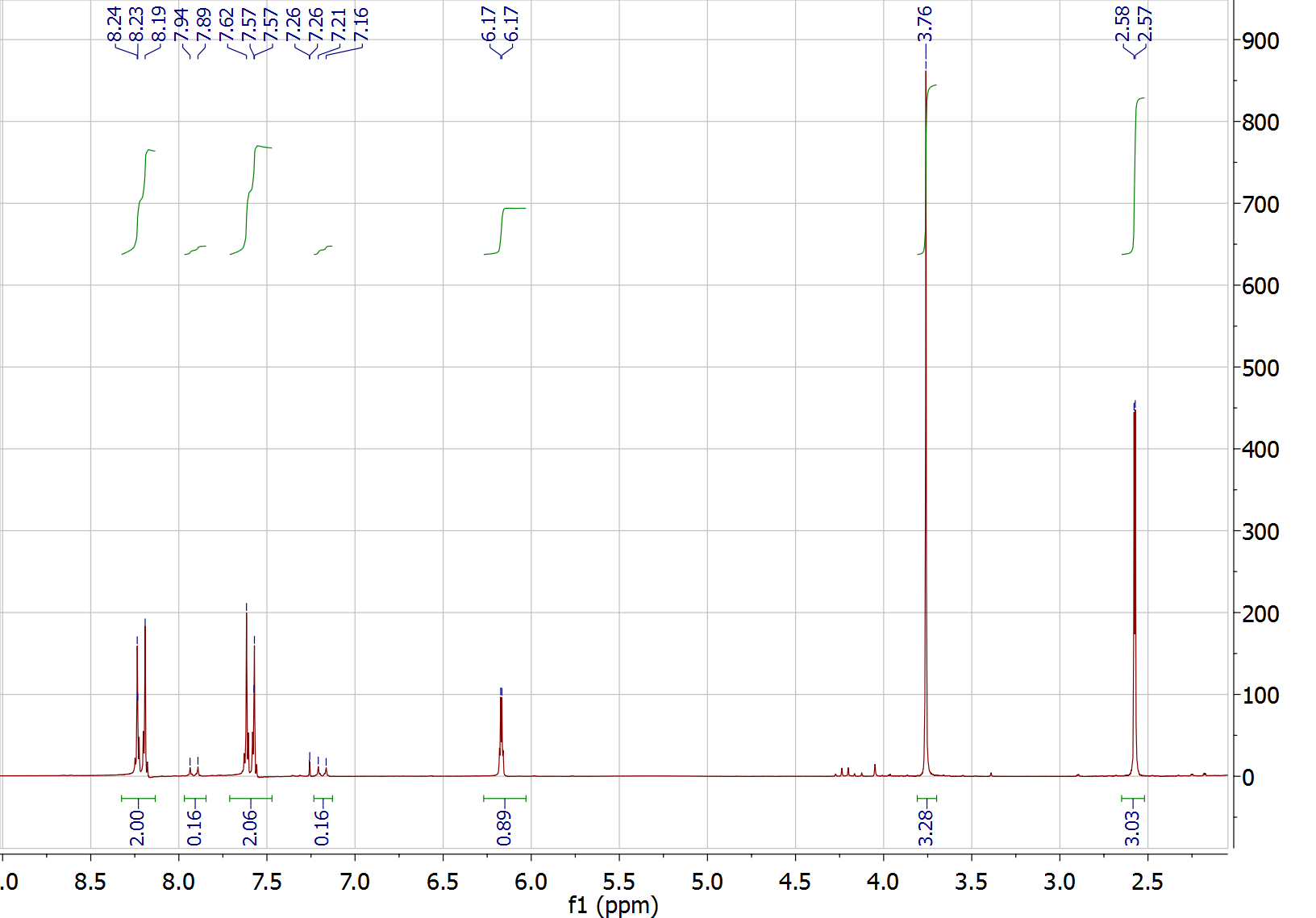


^1^H NMR of **14b**


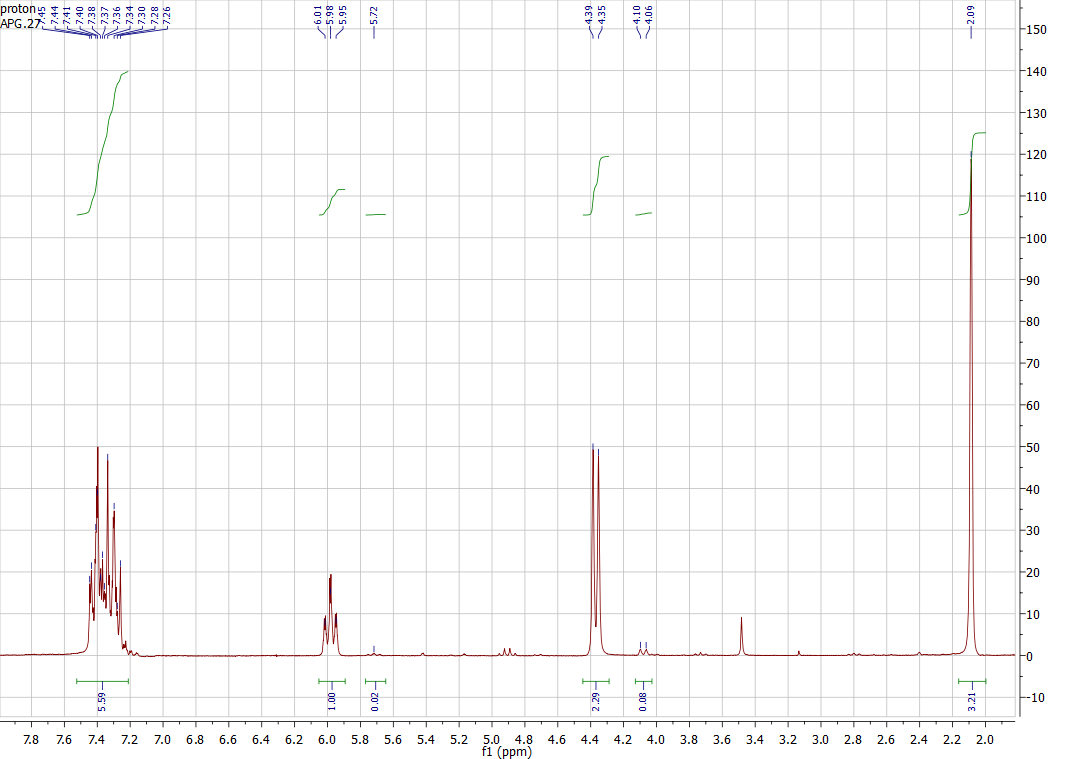


^1^H NMR of **14**


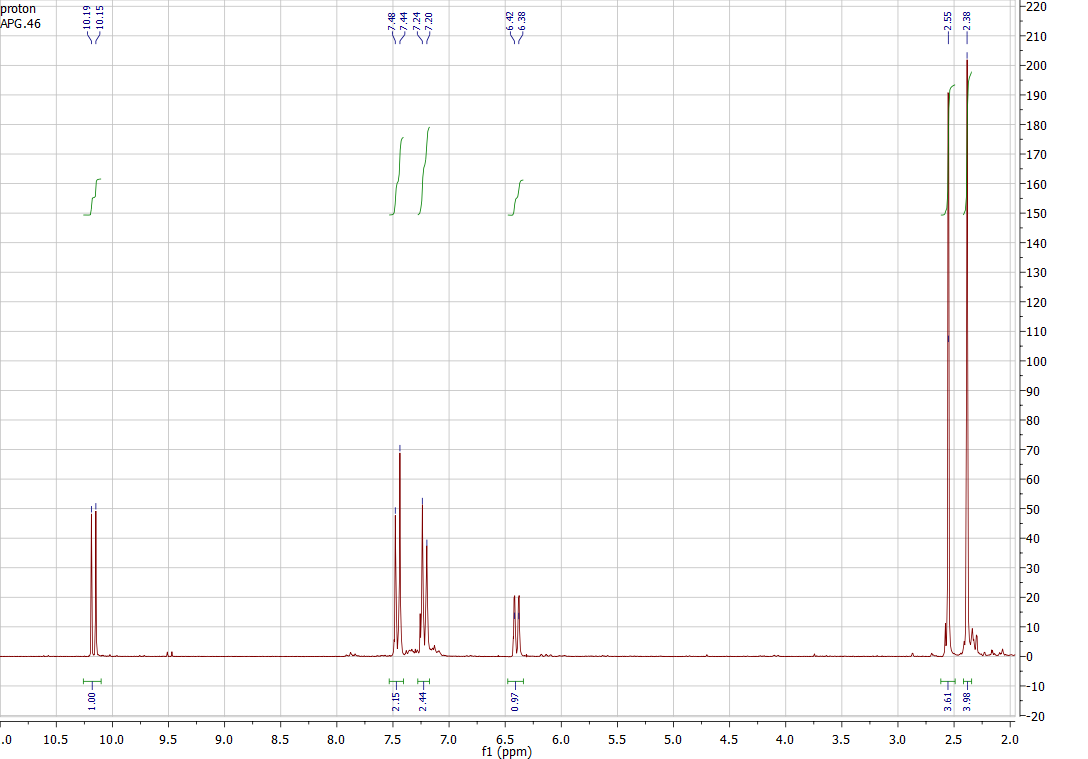


^1^H NMR of **15a**


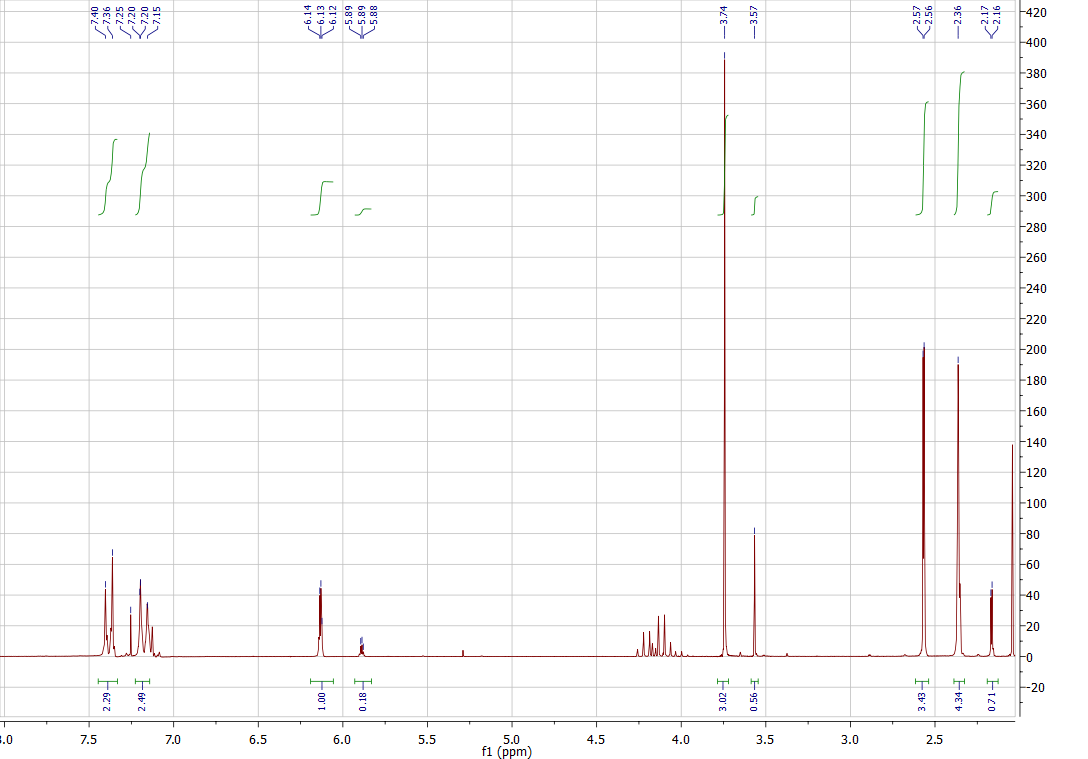


^1^H NMR of **15b**


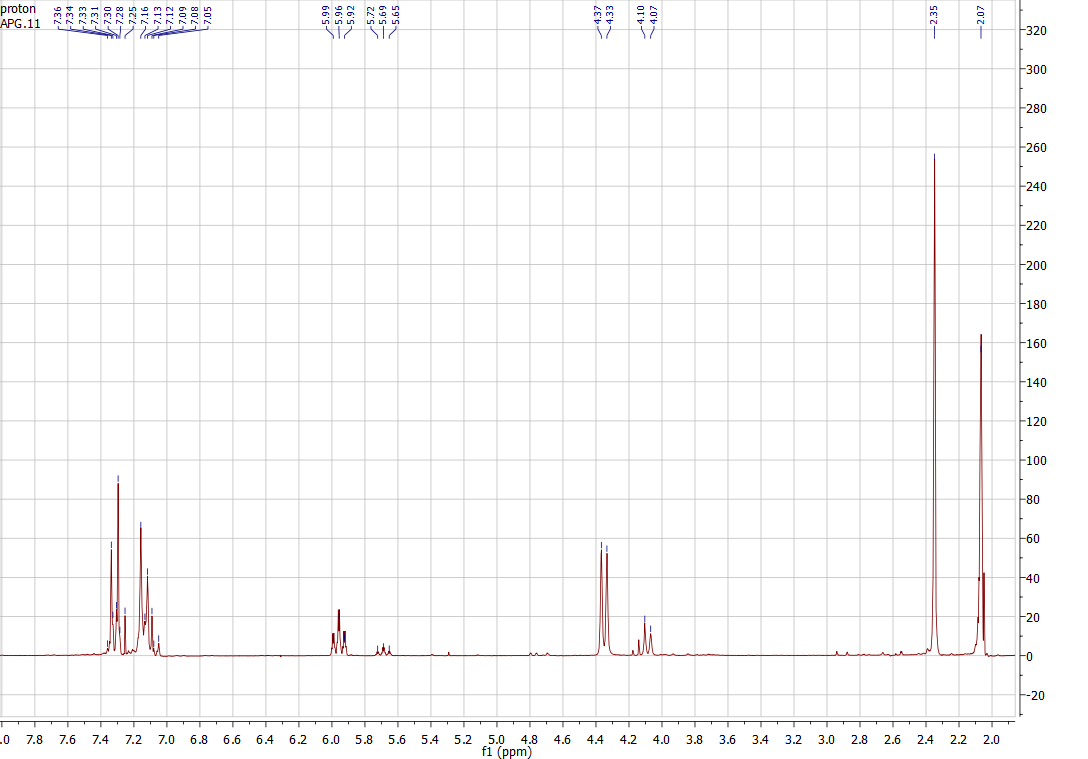


^1^H NMR of **15**


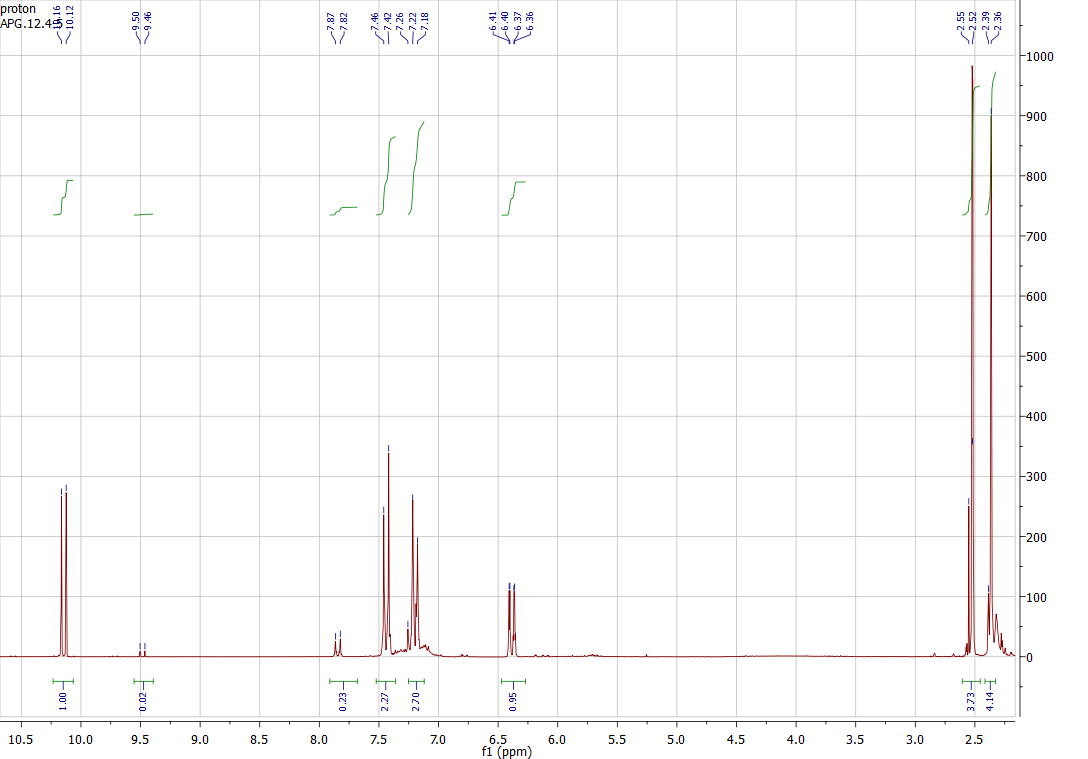


^1^H NMR of **16a**


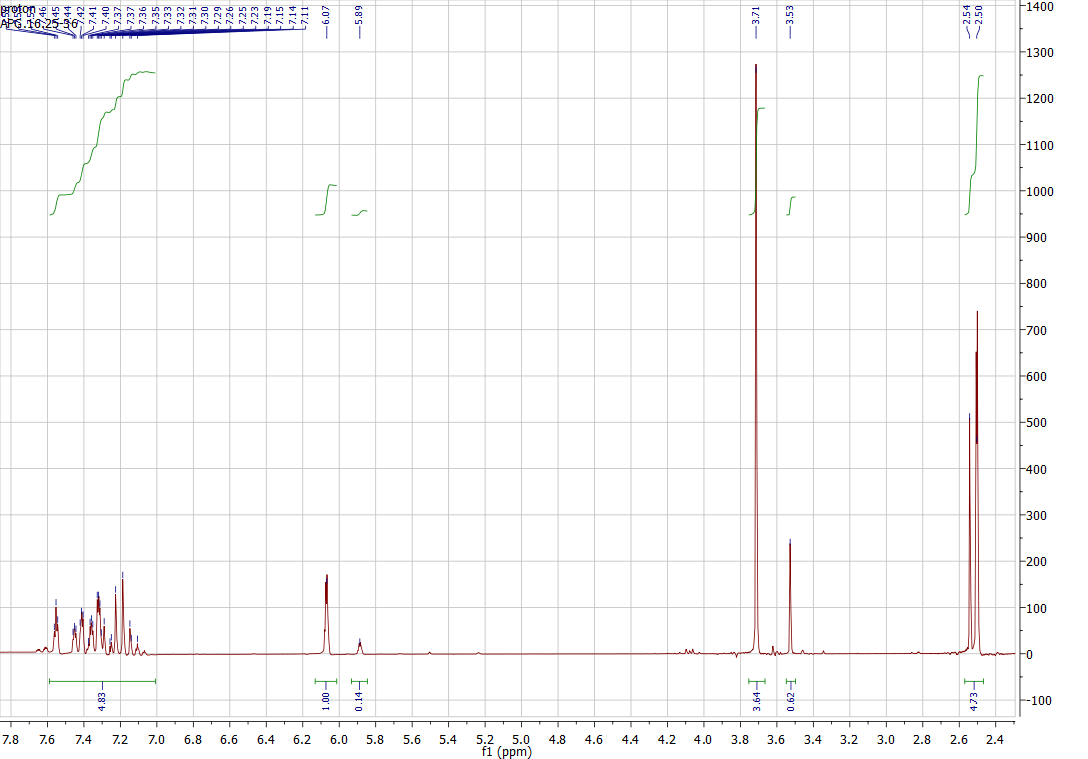


^1^H NMR of **16b**


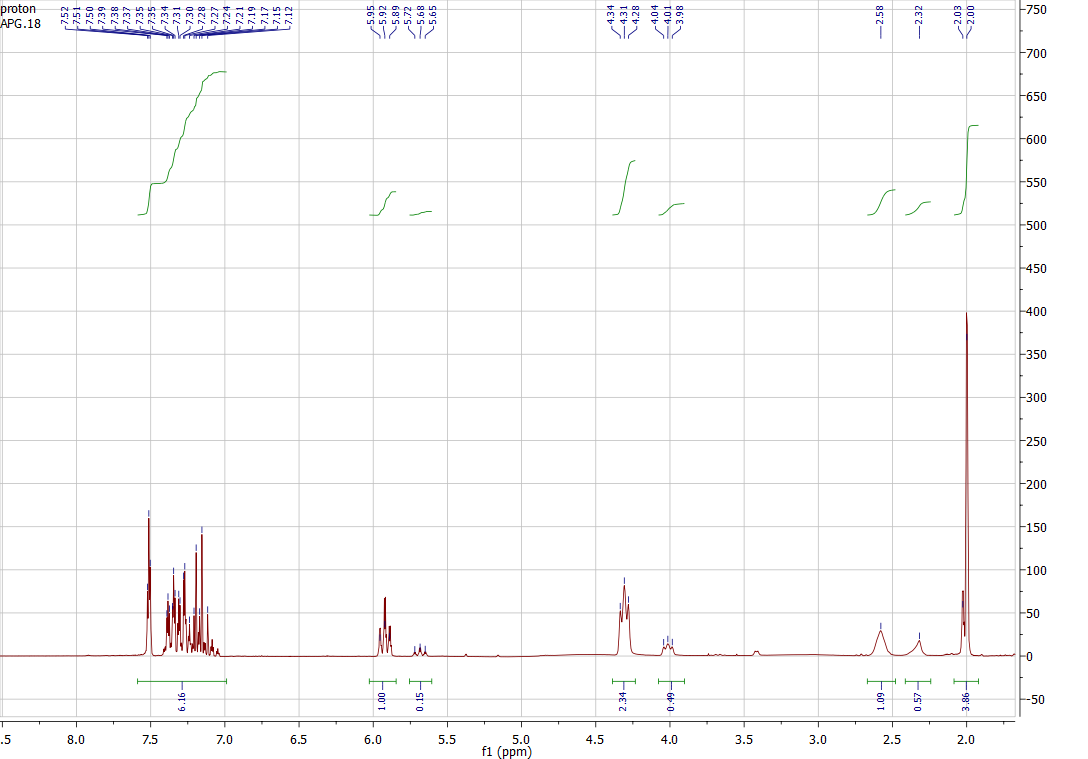


^1^H NMR of **16**


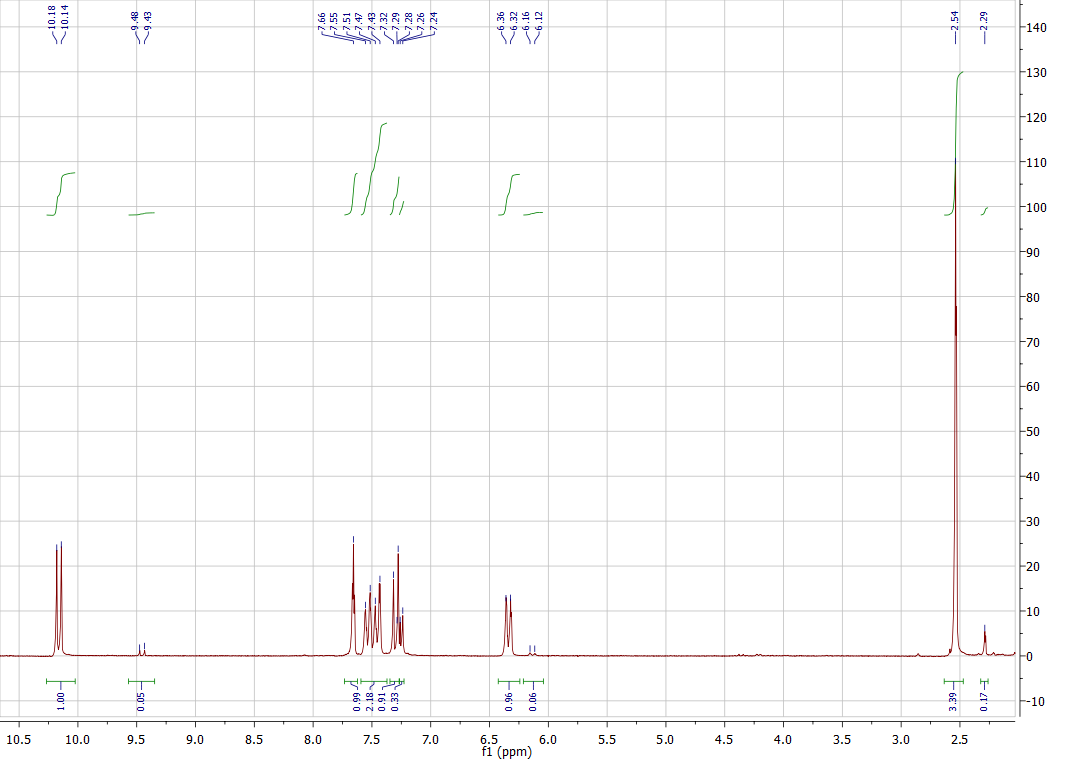


^1^H NMR of **17a**


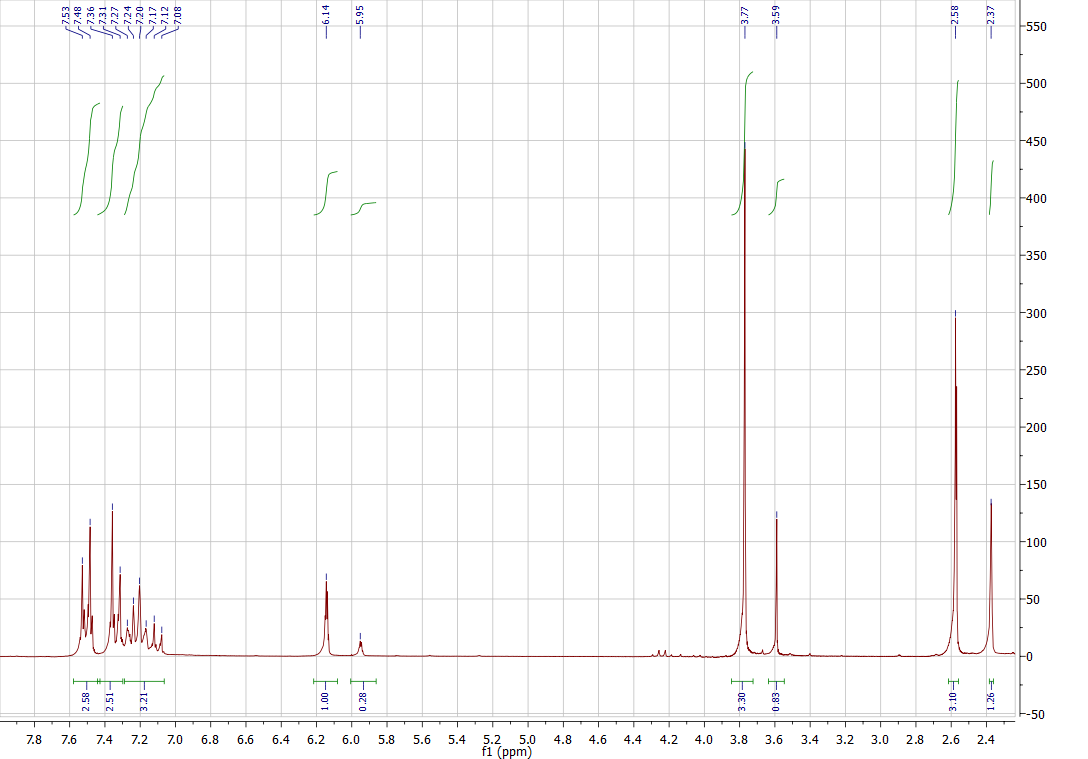


^1^H NMR of **17b**


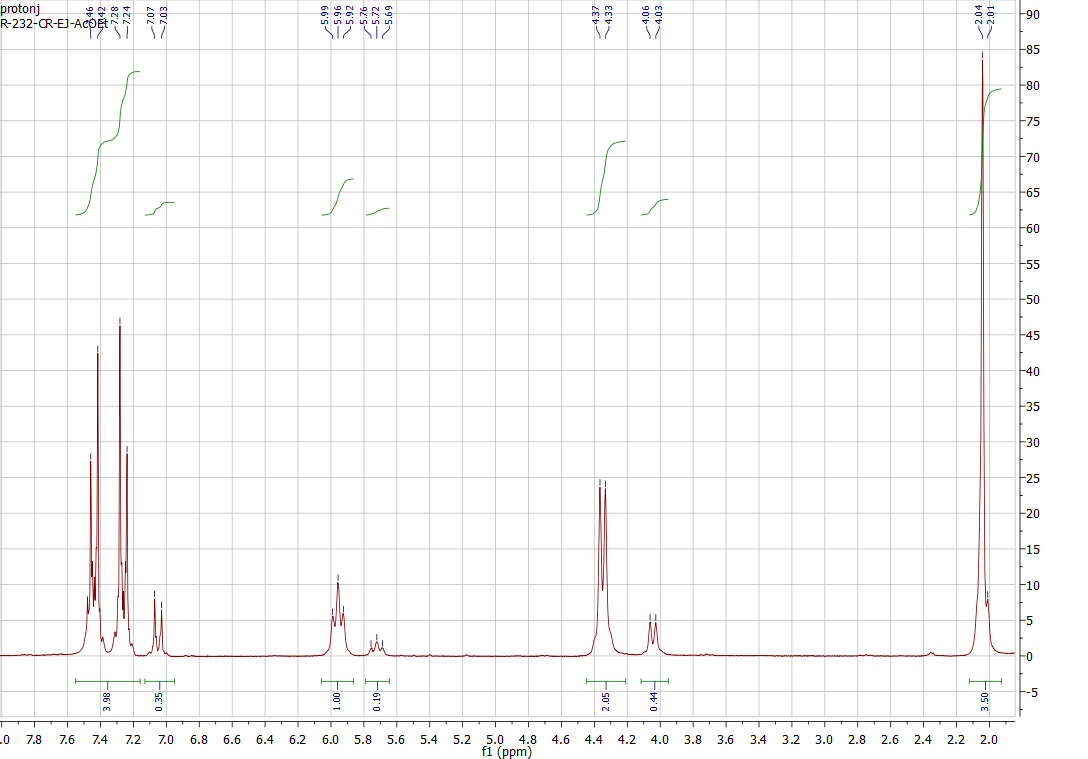


^1^H NMR of **17**


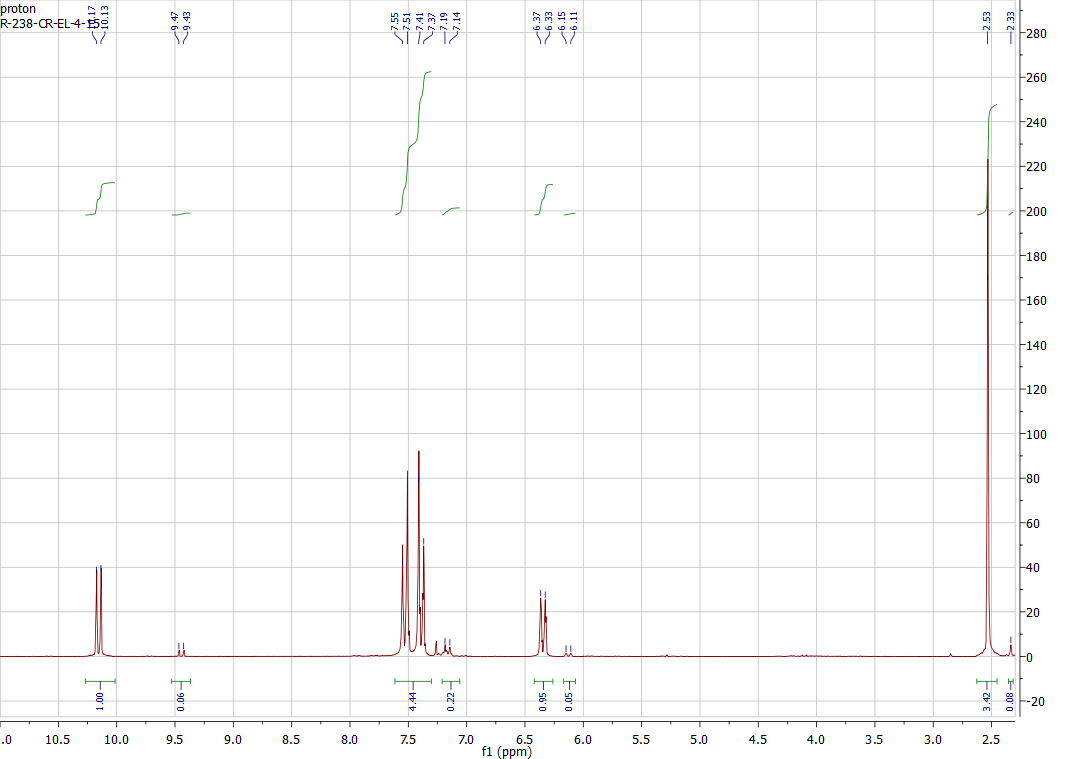


^1^H NMR of **18a**


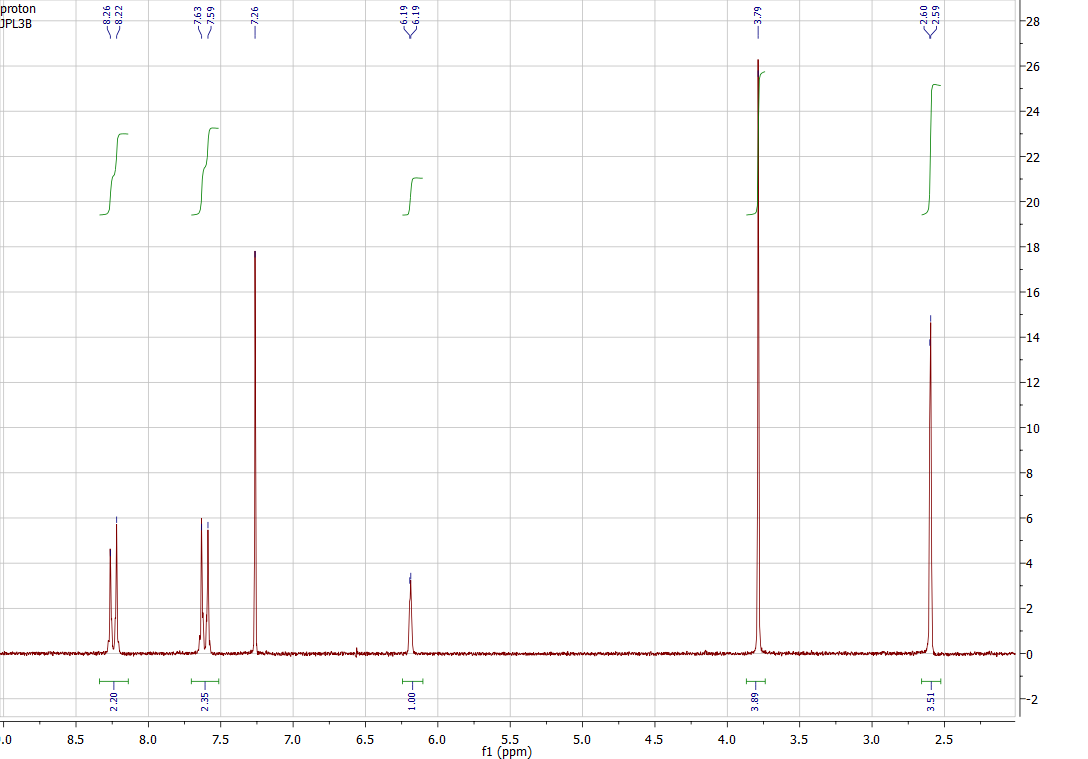


^1^H NMR of **18b**


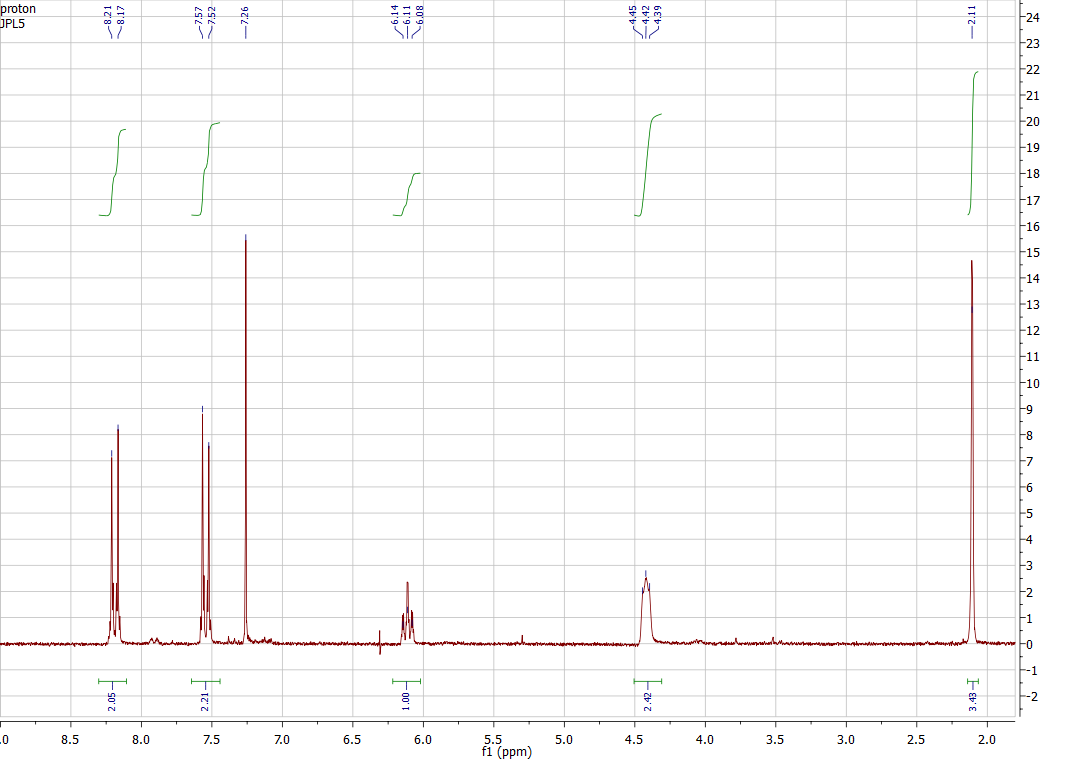


^1^H NMR of **18**


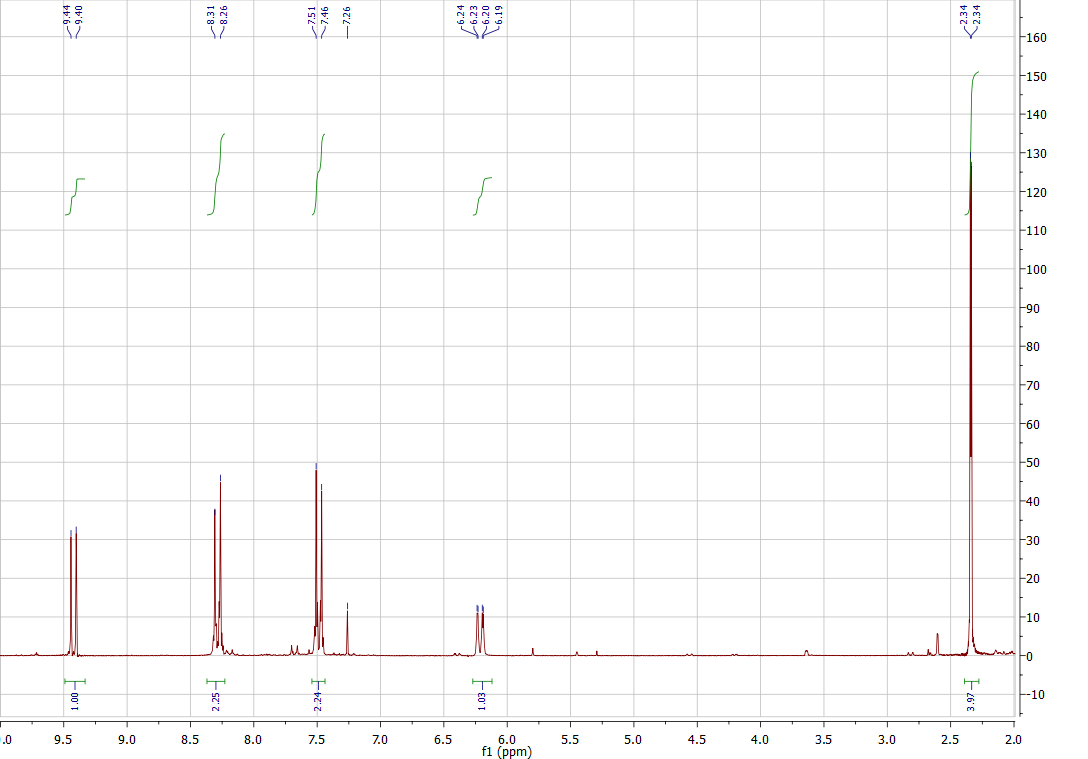


^1^H NMR of **19a**


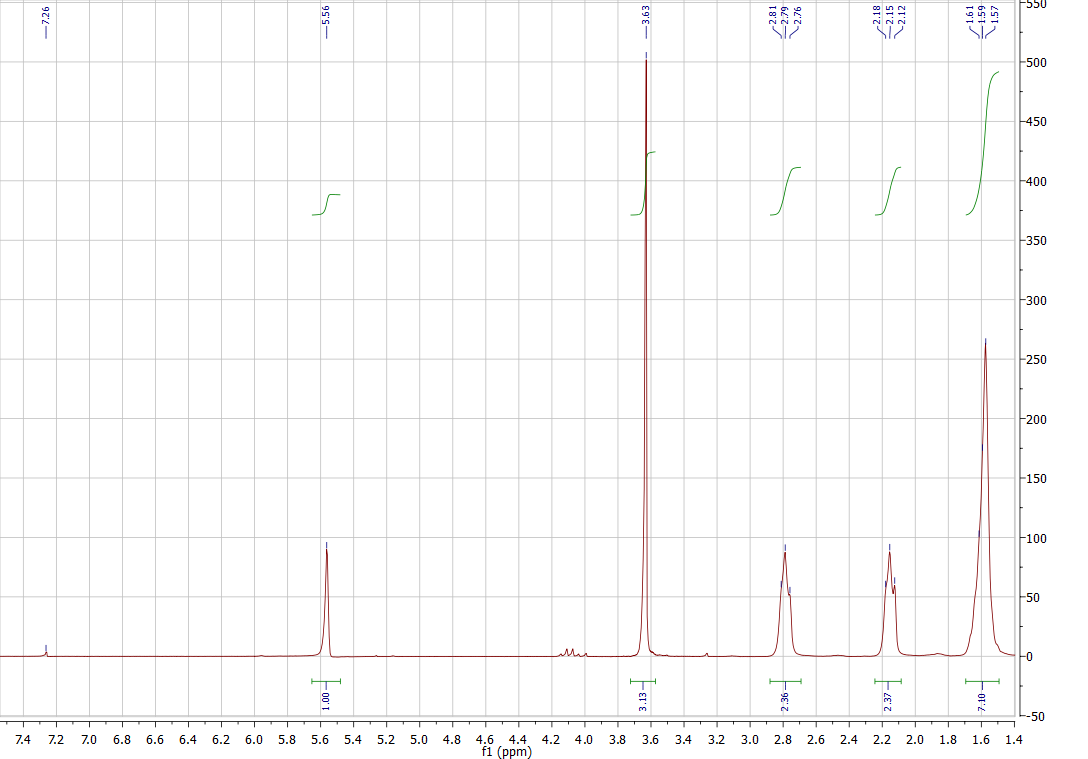


^1^H NMR of **19b**


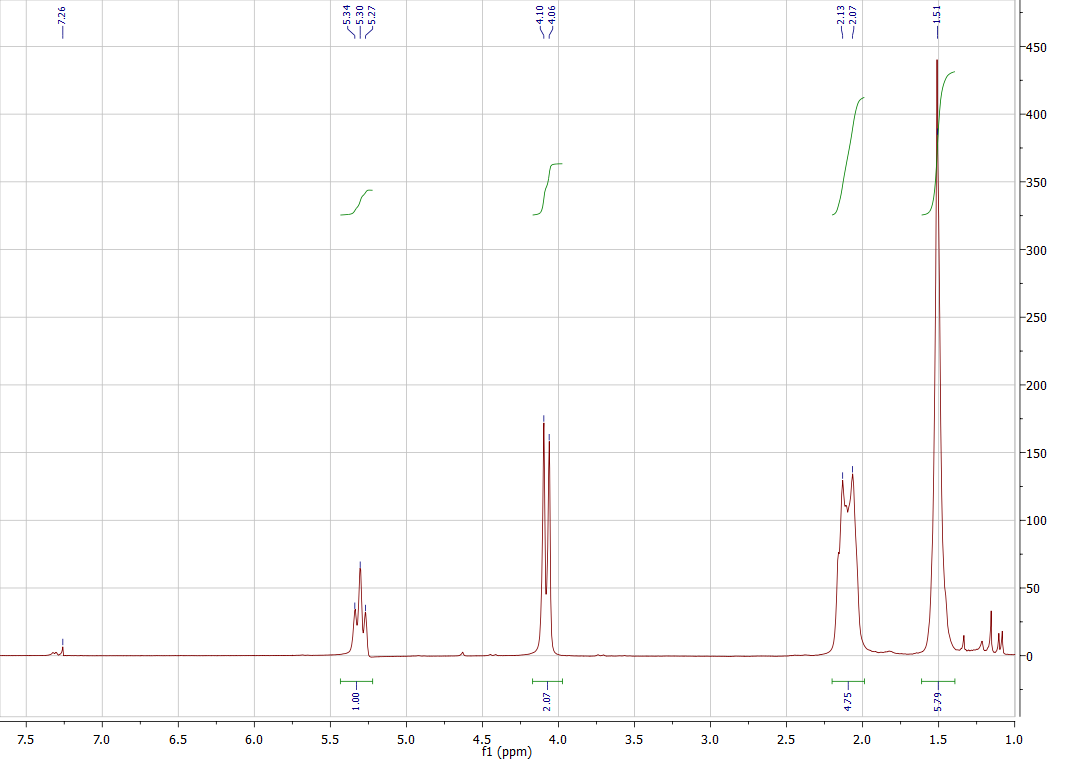


^1^H NMR of **19**


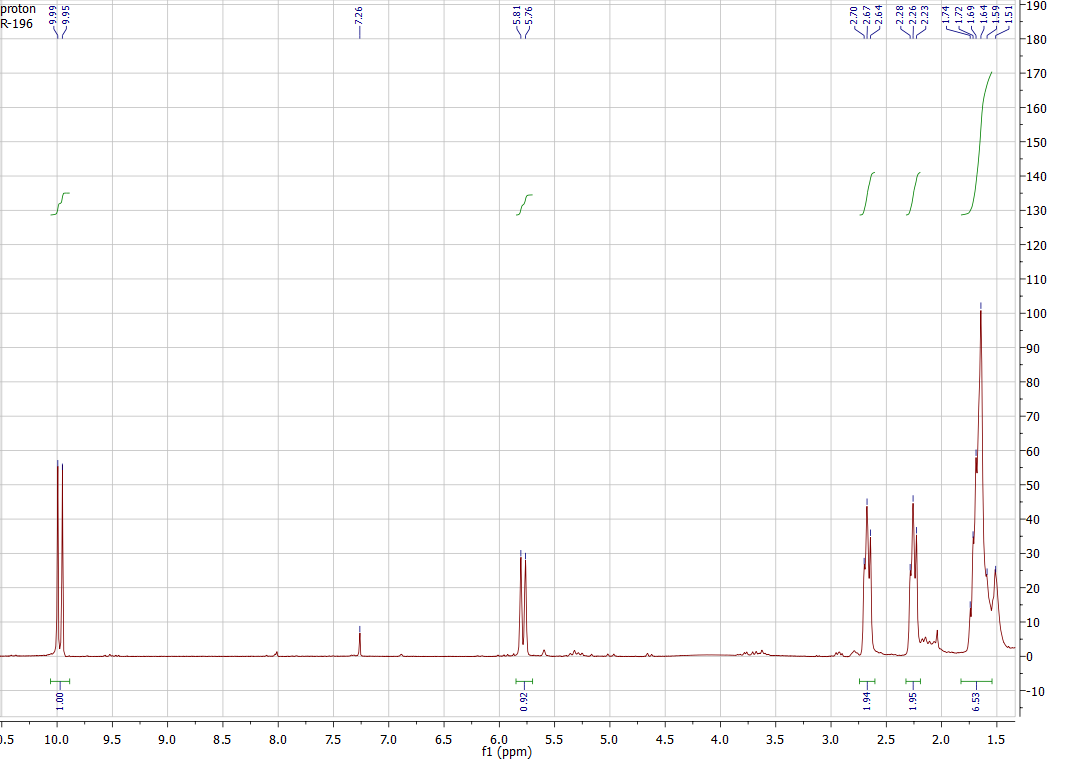


^1^H NMR and ^13^C NMR of **20a**


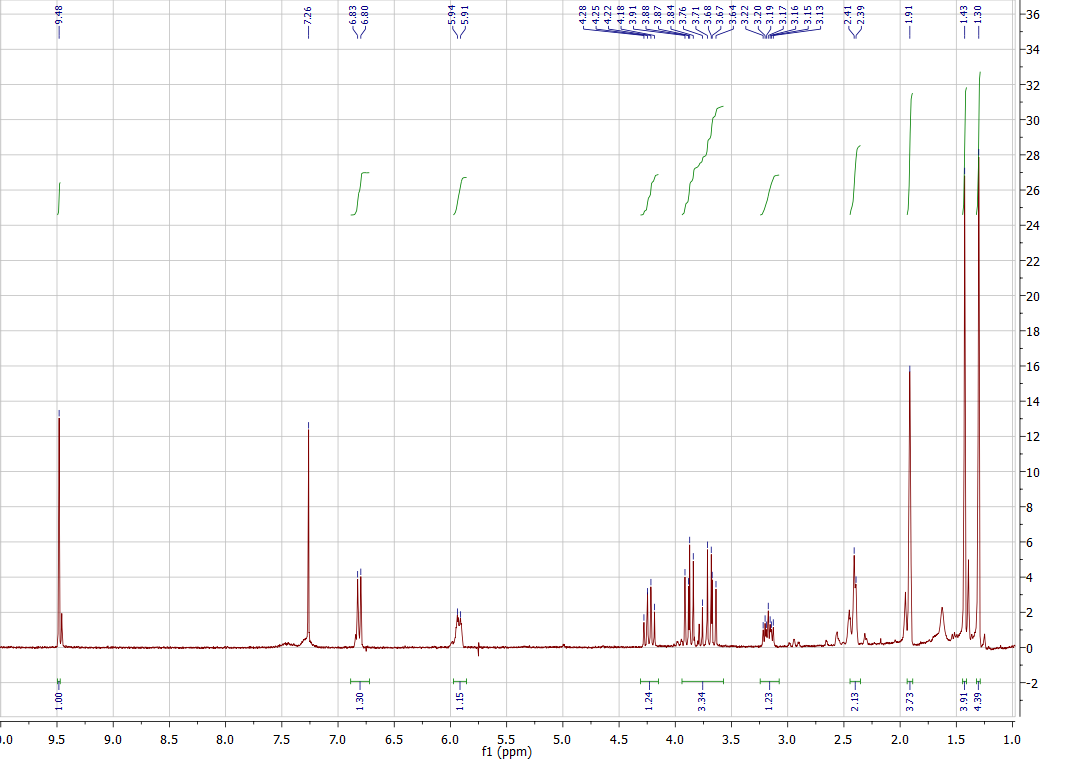


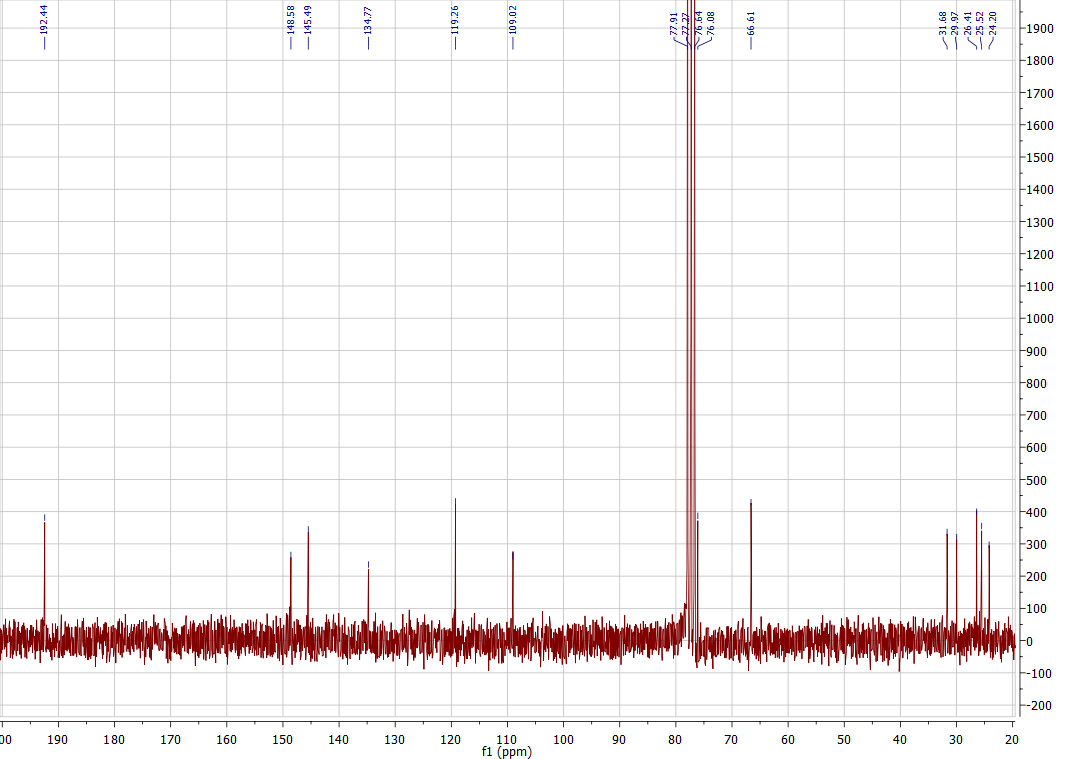


IR of **20a**

**
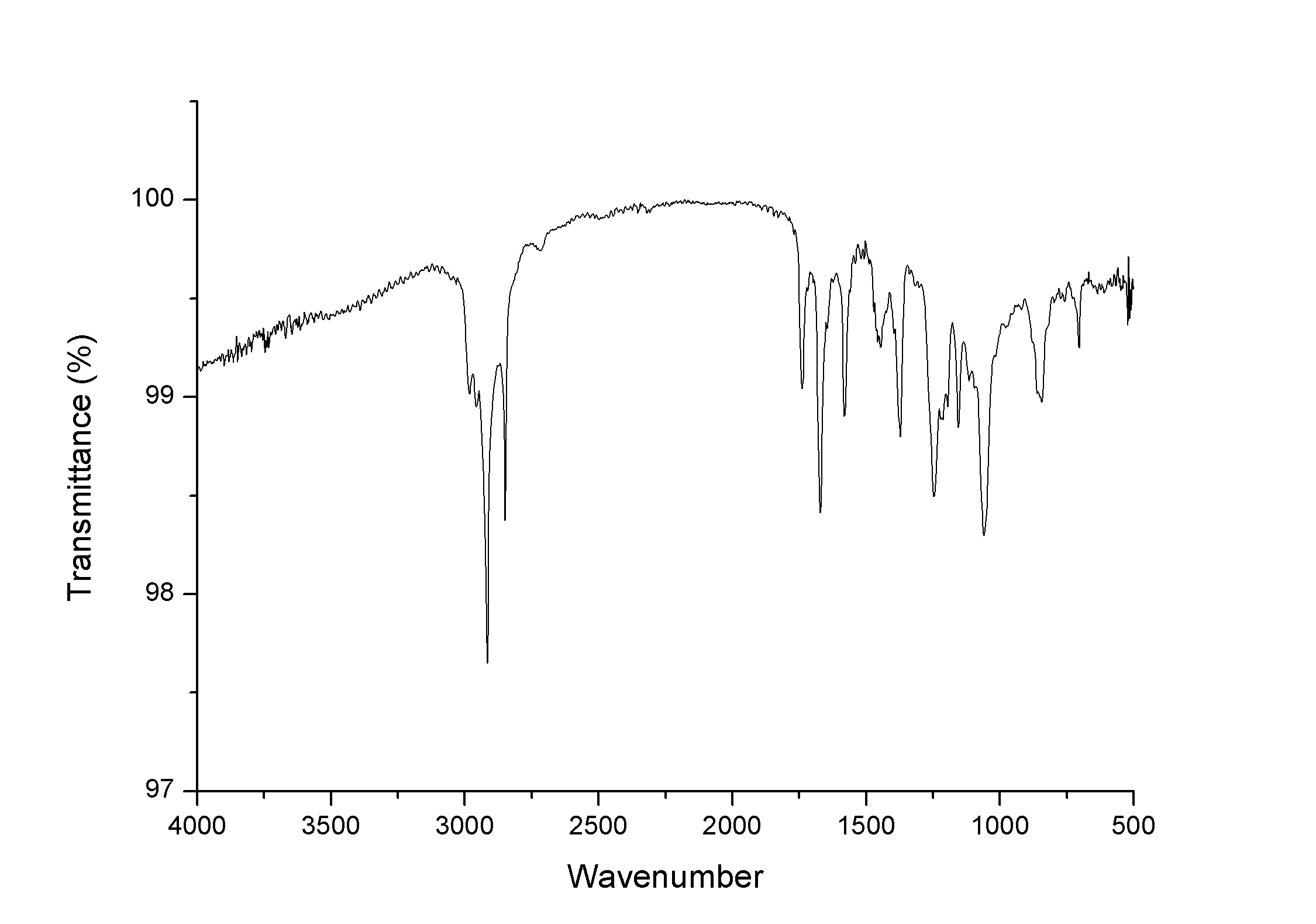
**

^1^H NMR and ^13^C NMR of **20b**


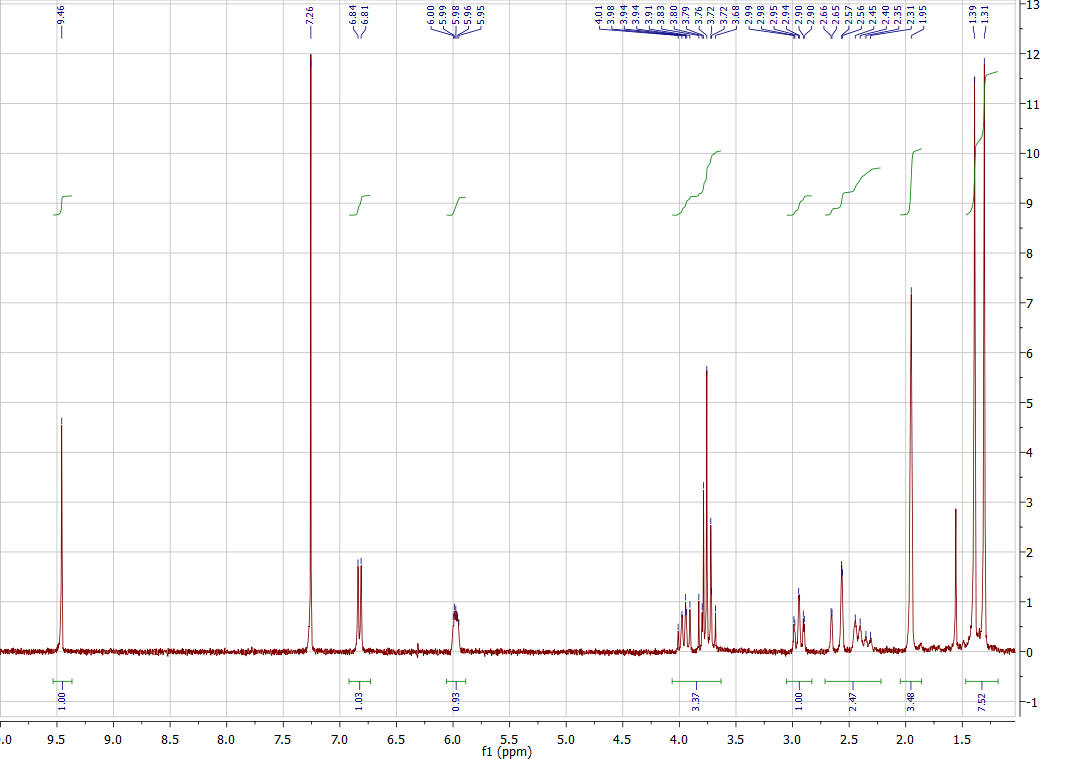


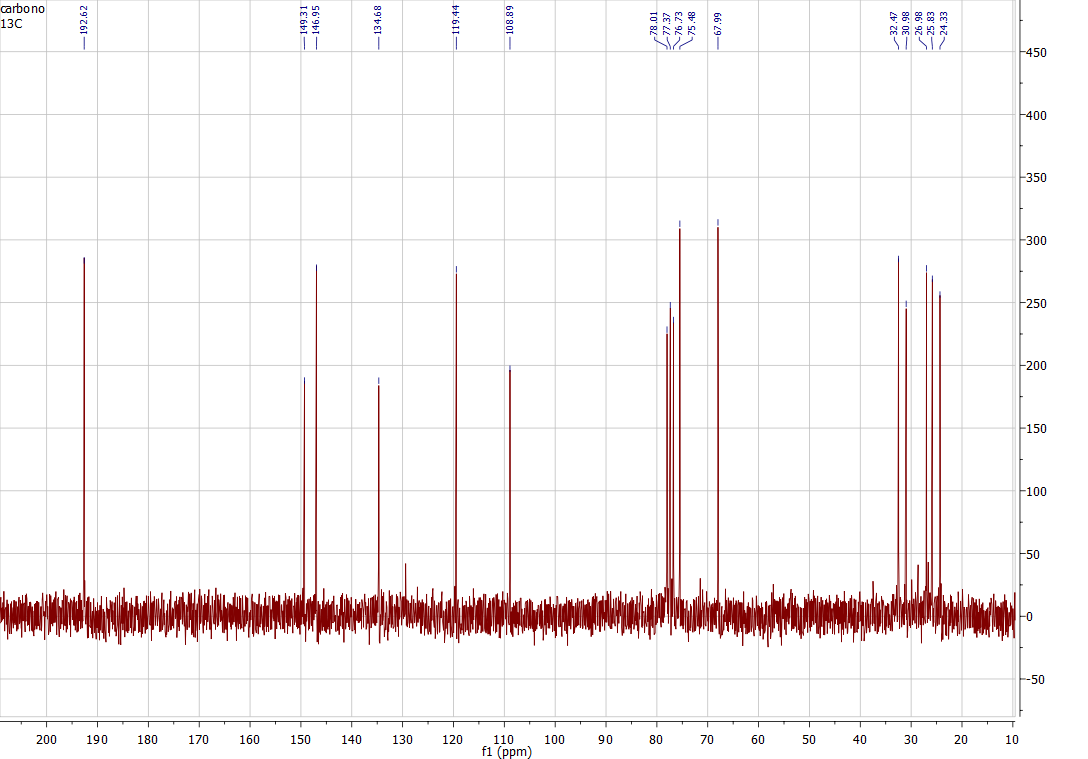


IR of **20b**

**
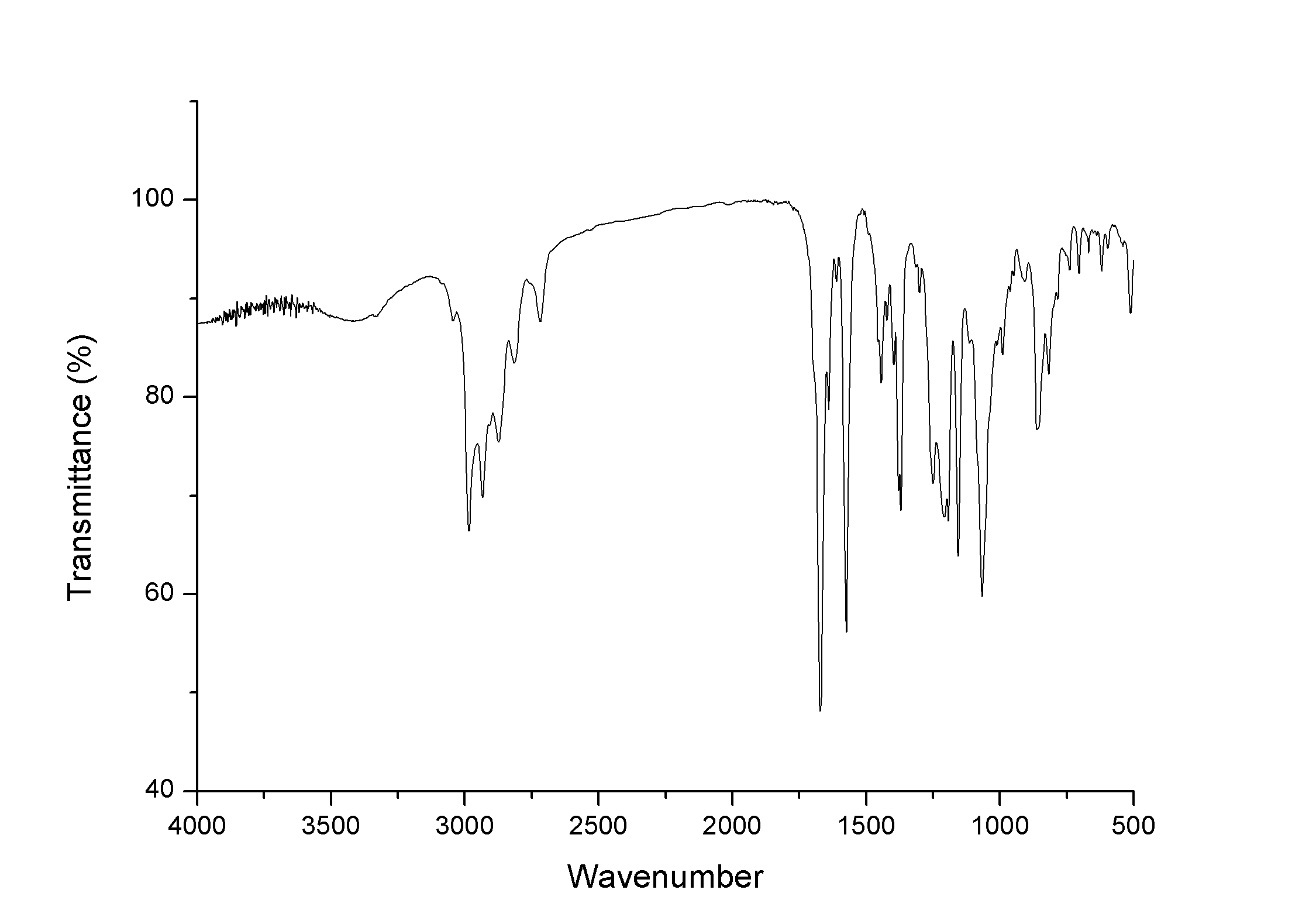
**

^1^H NMR and ^13^C NMR of **21a**


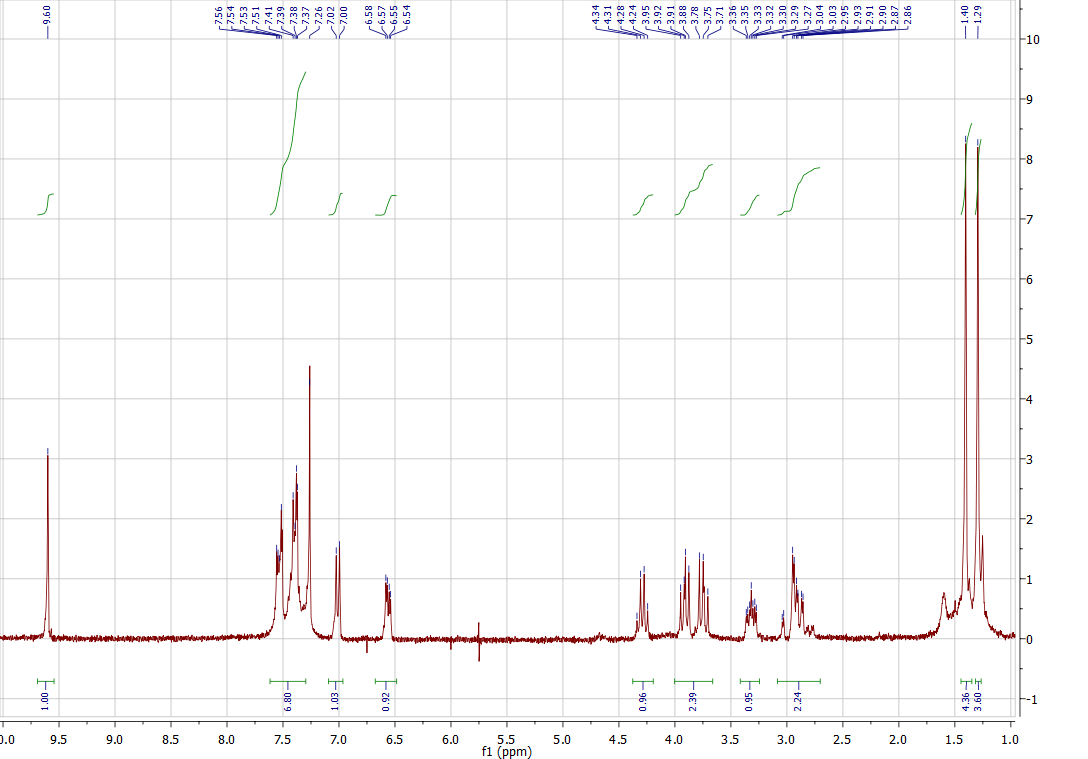


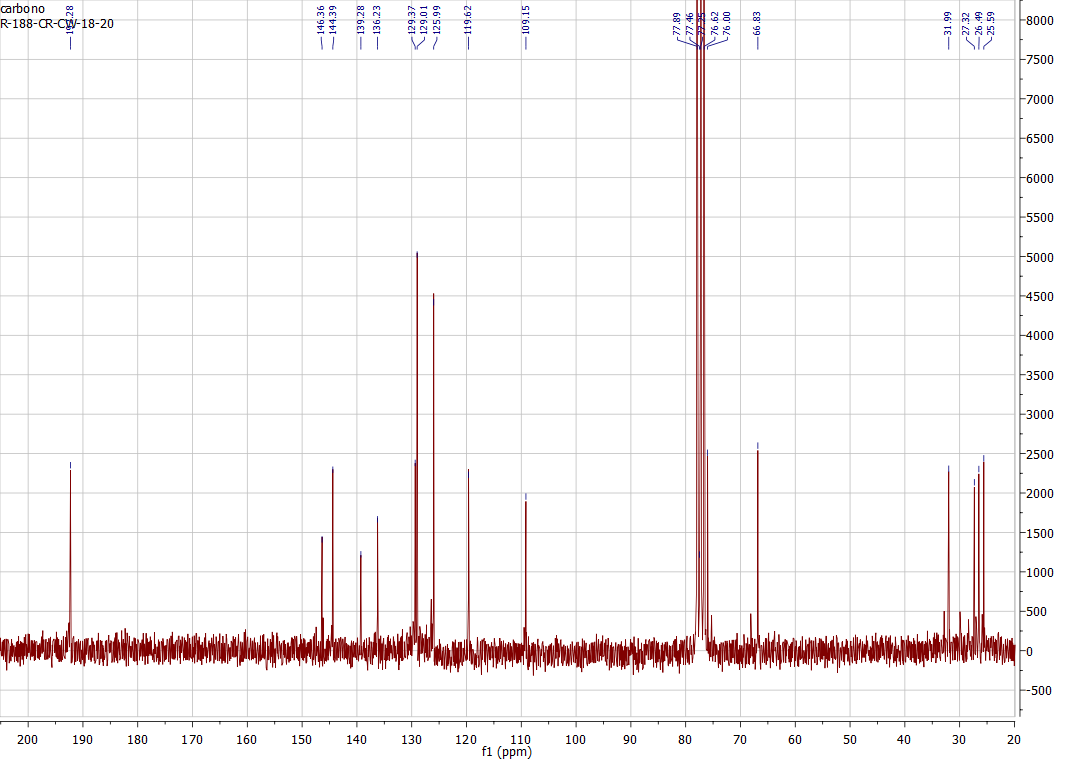


IR of **21a**

**
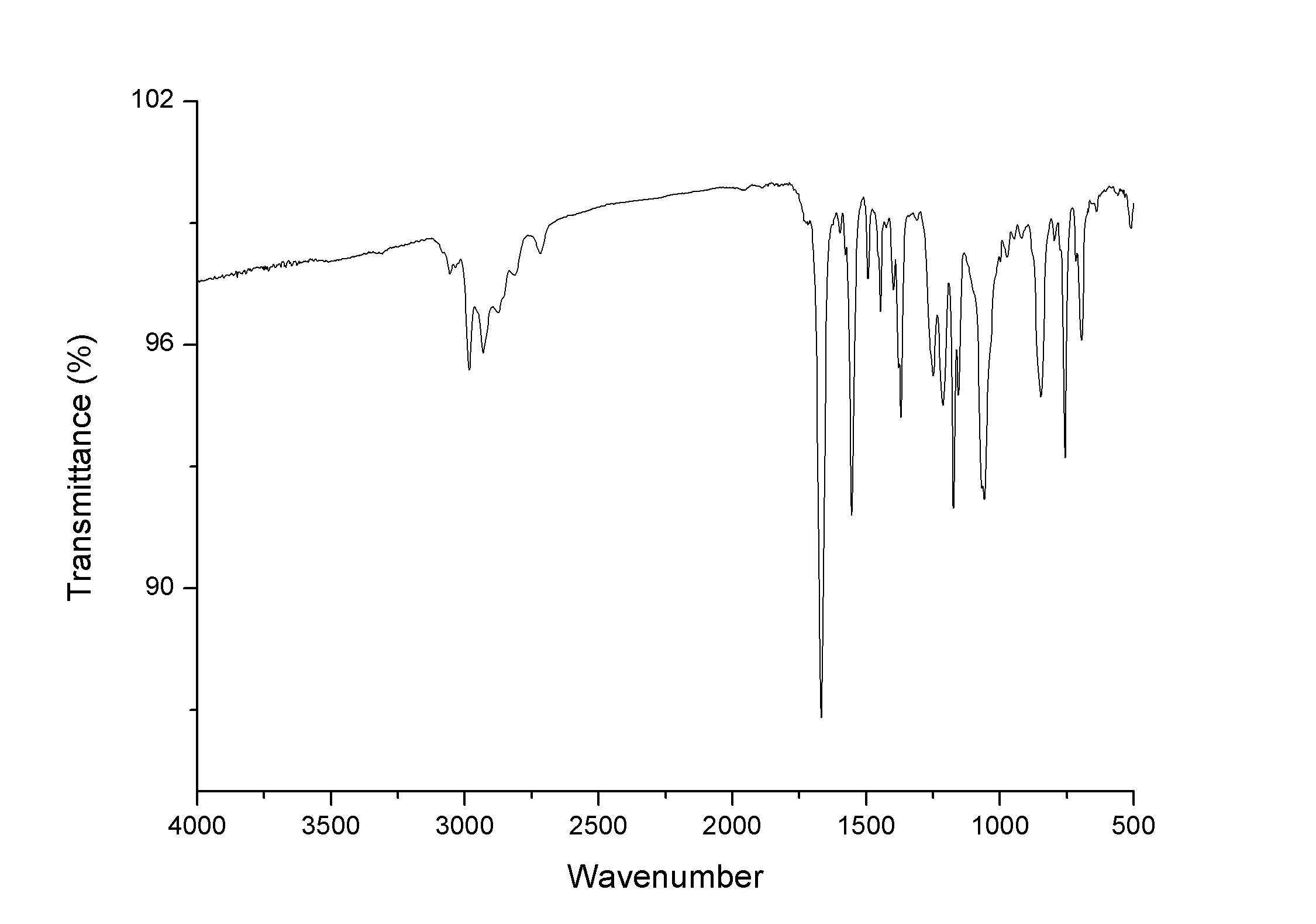
**

^1^H NMR and ^13^C NMR of **21b**


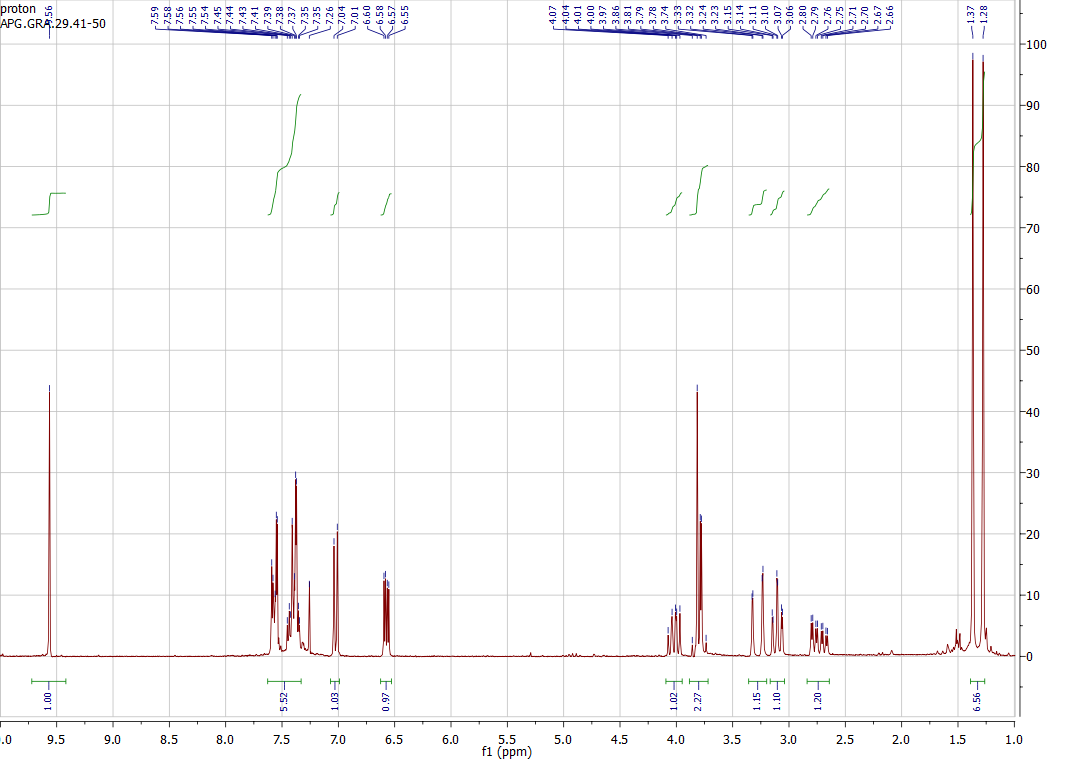


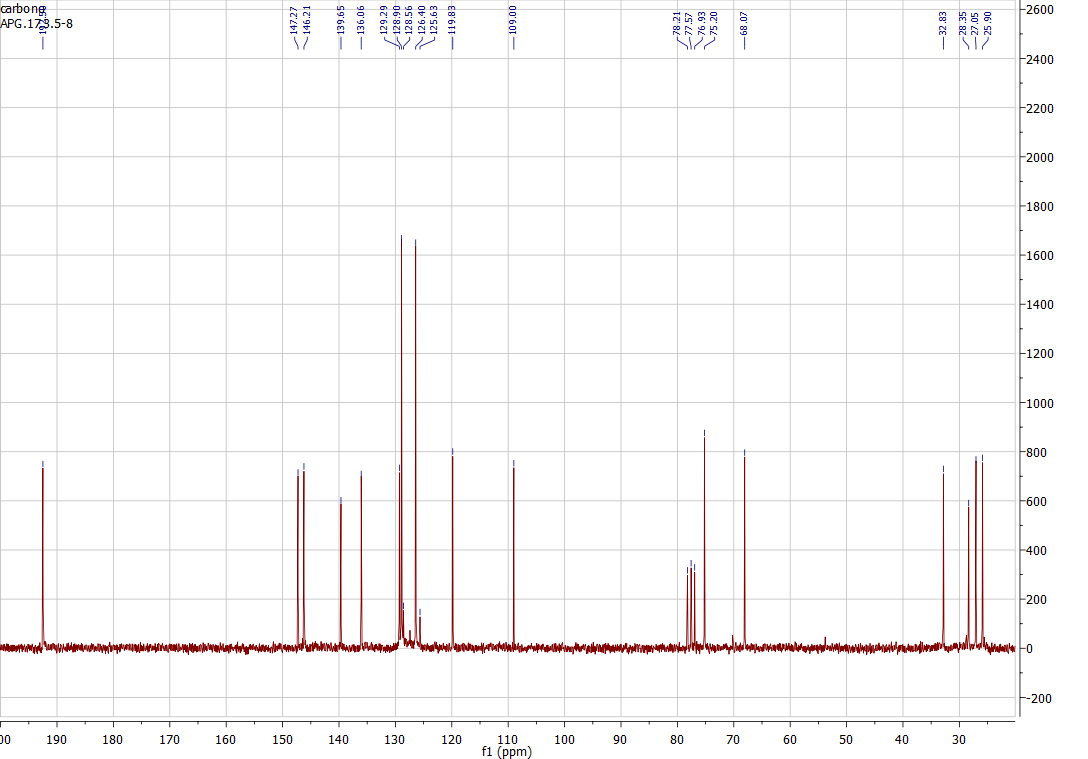


IR of **21b**

**
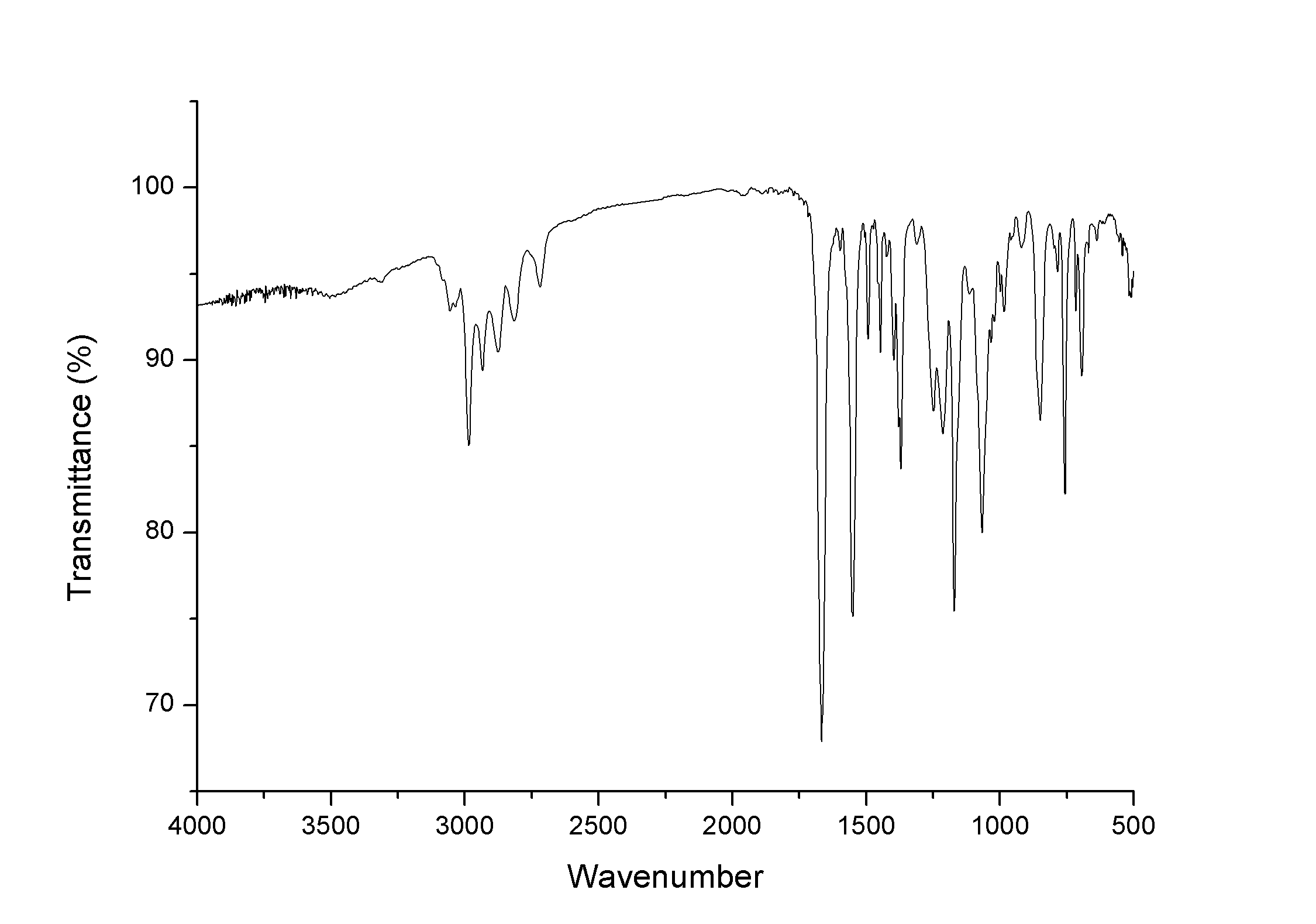
**

^1^H NMR and ^13^C NMR of **22a**


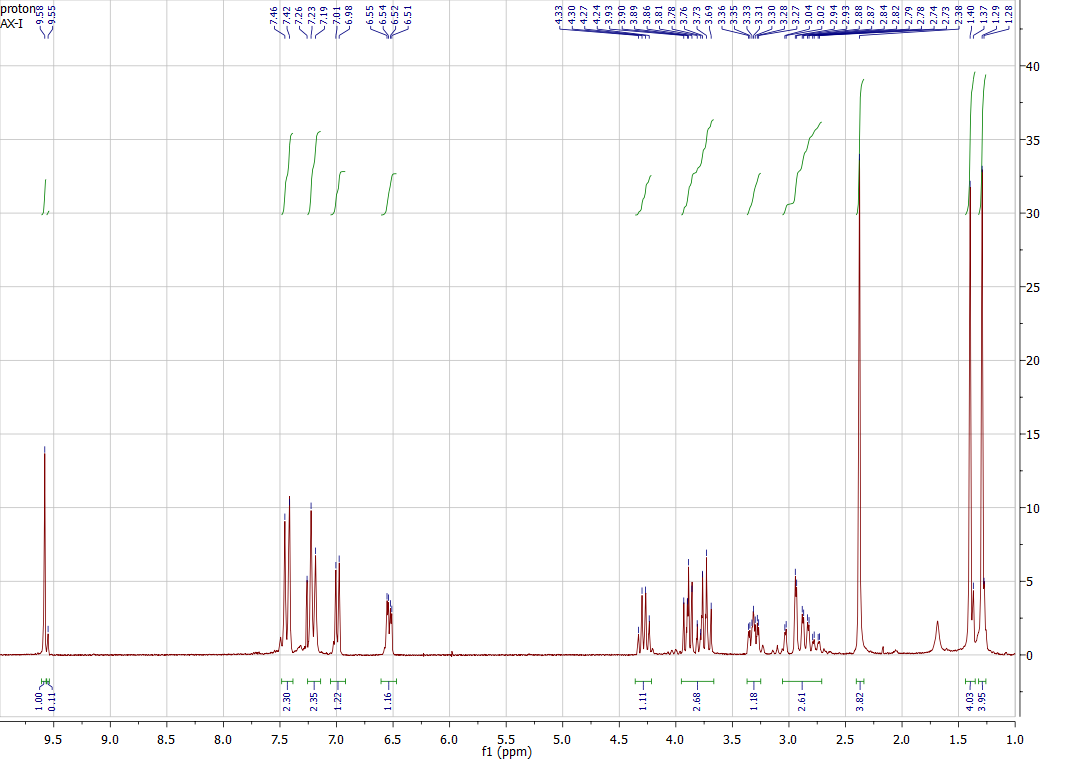


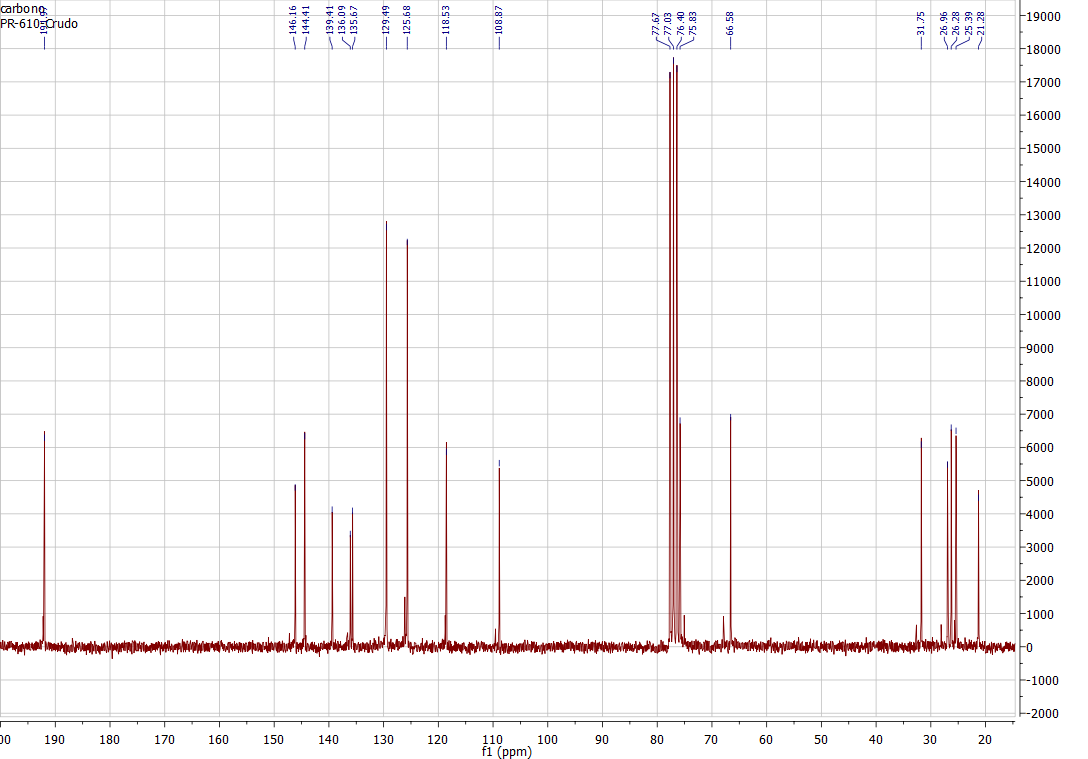


IR of **22a**

**
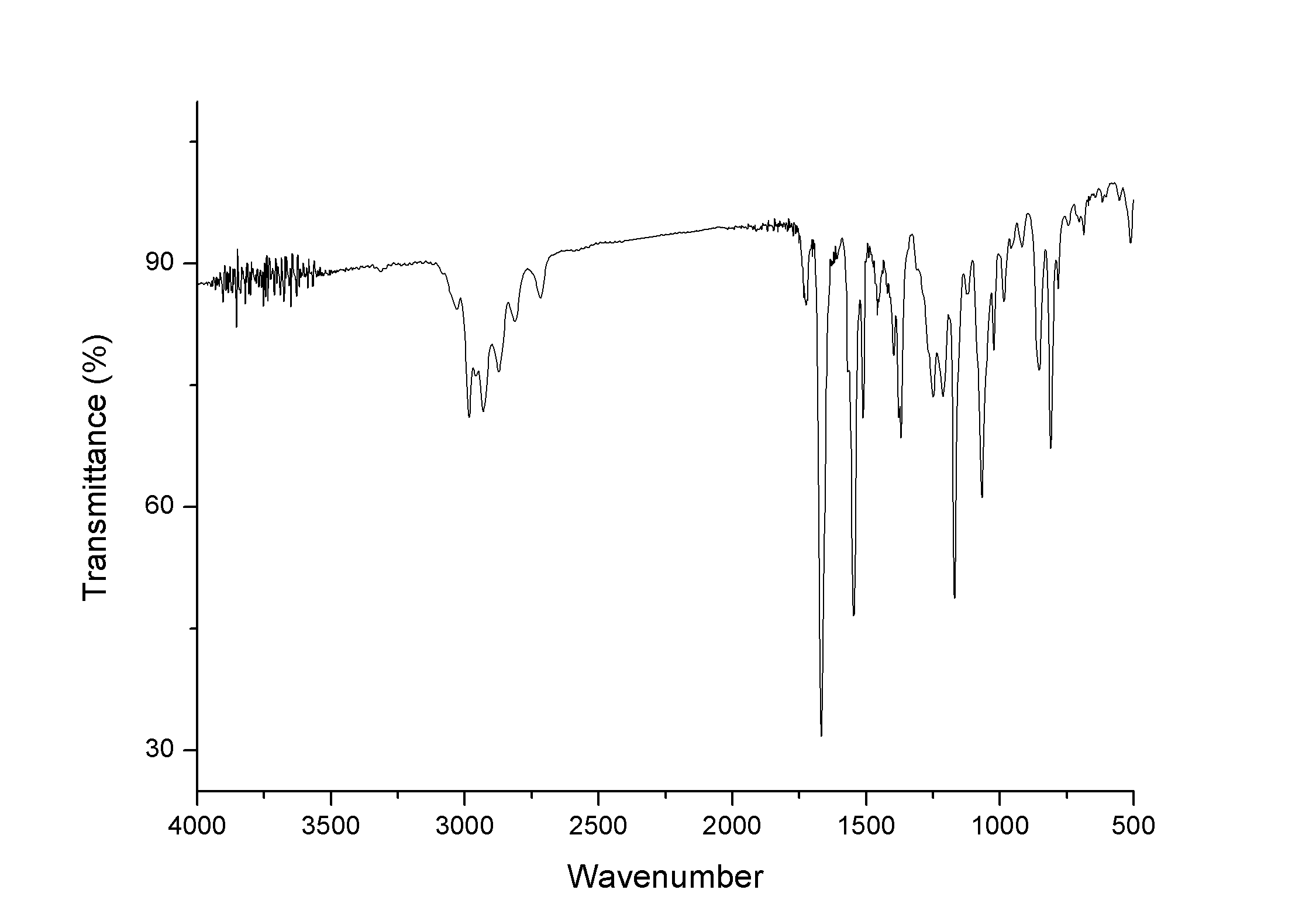
**

^1^H NMR and ^13^C NMR of **22b**


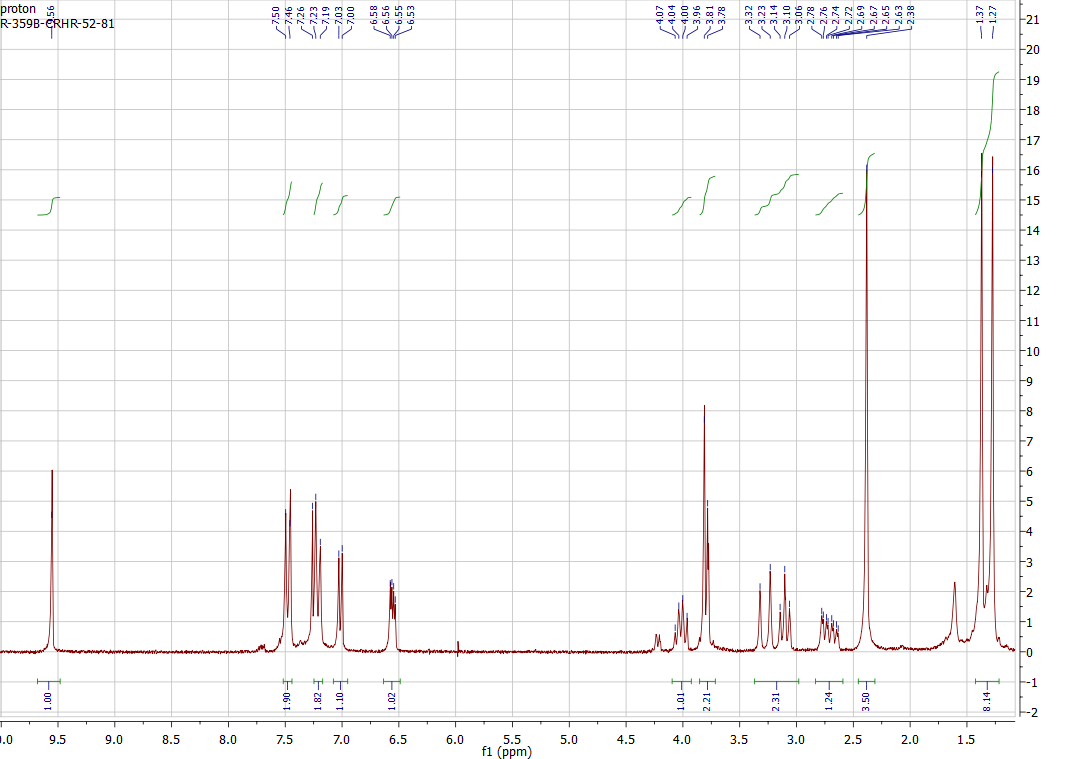


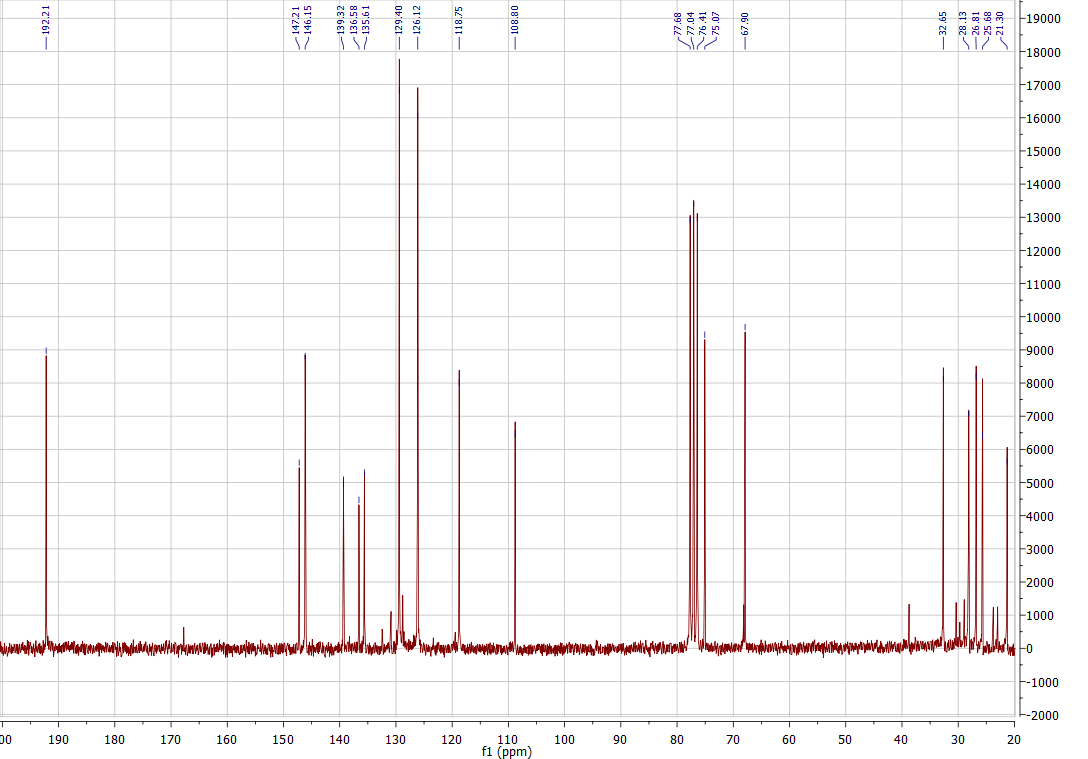


IR of **22b**

**
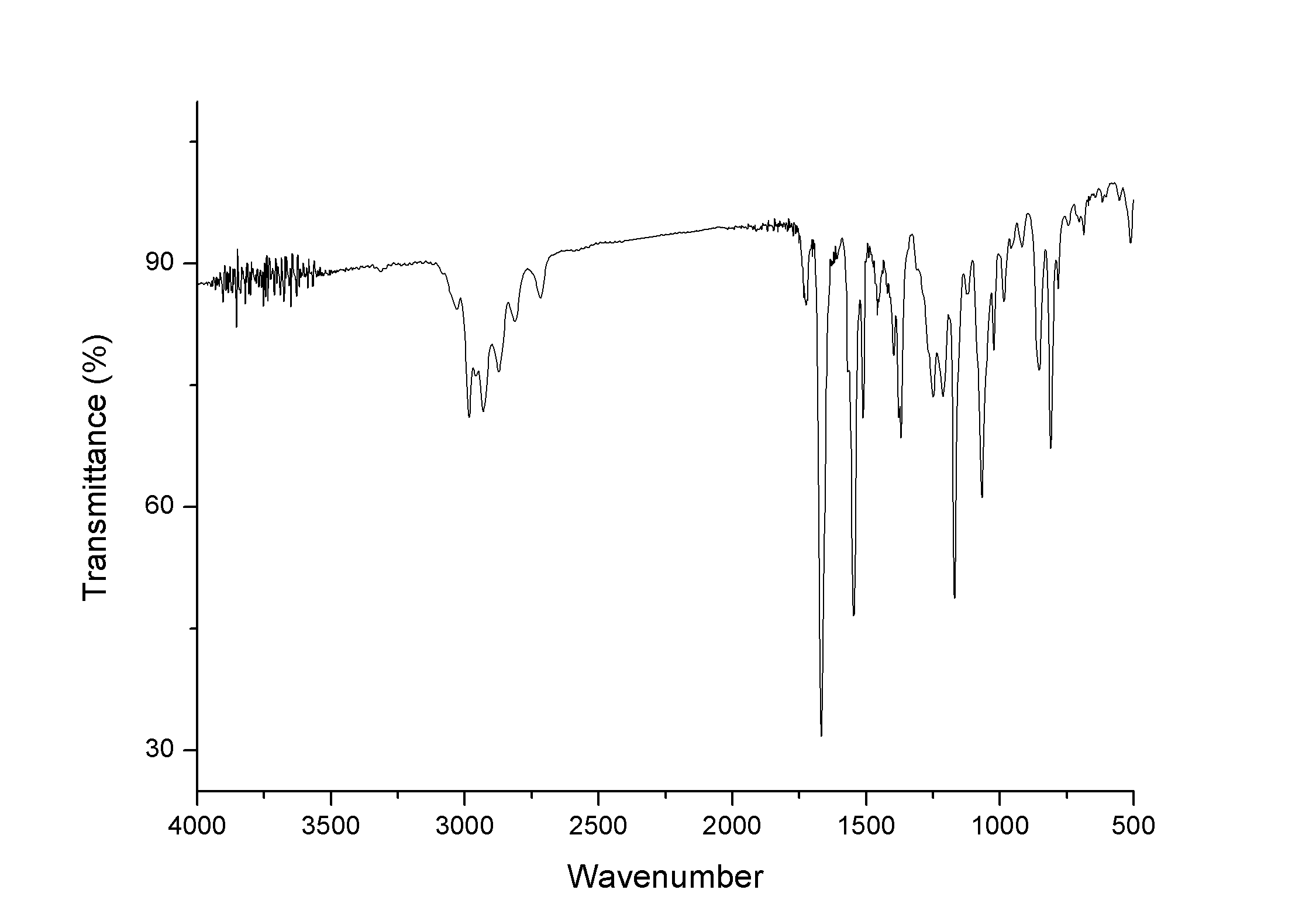
**

^1^H NMR and ^13^C NMR of **23a**


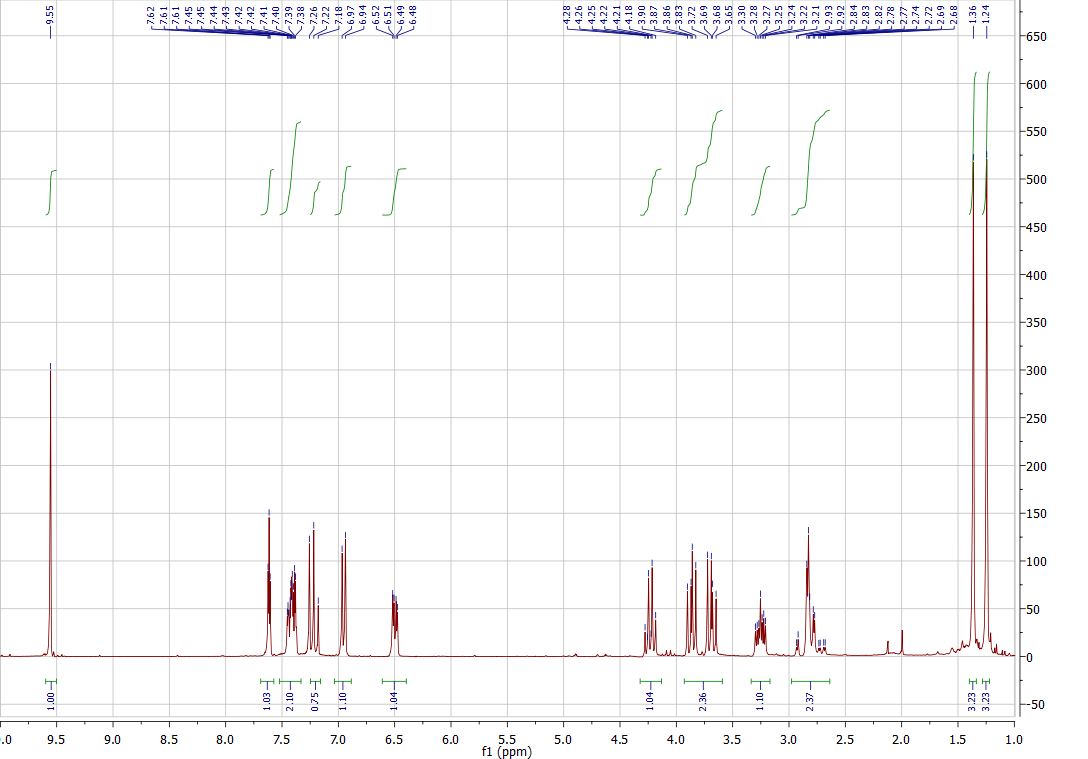


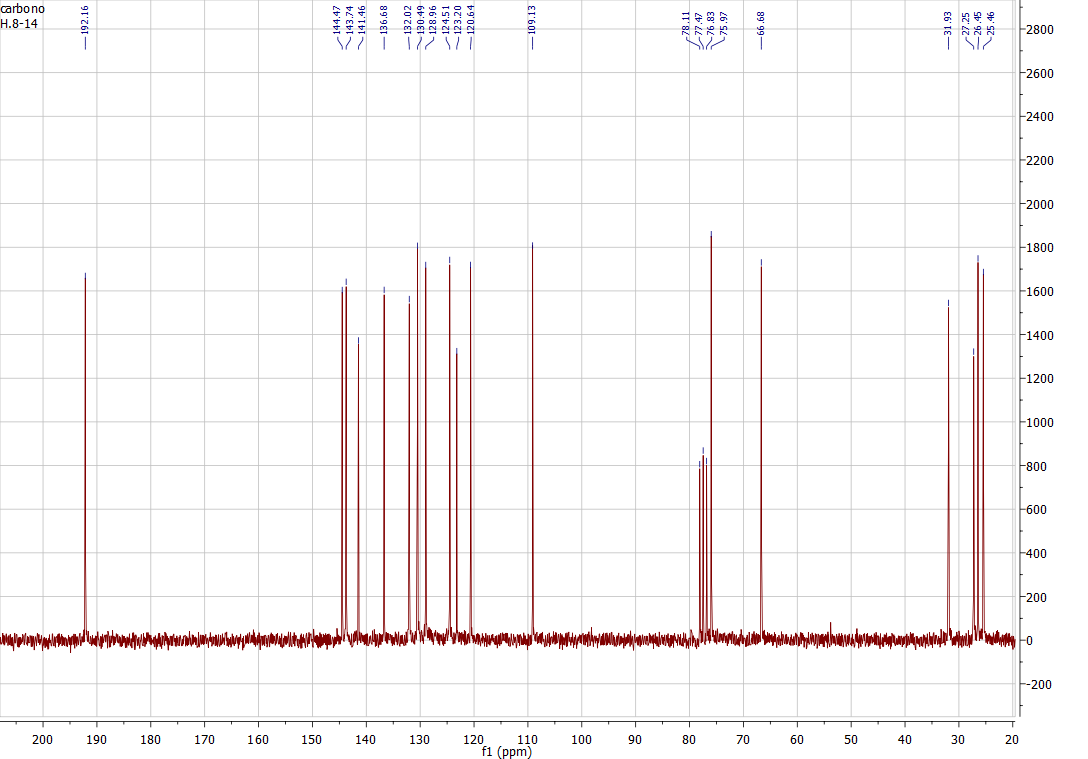


IR of **23a**

**
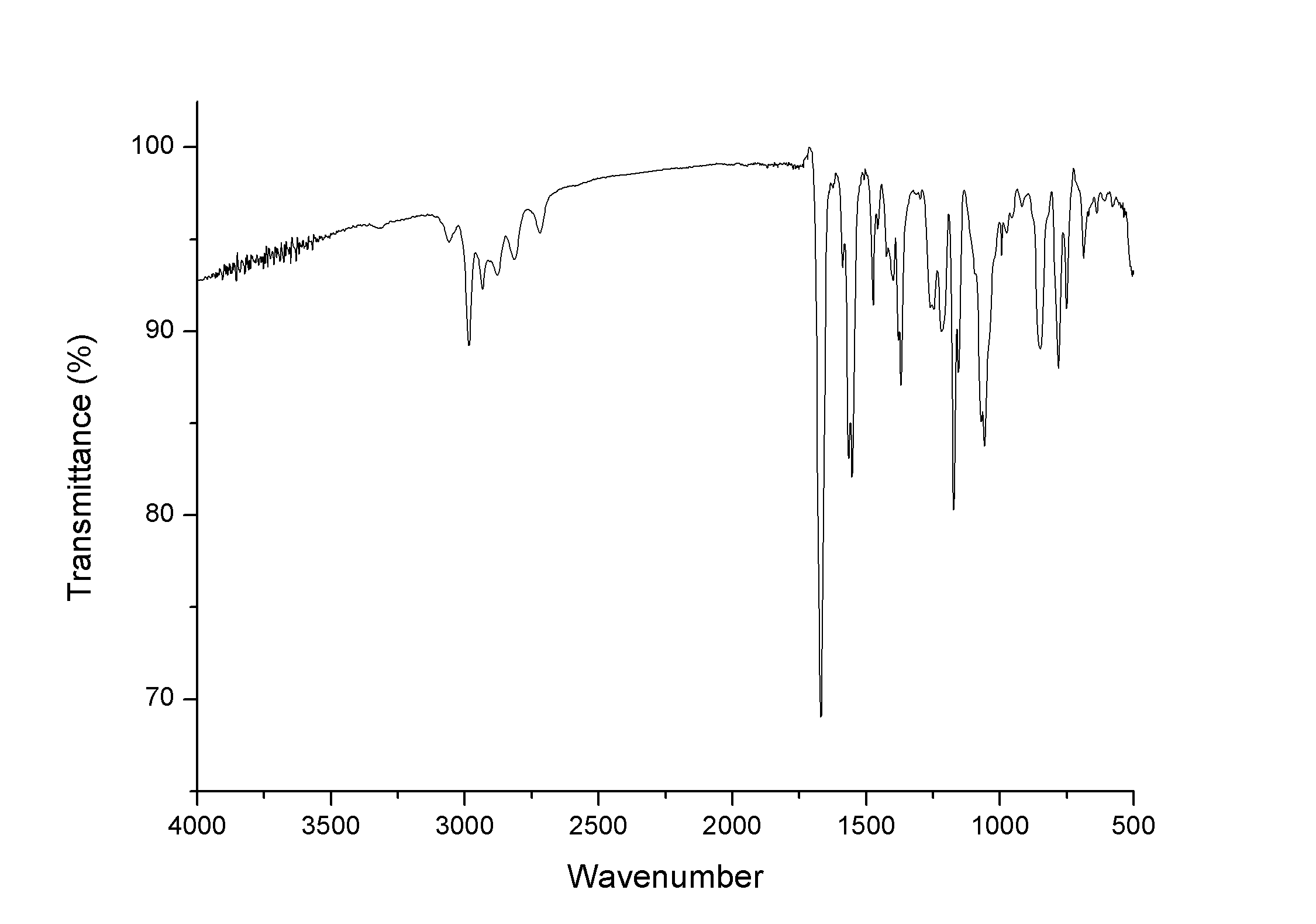
**

^1^H NMR and ^13^C of NMR **23b**


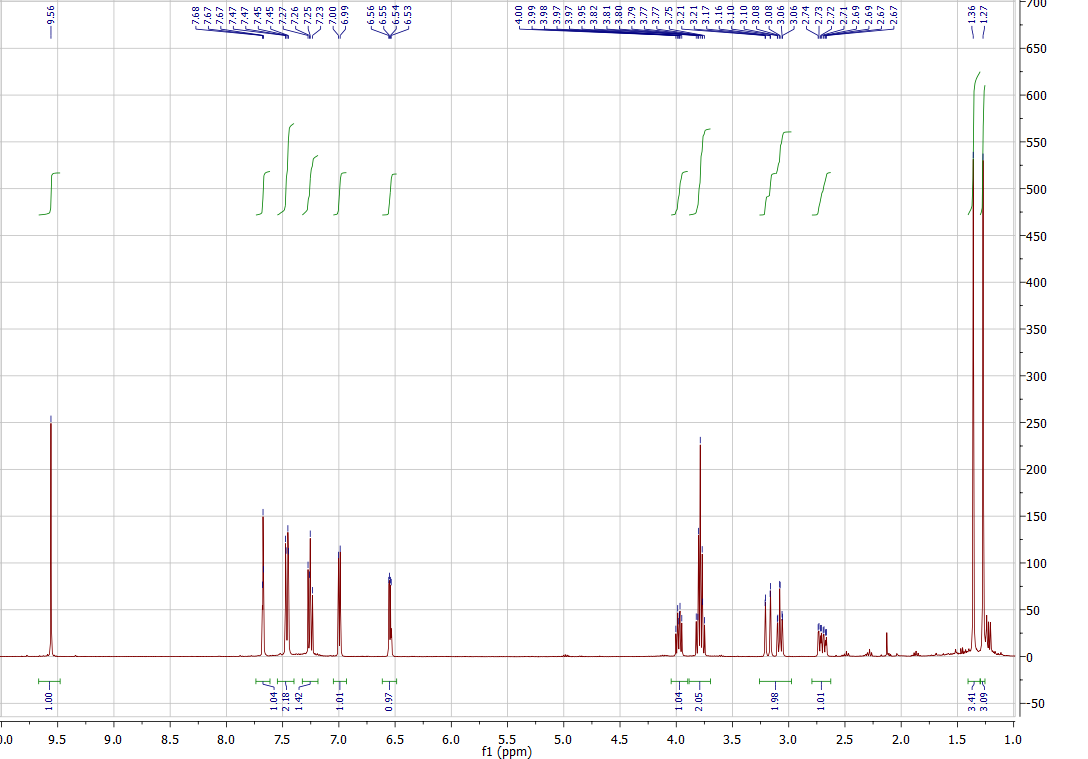


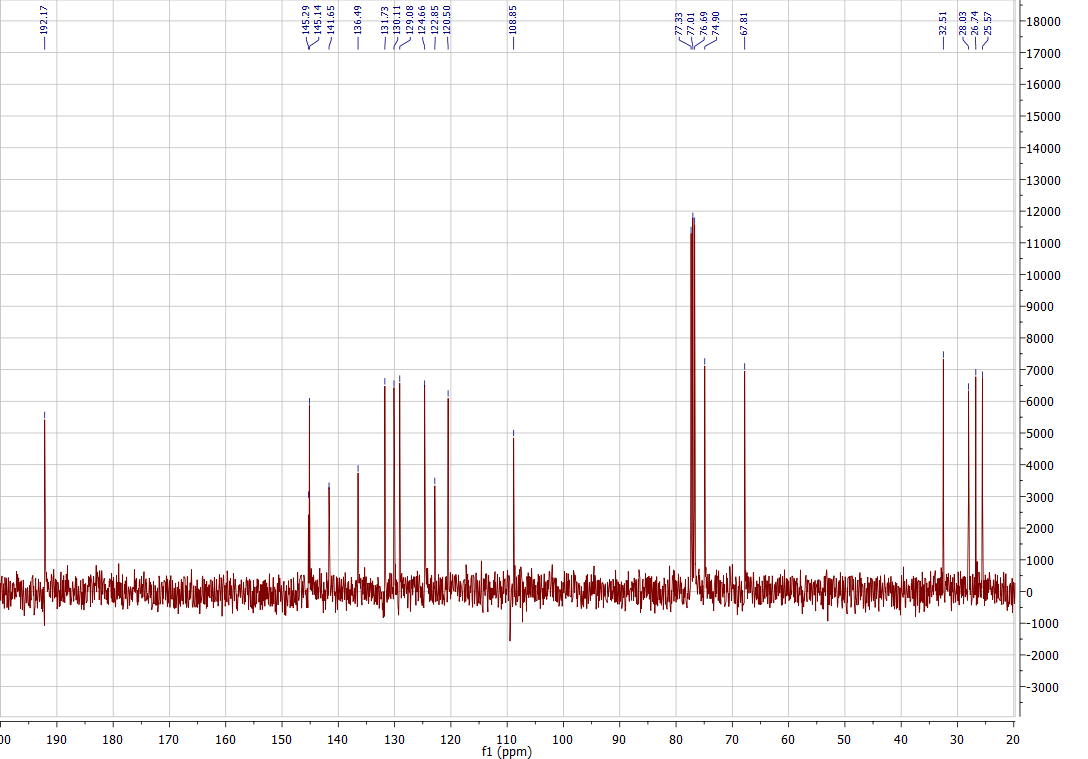


IR of **23b**

**
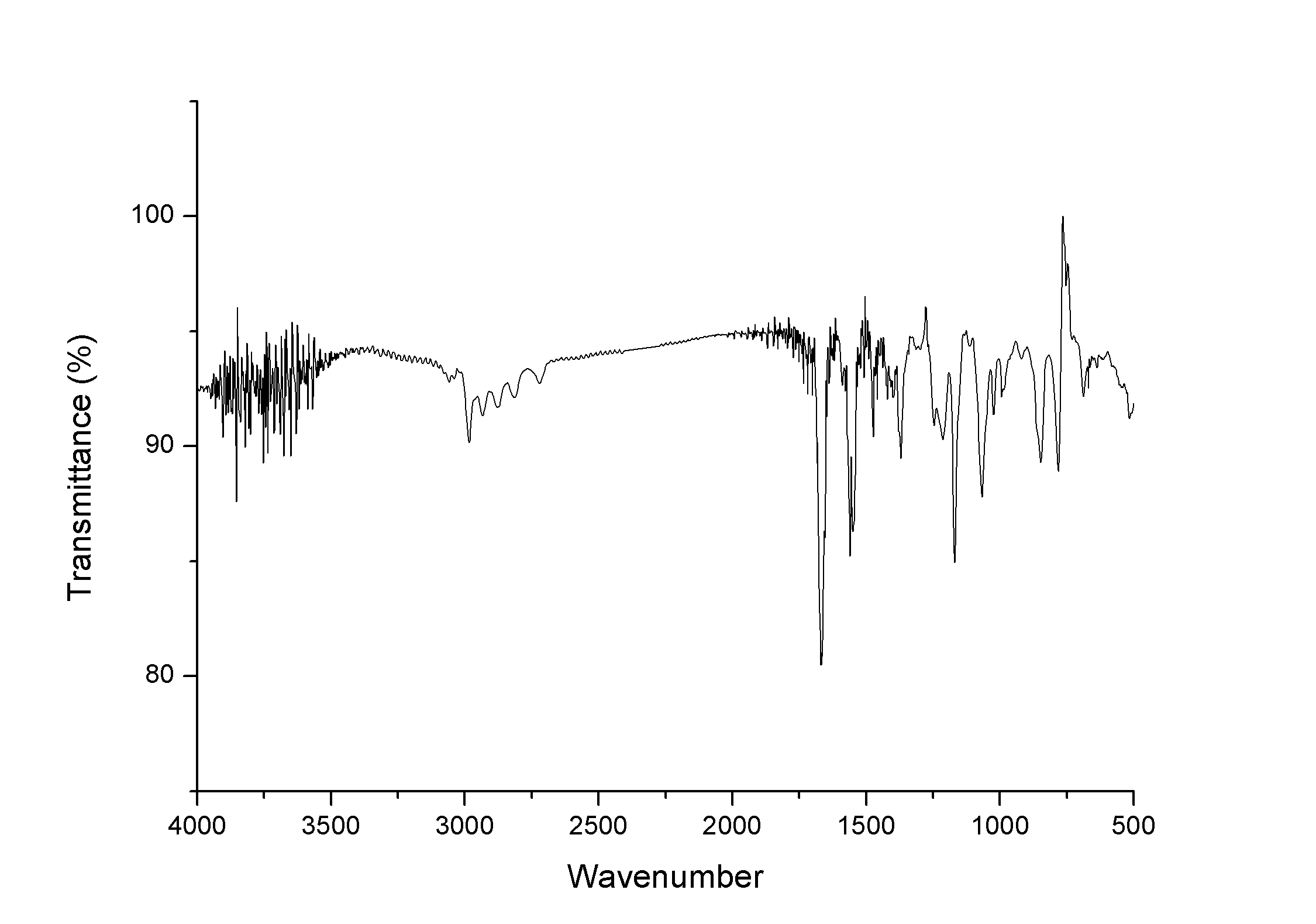
**

^1^H NMR and ^13^C of NMR **24a**


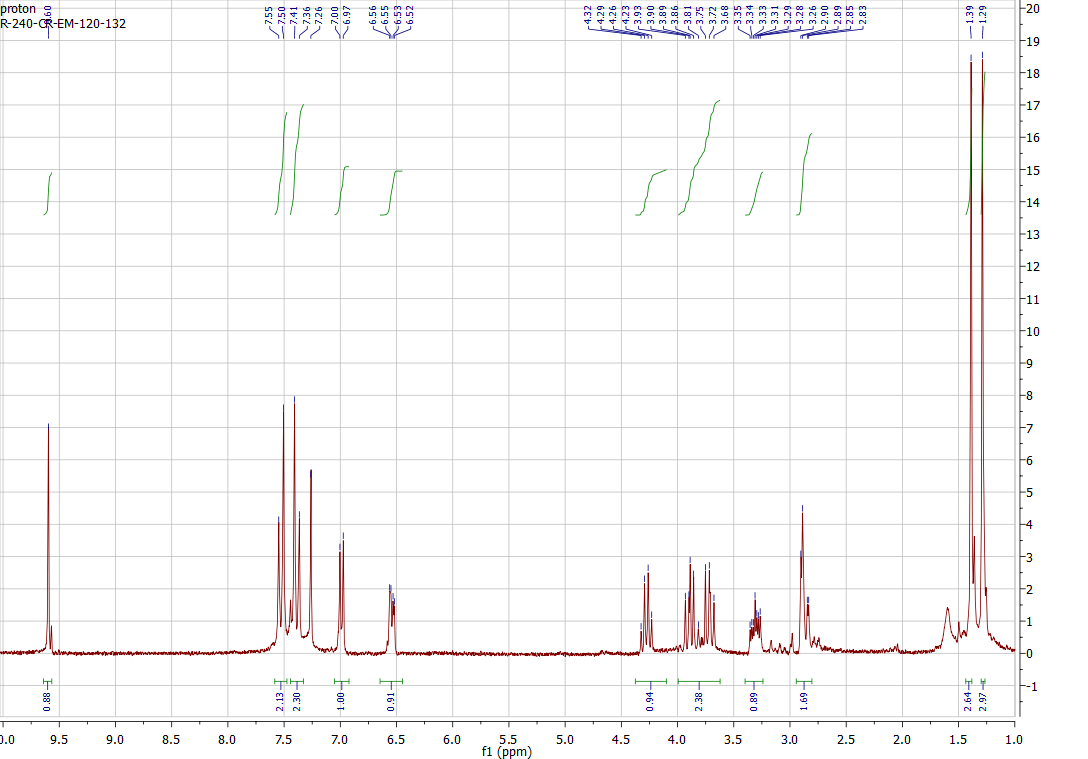


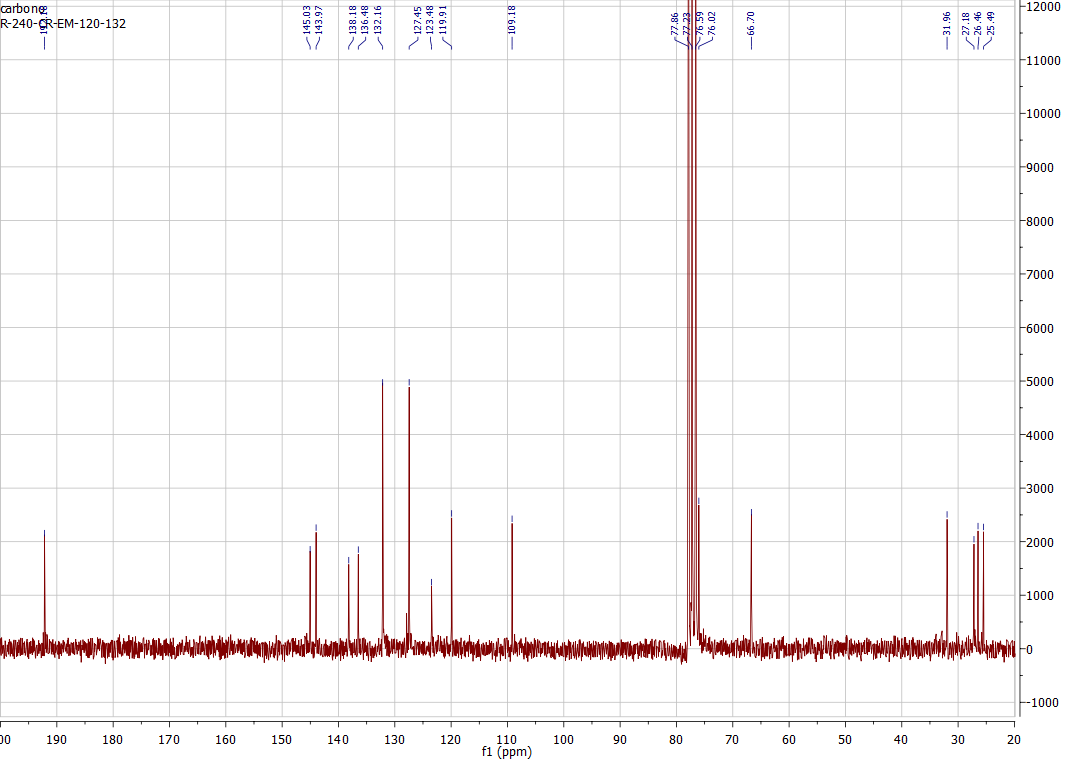


IR of **24a**

**
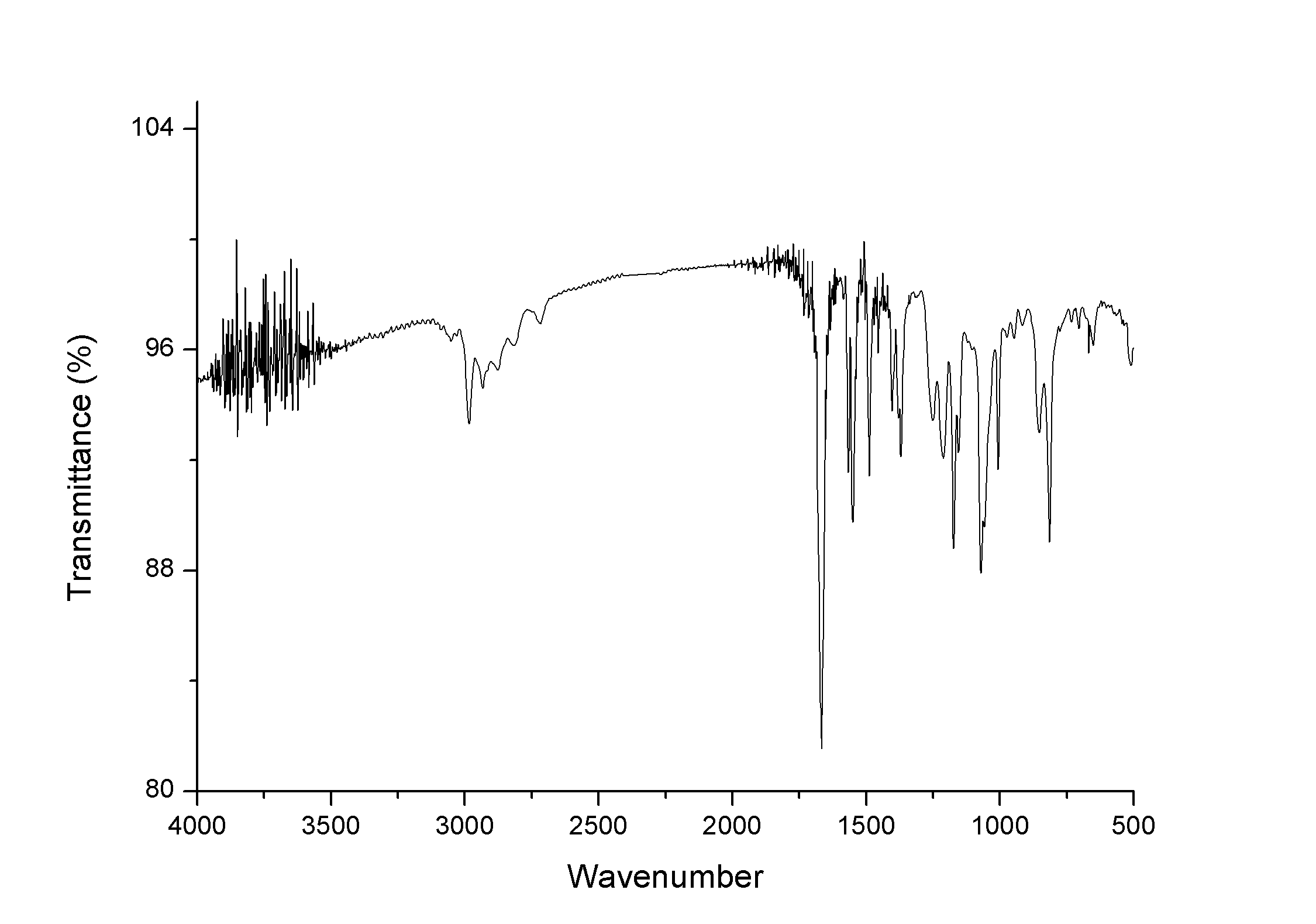
**

^1^H NMR and ^13^C NMR of **24b**

**
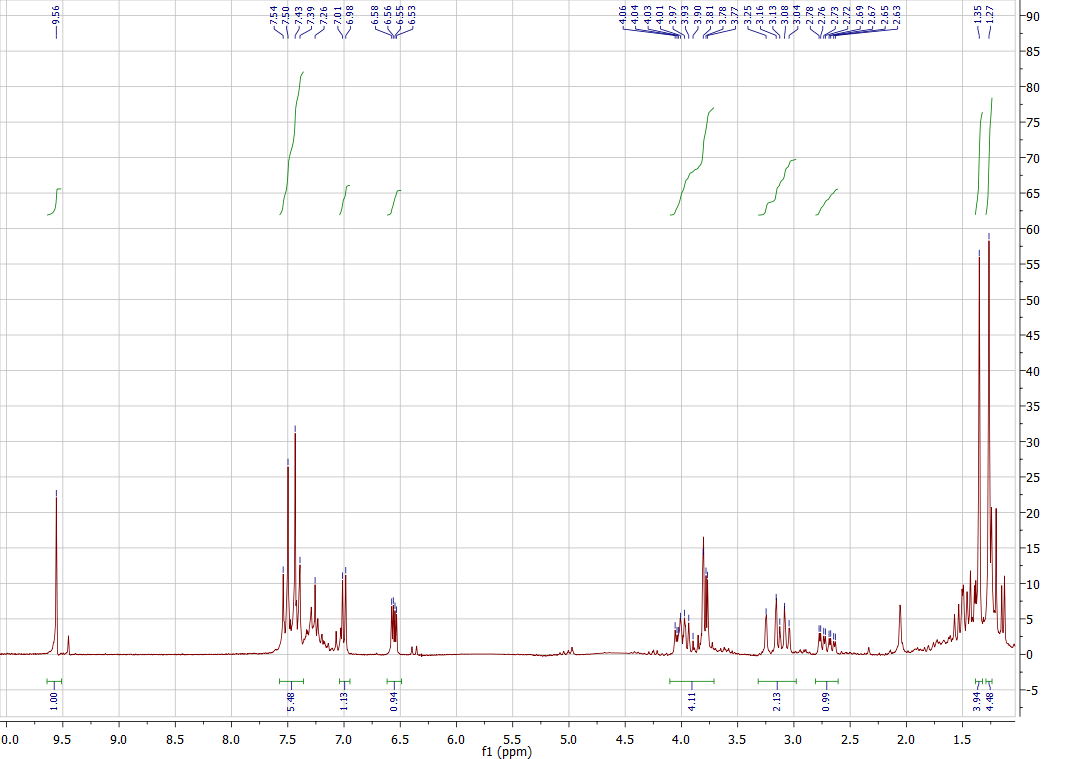
**

**
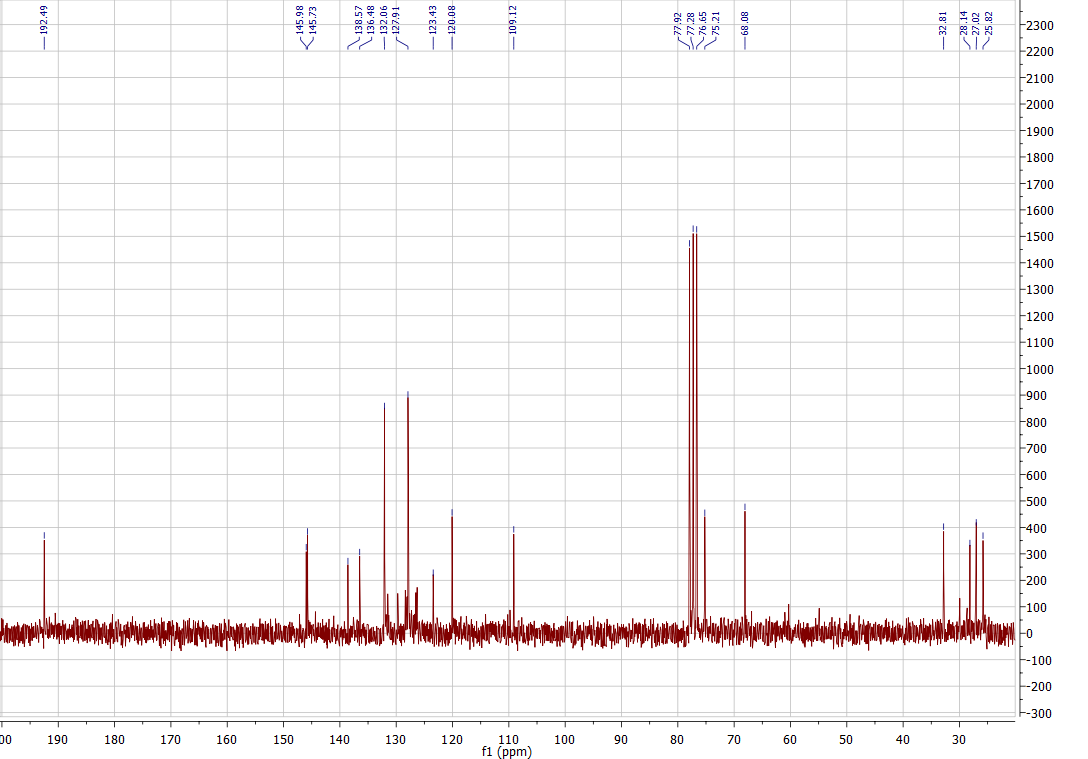
**

IR of **24b**

**
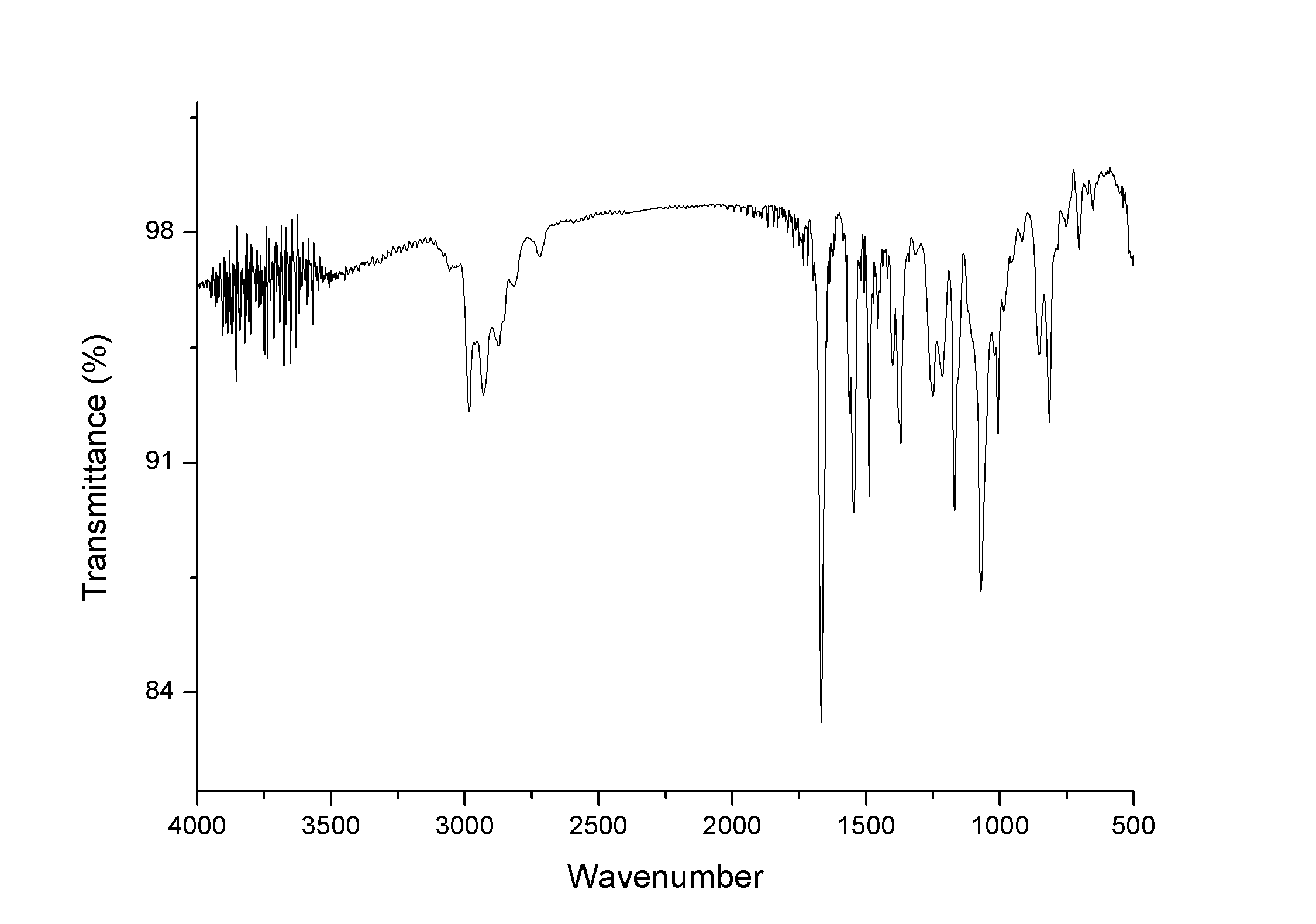
**

^1^H NMR and ^13^C NMR of **25a**


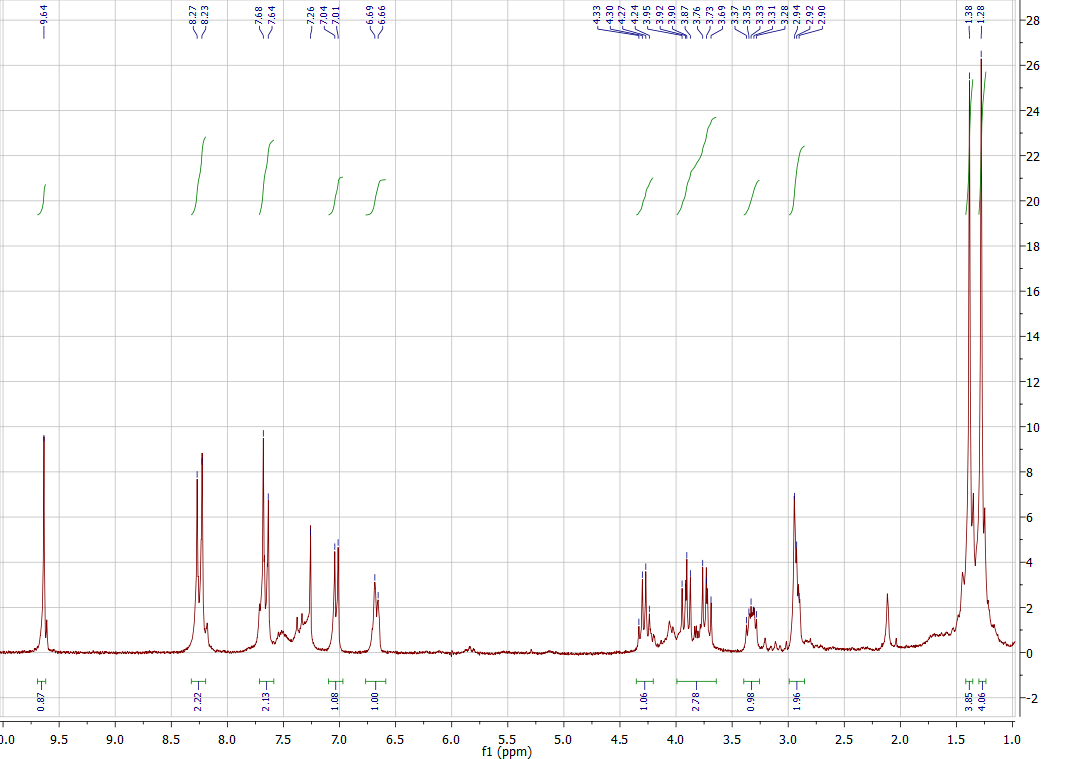


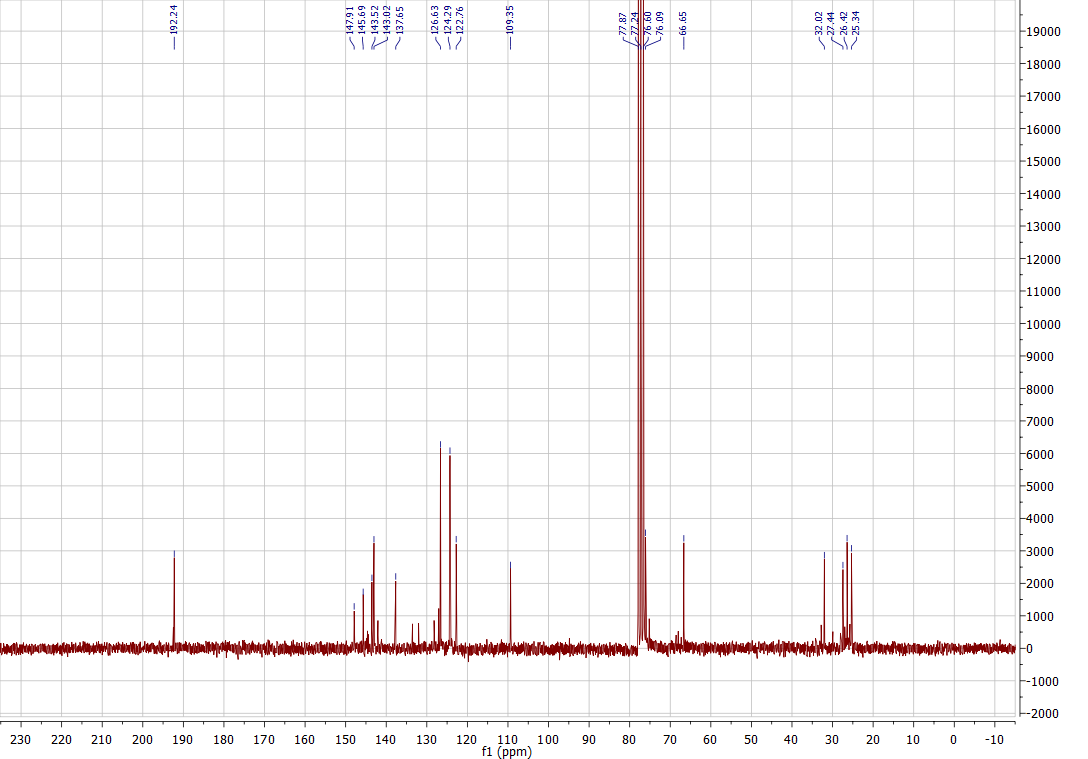


IR of **25a**

**
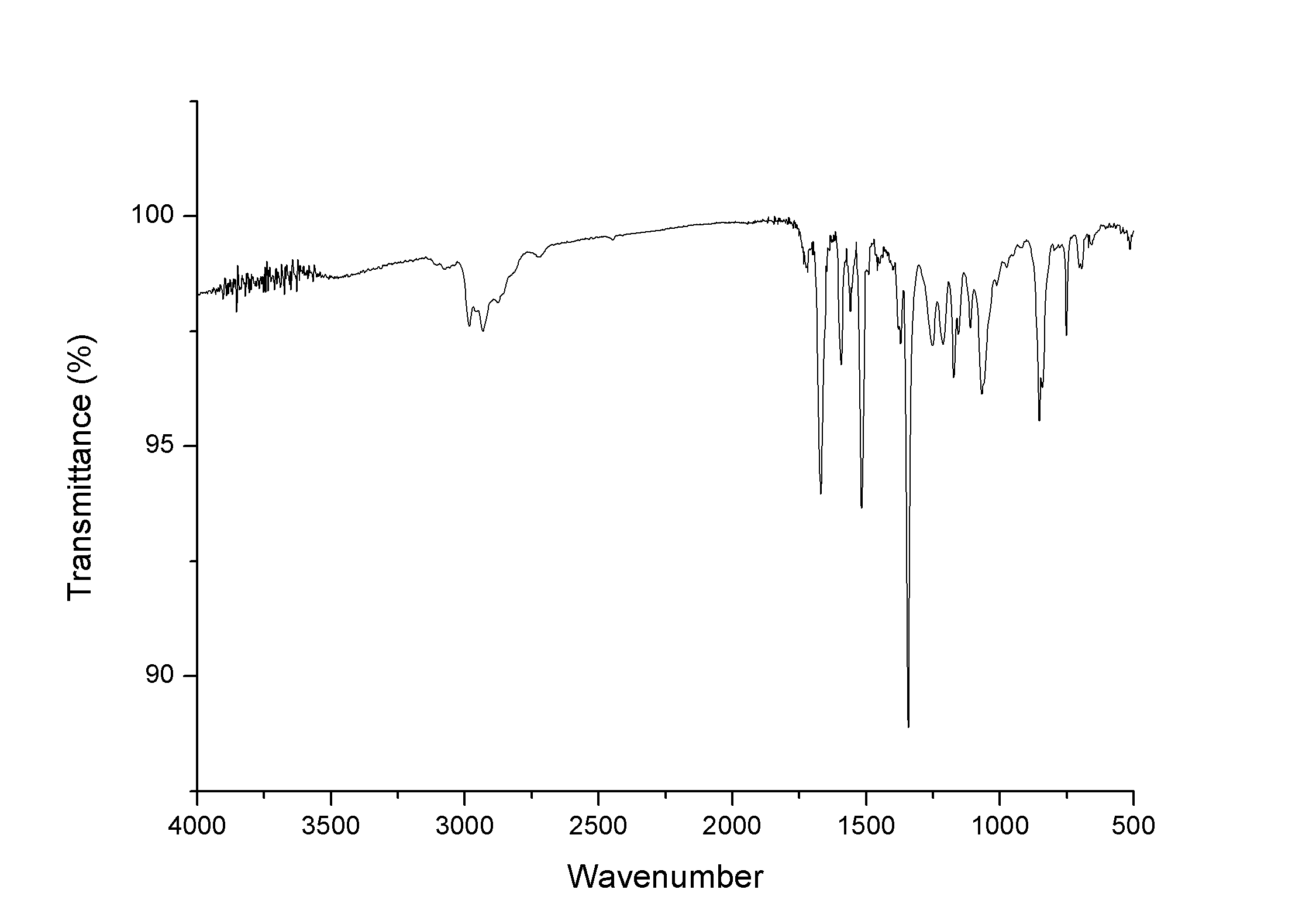
**

^1^H NMR and ^13^C NMR of **25b**


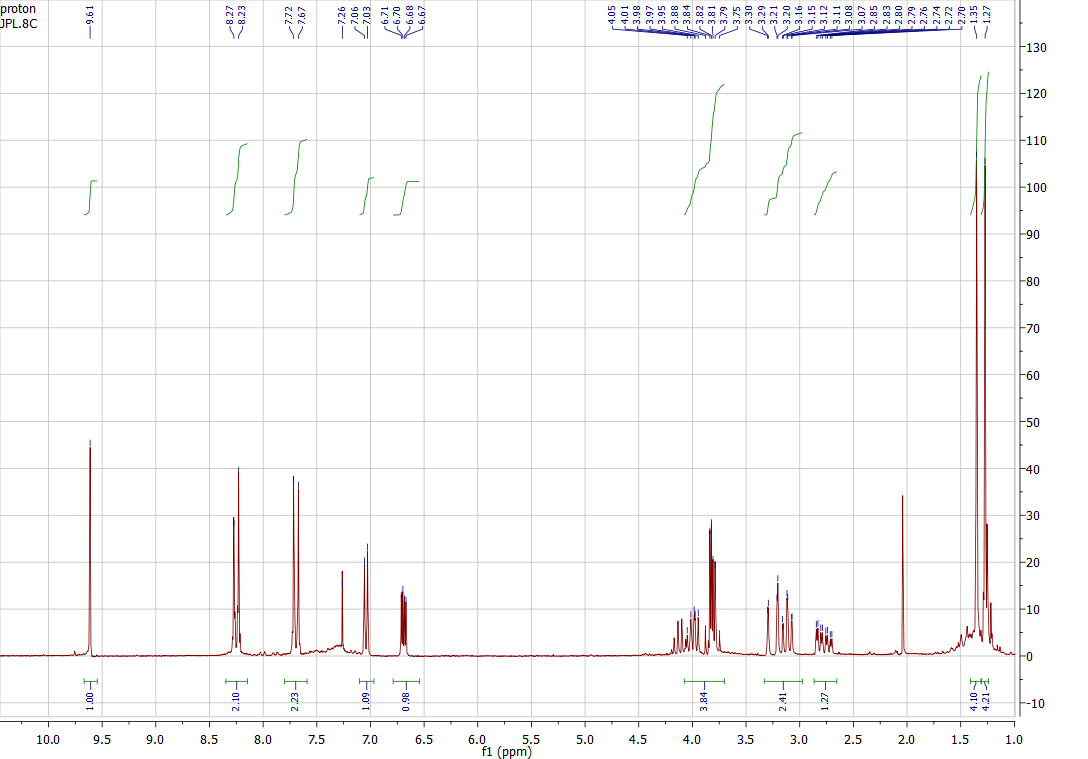


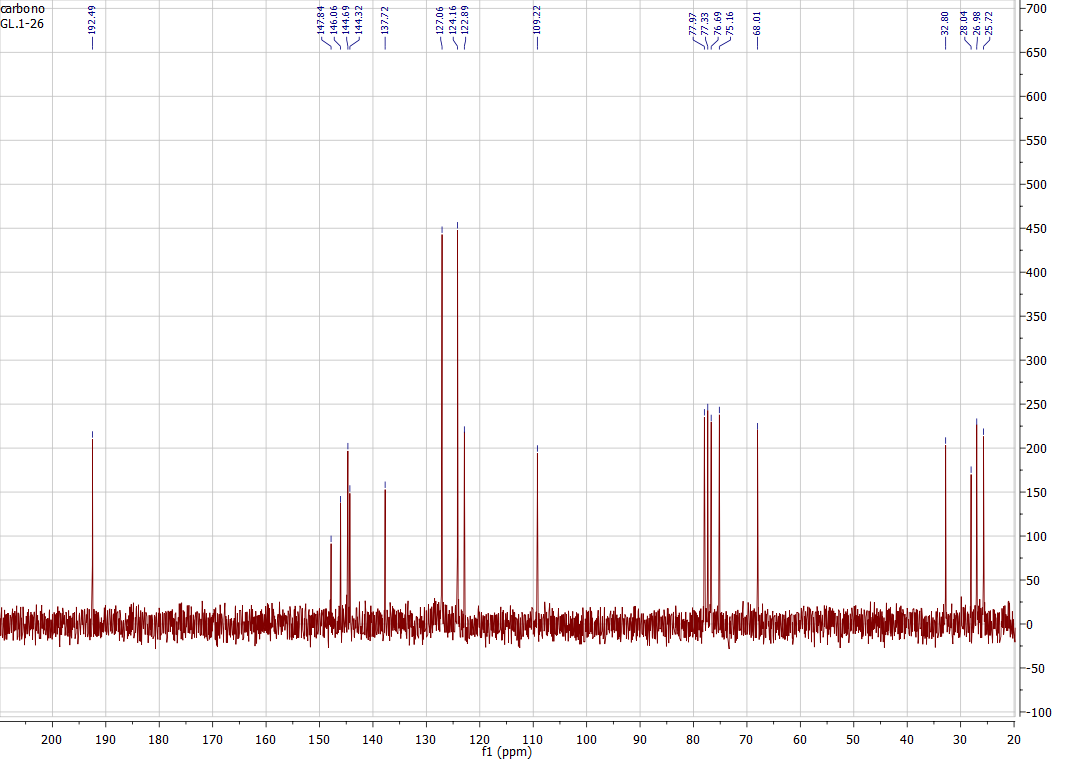


IR of **25b**

**
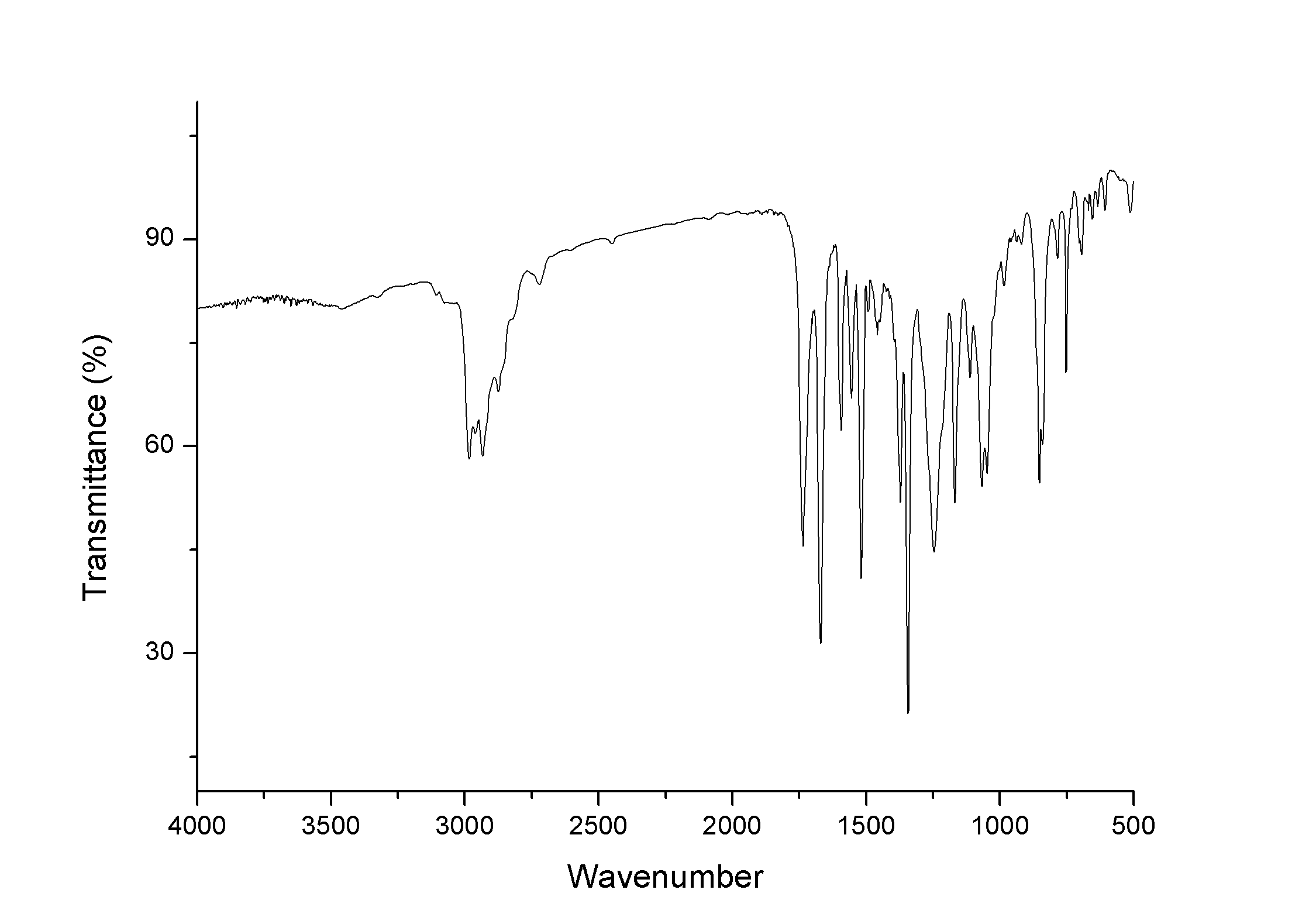
**

^1^H NMR and ^13^C NMR of **26**


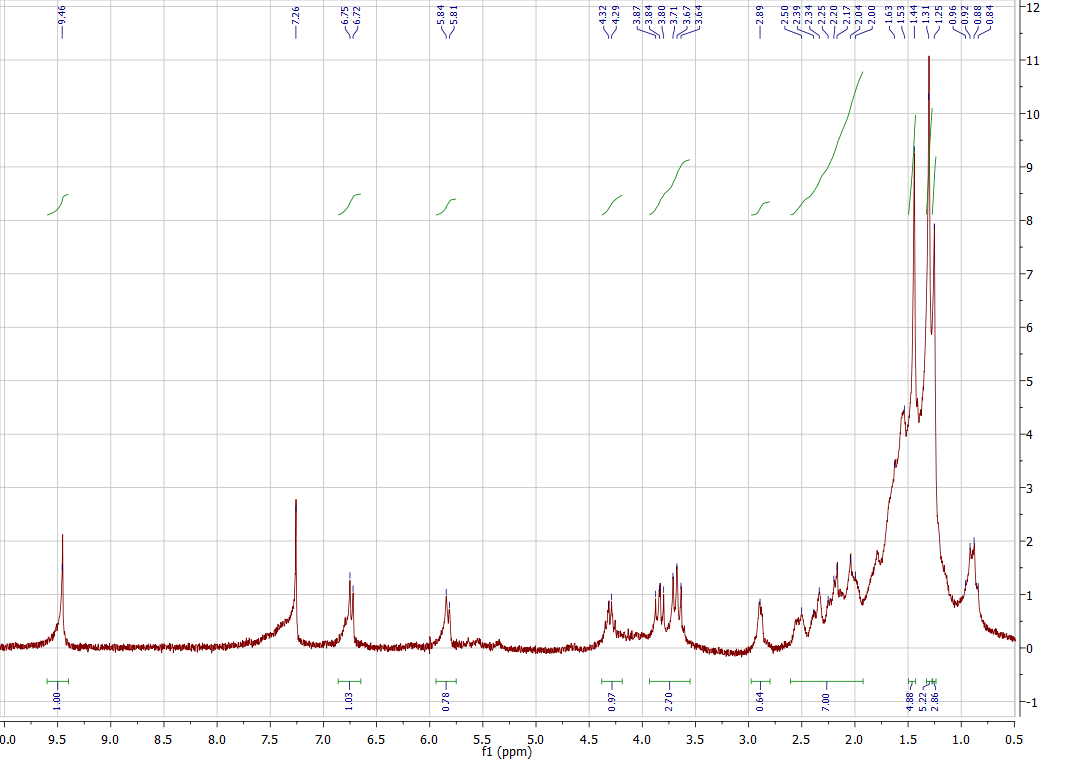


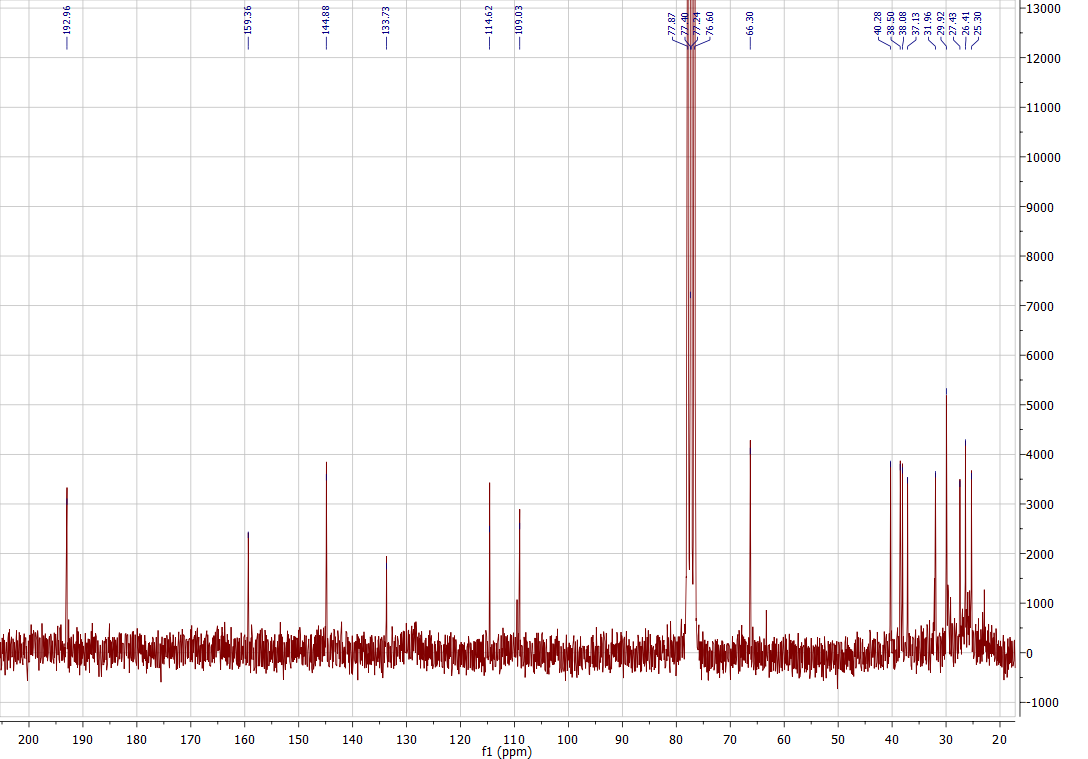


IR of **26**

**
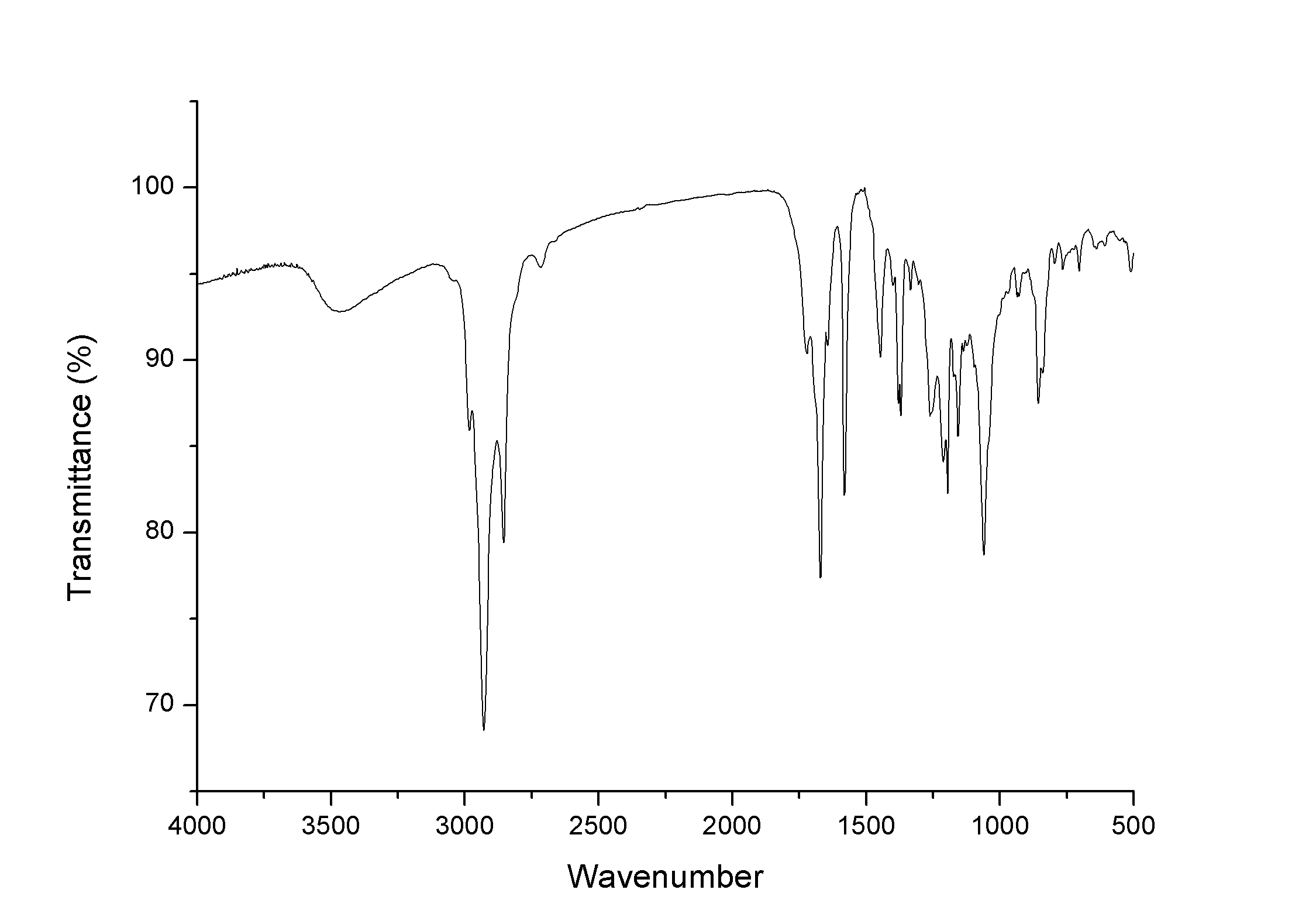
**
