# Supplementary material for: Diastereoselective synthesis of chiral 1,3-cyclohexadienals
Source: PLoS One. 2018 Feb 13;13(2):e0192113. doi: 10.1371/journal.pone.0192113 (PMC5810990; doi:10.1371/journal.pone.0192113)
Supplement: S2 File — (DOCX) [file pone.0192113.s002.docx]

**Diastereoselective Synthesis of Chiral 1,3-Cyclohexadienals**

Aitor Urosa^1¶^, Ignacio E. Tobal^1¶^, Ángela P. de la Granja^1^, M. Carmen Capitán^1^, R. F. Moro^1^, Isidro S. Marcos^1^, Narciso M. Garrido^1^, Francisca Sanz^2^, Emilio Calle^3^ and David Díez^1^*

^1^ Departamento de Química Orgánica, Facultad de Ciencias Químicas, Universidad de Salamanca, Salamanca. Spain.

^2^ Servicio de Difracción de Rayos X, Universidad de Salamanca, Salamanca, Spain.

^3^Departamento de Química Física, Facultad de Ciencias Químicas, Universidad de Salamanca, Salamanca. Spain.

*E-mail: [ddm@usal.es](mailto:ddm@usal.es)

¶ These authors contributed equally to this work.

**SUPPORTING INFORMATION 2**

**Experimental procedure for the synthesis of α,β-aldehyde intermediates**

| **Content** | **Page** |
| --- | --- |
| General Procedure of Synthesis of Aldehydes (**14-18**) | S2 |
| Synthesis of Aldehyde **19** | S6 |
| References | S7 |

**GENERAL PROCEDURE SYNTHESIS OF ALDEHYDES (14-18)**

Methyl Diethylphosphonoacetate (1.9 mL, 10.0 mmol) was added dropwise under argon atmosphere over a period of 5 min to a stirred suspension of NaH (60% on mineral oil; 500 mg, 10.0 mmol) in dry toluene (16.0 mL) at 0ºC, and the resulting mixture was stirred for 45 min at 0°C. A solution of ketone (10.0 mmol) in dry toluene (4.0 mL) was slowly added to the resulting mixture, and the reaction mixture was stirred at r.t. for 5h. After cooling to room temperature, the reaction was quenched with a saturated aqueous solution of NH_4_Cl and extracted with EtOAc (3x20 mL). The combined organic layer was washed with brine and dried over Na_2_SO_4_. After concentration under vacuum, the residue was purified by flash chromatography on silica gel (EtOAc:hexane) to afford (*E*)-oate and (*Z*)-oate as colorless oil.

DIBAL-H (4 equiv., 1.2 M in toluene) was added dropwise to a solution of ester in DCM (0.3 M) at −78°C. The reaction mixture was stirred at −78°C for 2h and the solution was allowed to reach 0°C. The solution was quenched with a saturated aqueous solution of potassium sodium tartrate at 0°C and stirred at room temperature for 3h. The resulting mixture was extracted with DCM (3x 30mL) and washed with brine. The resulting solution was dried over Na_2_SO_4_ and concentrated under vacuum. The residue was purified by column chromatography on silica gel (EtOAc:hexane) to obtain alcohol as colorless oil.

MnO_2_ (5 equiv) was added to a solution of alcohol in DCM (0.5 M) at r.t. The reaction mixture was stirred at r.t. for 24h. The suspension was filtered through a pad of Celite and washed with EtOAc. The resulting solution was concentrated under vacuum, the residue was purified by column chromatography on silica gel (EtOAc:hexane) to afford aldehyde as a light yellow oil.

All characterization data are consistent with literature values.

**Methyl (*Z,E*)-3-phenylbut-2-enoate, 14a.** [1]

**Yield:** 87% (1.5 g, 8.72 mmol).

**Z:E ratio:** 7:93.

**^1^H NMR** (300 MHz, CDCl_3_): δ=8.24-8.19 (2H, m, E isomer), 7.94-7-89 (2H, m, Z isomer), 7.62-7.57 (2H, m, E isomer), 7.21-7.16 (2H, m, Z isomer), 6.17 (1H, bs, E isomer), 3.76 (3H, s, E isomer), 3.54 (3H, s), 2.57 (3H, s, E isomer).

**(*Z,E*)-3-Phenyl-but-2-en-1-ol, 14b.**[2]

**Yield:** 88% (1.1 g, 7.63 mmol).

**Z:E ratio:** 2:98.

**^1^H NMR** (300 MHz, CDCl_3_): δ= 7.32-7.15 (10H, m), 5.96 (1H, td, *J* = 6.9, 1.5 Hz, E isomer), 5.70 (1H, td, *J* = 6.9, 1.2 Hz, Z isomer), 4.34 (2H, d, *J* = 6.9 Hz, E isomer), 4.05 (2H, d, *J* = 6.9 Hz, Z isomer), 2.07 (6H, bs, E isomer).

**(*Z,E*)-3-Phenyl-but-2-enal, 14.**[3]

**Yield:** 91% (1.0 g, 6.94 mmol).

**Z:E ratio:** 2:98.

**^1^H NMR** (300 MHz, CDCl_3_): δ= 10.18 (1H, d, *J* =7.5 Hz, E isomer), 9.47 (1H, d, *J* = 8.1 Hz, Z isomer), 7.55-7.29 (10H, m), 6.39 (1H, d, *J* = 7.5 Hz, E isomer), 6.13 (1H, d, *J* = 8.1 Hz, Z isomer), 2.56 (3H, s, E isomer), 2.30 (3H, s, Z isomer).

**HRMS** (ESI): Calculated for C_10_H_11_O ([M+H]^+^): 147.0804, found 147.0807.

**Methyl (*Z,E*)-3-(4-methylphenyl)but-2-enoate, 15a.**[4-6]

**Yield:** 63% (1.2 g, 6.30 mmol).

**Z:E ratio:** 15:85.

**^1^H NMR** (200 MHz, CDCl_3_): δ= 7.39 (2H, d, *J* = 8.4 Hz), 7.18 (2H, d, *J* = 8.4 Hz), 6.13 (1H, bs, E isomer), 5.89 (1H, bs, Z isomer), 3.74 (3H, s, E isomer), 3.57 (3H, s, Z isomer), 2.57 (3H, s), 2.36 (3H, s, E isomer), 2.17 (3H, s, Z isomer).

**HRMS** (ESI): Calculated for C_12_H_14_O_2_Na ([M+Na]^+^): 213.0886; found 213.0887.

**(*Z,E*)-3-(4-Methylphenyl)but-2-en-1-ol, 15b.**[2]

**Yield**: 91% (930 mg, 5.73 mmol).

**Z:E ratio:** 15:85.

**^1^H NMR** (200 MHz, CDCl_3_): δ= 7.32 (2H, d, *J* = 8.2 Hz), 7.14 (2H, d, *J* = 8.2 Hz), 5.96 (1H, t, *J* = 6.7 Hz, E isomer), 5.69 (1H, t, *J* = 7.0 Hz, Z isomer), 4.35 (2H, d, *J* = 6.7 Hz, E isomer), 4.09 (2H, d, *J* = 7.0 Hz, Z isomer), 2.35 (3H, s), 2.07 (3H, s, E isomer), 2.05 (3H, s, Z isomer).

**(*Z,E*)-3-(4-Methylphenyl)but-2-enal, 15.**[7]

**Yield**: 83% (762 mg, 4.76 mmol).

**Z:E ratio:** 2:98.

**^1^H NMR** (200 MHz, CDCl_3_): δ= 10.14 (1H, d, *J* = 7.9Hz, E isomer), 9.48 (1H, d, *J* = 8.2 Hz, Z isomer), 7.84 (2H, d, *J* = 8.2 Hz, Z isomer), 7.46 (2H, d, *J* = 8.3 Hz, E isomer), 7.22 (1H, d, *J* = 8.3 Hz, E isomer), 6.40 (1H, dd, *J* = 7.9, 1.4 Hz, E isomer), 2.55 (3H, s, E isomer), 2.39 (3H, s, E isomer).

**Methyl (*Z,E*)-3-(3-bromophenyl)but-2-enoate, 16a.** [8]

**Yield**: 89 % (2.3 g, 8.90 mmol).

**Z:E ratio:** 12:88.

**^1^H NMR** (200 MHz, CDCl_3_): δ= 7.60-7.10 (8H, m), 6.07 (1H, bs, E isomer), 5.89 (1H, bs, Z isomer), 3.71 (3H, s, E isomer), 3.53 (3H, s, Z isomer), 2.54 (3H, s, Z isomer), 2.50 (3H, s, E isomer).

**(*Z,E*)-3-(3-Bromophenyl)but-2-en-1-ol, 16b.** [9]

**Yield**: 81% (1.6 g, 7.20 mmol).

**Z:E ratio:** 12:88.

**^1^H NMR** (200 MHz, CDCl_3_): δ= 7.52-7.12 (8H, m), 5.92 (1H, t, *J* = 6.5 Hz, E isomer), 5.68 (1H, t, *J* = 6.9 Hz, Z isomer), 4.31 (2H, t, *J* = 6.5 Hz, E isomer), 4.01 (2H, t, *J* = 6.9 Hz, Z isomer), 2.58 (1H, bs, OH), 2.32 (1H, bs, OH), 2.03 (3H, s, Z isomer), 2.00 (3H, s, E isomer).

**(*Z,E*)-3-(3-Bromopheynyl)but-2-enal, 16.** [3]

**Yield**: 61% (1.0 g, 4.42 mmol).

**Z:E ratio:** 5:95.

**^1^H NMR** (200 MHz, CDCl_3_): δ= 10.16 (1H, d, *J* = 7.7 Hz, E isomer), 9.45 (1H, d, *J* = 8.4 Hz, Z isomer), 7,66 (1H, bs), 7.49 (2H, dd, *J* = 16.5, 7.9 Hz), 7.30 (1H, d, *J* = 7.9 Hz), 7.26 (1H, d, *J* = 9.4 Hz), 6.34 (1H, d, *J* = 7.7 Hz, E isomer), 6.14 (1H, d, *J* = 8.4 Hz, Z isomer), 2.54 (3H, s, E isomer), 2.29 (3H, s, Z isomer).

**Methyl (*Z,E*)-3-(4-bromophenyl)but-2-enoate, 17a.** [10]

**Yield**: 64% (1.6 g, 6.40 mmol).

**Z:E ratio:** 22:78.

**^1^H NMR** (200 MHz, CDCl_3_): δ= 7.51 (2H, d, *J* = 8.8 Hz, E isomer), 7.34 (2H, d, *J* = 8.8 Hz, E isomer), 7.30-7.10 (4H, m, Z isomer), 6.14 (1H, bs, E isomer), 5.95 (1H, bs, Z isomer), 3.77 (3H, s, E isomer), 3.59 (3H, s, Z isomer), 2.58 (3H, s, E isomer), 2.37(3H, s, Z isomer).

**(*Z,E*)-3-(4-Bromophenyl)but-2-en-1-ol, 17b.** [9]

**Yield**: 90% (1.3 g, 5.76 mmol).

**Z:E ratio:** 16:84.

**^1^H NMR** (200 MHz, CDCl_3_): δ= 7.44 (2H, d, *J* = 8.5 Hz, E isomer), 7.26 (2H, d, *J* = 8.5 Hz, E isomer), 7.05 (2H, d, *J* = 8.4 Hz, Z isomer), 5.96 (1H, t, *J* = 6.5 Hz, E isomer), 5.72 (1H, t, *J* = 7.0 Hz, Z isomer), 4.35 (2H, d *J* = 6.5 Hz, E isomer), 4.04 (2H, d, *J* = 7.0 Hz, Z isomer), 2.04 (3H, s, E isomer), 2.01 (3H, s, Z isomer).

**(*Z,E*)-3-(4-Bromophenyl)but-2-enal, 17.** [11]

**Yield**: 69% (895 mg, 3.97 mmol).

**Z:E ratio:** 6:94.

**^1^H NMR** (200 MHz, CDCl_3_): δ= 10.15 (1H, d, *J* = 7.8 Hz, E isomer), 9.45 (1H, d *J* = 8.2 Hz, Z isomer), 7.53 (2H, d, *J* = 8.6 Hz, E isomer), 7.39 (2H, d, *J* = 8.6 Hz, E isomer), 7.16 (2H, d, *J* = 8.3 Hz, Z isomer), 6.35 (1H, d, *J* = 7.8 Hz, E isomer), 6.13 (1H, d, *J* = 8.2 Hz, Z isomer), 2.53 (3H, s, E isomer), 2.33 (3H, s, Z isomer).

**Methyl (*E*)-3-(4-nitrophenyl)but-2-enoate, 18a.** [12]

**Yield**: 69% (1.5 g, 6.90 mmol).

**^1^H NMR** (200 MHz, CDCl_3_): δ= 8.24 (2H, d, *J* = 8.7 Hz), 7.61 (2H, d, *J* = 8.7 Hz), 6.19 (1H, d, *J* = 1.4 Hz), 3.79 (3H, s), 2.60 (3H, d, *J* = 1.4 Hz).

**(*E*)-3-(4-Nitrophenyl)but-2-en-1-ol, 18b.** [13]

**Yield**: 99% (1.3 g, 6.83 mmol).

**^1^H NMR** (200 MHz, CDCl_3_): δ= 8.19 (2H, d, *J* = 8.9 Hz), 7.54 (2H, d, *J* = 8.9 Hz), 6.11 (1H, t, *J* = 6.5 Hz), 4.42 (2H, t, *J* = 5.1 Hz), 2.11 (3H, s).

**(*E*)-3-(4-Nitrophenyl)but-2-enal, 18.** [14]

**Yield**: 97% (1.3 g, 6.63 mmol).

**^1^H NMR** (200 MHz, CDCl_3_): δ= 9.42 (1H, d, *J* = 8.2 Hz), 8.29 (2H, d, *J* = 8.7 Hz), 7.49 (2H, d, *J* = 8.7 Hz), 6.21 (1H, dd, *J* = 8.2, 1.5 Hz), 2.34 (3H, d, *J* = 1.5 Hz).

**SYNTHESIS OF ALDEHYDE 19**

Methyl Diethylphosphonoacetate (5.8 mL, 29.4 mmol) was added dropwise under argon atmosphere over a period of 5 min to a stirred suspension of NaH (60% on mineral oil; 1130 mg, 29.4 mmol) in dry toluene (18 mL) at 0ºC, and the resulting mixture was stirred for 45 min at 0°C. A solution of cyclohexanone (2.6 mmol) in dry toluene (9.0 mL) was slowly added to the resulting mixture, and the reaction mixture was stirred at r.t. for 5h. After cooling to room temperature, the reaction was quenched with a saturated aqueous solution of NH_4_Cl and extracted with EtOAc (3x20 mL). The combined organic layer was washed with brine and dried over Na_2_SO_4_. After concentration under vacuum, the residue was purified by flash chromatography on silica gel (EtOAc:hexane) to afford **19a** (95%, 471 mg, 2.53mmol) as colorless oil.

**Methyl 2-cyclohexylideneacetate, 19a.** [15]

**^1^H NMR** (200 MHz, CDCl_3_): δ= 5.56 (1H, s), 3.63 (3H, s), 2.81-2.76 (2H, m), 2.18-2.12 (2H, m), 1.59 (6H, bs).

All characterization data are consistent with literature values.

DIBAL-H (3.7 mL, 1.5 M in toluene) was added dropwise to a solution of **19a** (451 mg, 2.53 mmol) in DCM (25.3 mL) at −78°C. The reaction mixture was stirred at −78°C for 2h and the solution was allowed to reach 0°C. The solution was quenched with a saturated aqueous solution of potassium sodium tartrate at 0°C and stirred at room temperature for 3h. The resulting mixture was extracted with EtOAc (3x50 mL) and washed with brine. The resulting solution was dried over Na_2_SO_4_ and concentrated under vacuum to obtain **19b** (67%, 266 mg, 1.70 mmol) as colorless oil.

**2-Cyclohexylideneethan-1-ol, 19b.** [2]

**^1^H NMR** (200 MHz, CDCl_3_): δ= 5.30 (1H, t, *J* = 7.2 Hz), 4.08 (2H, d, *J* = 7.2 Hz), 2.20-2.00 (4H, m), 1.51 (6H, bs).

All characterization data are consistent with literature values.

To a solution of **19b** (266mg, 1.70 mmol) in DCM (8.5 mL), PDC (770 mg, 2.04 mmol) and NaHCO_3_ (186mg, mmol) were added. The reaction mixture was stirred under Ar atmosphere at room temperature for 3h. Then, the resulting suspension was filtered through pad of Celite washing with DCM. The filtrate was evaporated under vacuum to obtain **19** (54%, 140 mg, 0.91 mmol) as a colourless oil.

**2-Cyclohexylideneacetaldehyde, 19.** [16]

**^1^H NMR** (200 MHz, CDCl_3_): δ= 9.97 (1H, d, *J* = 8.4 Hz), 5.79 (1H, d, *J* = 8.4 Hz), 2.67 (2H, bt, *J* = 5.4 Hz), 2.26 (2H, t, *J* = 5.4 Hz), 1.74-1.59 (6H, m).

**HRMS** (ESI): Calculated for C_8_H_13_O [(M+H)^+^]: 125.0961; found 125.0966.

All characterization data are consistent with literature values.

**REFERENCES**

1. Maji T, Karmakar A, Reiser O. Visible-Light Photoredox Catalysis: Dehalogenation of Vicinal Dibromo-, α-Halo-, and α,α-Dibromocarbonyl Compounds. The Journal of Organic Chemistry. 2011;76(2):736-9.

2. Bernasconi M, Ramella V, Tosatti P, Pfaltz A. Iridium-Catalyzed Asymmetric Hydrogenation of 3,3-Disubstituted Allylic Alcohols in Ethereal Solvents. Chemistry – A European Journal. 2014;20(9):2440-4.

3. Song A, Zhang X, Song X, Chen X, Yu C, Huang H, et al. Construction of Chiral Bridged Tricyclic Benzopyrans: Enantioselective Catalytic Diels–Alder Reaction and a One-Pot Reduction/Acid-Catalyzed Stereoselective Cyclization. Angewandte Chemie International Edition. 2014;53(19):4940-4.

4. Metternich JB, Gilmour R. A Bio-Inspired, Catalytic E → Z Isomerization of Activated Olefins. Journal of the American Chemical Society. 2015;137(35):11254-7.

5. Guzman-Martinez A, Hoveyda AH. Enantioselective Synthesis of Allylboronates Bearing a Tertiary or Quaternary B-Substituted Stereogenic Carbon by NHC-Cu-Catalyzed Substitution Reactions. Journal of the American Chemical Society. 2010;132(31):10634-7.

6. Ren K, Hu B, Zhao M, Tu Y, Xie X, Zhang Z. Ruthenium-Catalyzed Oxidation of Allyl Alcohols with Intermolecular Hydrogen Transfer: Synthesis of α,β-Unsaturated Carbonyl Compounds. The Journal of Organic Chemistry. 2014;79(5):2170-7.

7. Castagnolo D, Botta L, Botta M. Alkyne-Enol Ether Cross-Metathesis in the Presence of CuSO4: Direct Formation of 3-Substituted Crotonaldehydes in Aqueous Medium. The Journal of Organic Chemistry. 2009;74(8):3172-4.

8. Shevlin M, Friedfeld MR, Sheng H, Pierson NA, Hoyt JM, Campeau L-C, et al. Nickel-Catalyzed Asymmetric Alkene Hydrogenation of α,β-Unsaturated Esters: High-Throughput Experimentation-Enabled Reaction Discovery, Optimization, and Mechanistic Elucidation. Journal of the American Chemical Society. 2016;138(10):3562-9.

9. Zi W, Wang Y-M, Toste FD. An In Situ Directing Group Strategy for Chiral Anion Phase-Transfer Fluorination of Allylic Alcohols. Journal of the American Chemical Society. 2014;136(37):12864-7.

10. Ruan J, Li X, Saidi O, Xiao J. Oxygen and Base-Free Oxidative Heck Reactions of Arylboronic Acids with Olefins. Journal of the American Chemical Society. 2008;130(8):2424-5.

11. Johansen TK, Gómez CV, Bak JR, Davis RL, Jørgensen KA. Organocatalytic Enantioselective Cycloaddition Reactions of Dienamines with Quinones. Chemistry – A European Journal. 2013;19(49):16518-22.

12. Dubbaka SR, Vogel P. Palladium-Catalyzed Desulfitative Mizoroki–Heck Couplings of Sulfonyl Chlorides with Mono- and Disubstituted Olefins: Rhodium-Catalyzed Desulfitative Heck-Type Reactions under Phosphine- and Base-Free Conditions. Chemistry – A European Journal. 2005;11(9):2633-41.

13. Li J-Q, Liu J, Krajangsri S, Chumnanvej N, Singh T, Andersson PG. Asymmetric Hydrogenation of Allylic Alcohols Using Ir–N,P-Complexes. ACS Catalysis. 2016;6(12):8342-9.

14. Stadler M, List B. Heck Reactions of Crotonaldehyde. Synlett. 2008;2008(04):597-9.

15. Strick BF, Mundal DA, Thomson RJ. An Oxidative [2,3]-Sigmatropic Rearrangement of Allylic Hydrazides. Journal of the American Chemical Society. 2011;133(36):14252-5.

16. Ouellet SG, Tuttle JB, MacMillan DWC. Enantioselective Organocatalytic Hydride Reduction. Journal of the American Chemical Society. 2005;127(1):32-3.
