# Supplementary material for: Diastereoselective synthesis of chiral 1,3-cyclohexadienals
Source: PLoS One. 2018 Feb 13;13(2):e0192113. doi: 10.1371/journal.pone.0192113 (PMC5810990; doi:10.1371/journal.pone.0192113)

**Diastereoselective Synthesis of Chiral 1,3-Cyclohexadienals**

Aitor Urosa^1¶^, Ignacio E. Tobal^1¶^, Angela P. de la Granja^1^, M. Carmen Capitán^1^, R. F. Moro^1^, Isidro S. Marcos^1^, Narciso M. Garrido^1^, Francisca Sanz^2^, Emilio Calle^3^ and David Díez^1^*

^1^ Departamento de Química Orgánica, Facultad de Ciencias Químicas, Universidad de Salamanca, Salamanca. Spain.

^2^ Servicio de Difracción de Rayos X, Universidad de Salamanca, Salamanca, Spain.

^3^ Departamento de Química Física, Facultad de Ciencias Químicas, Universidad de Salamanca, Salamanca. Spain.

*E-mail: [ddm@usal.es](mailto:ddm@usal.es)

¶ These authors contributed equally to this work.

**SUPPORTING INFORMATION 3**

**X-Ray crystallographic data**

**X-RAY CHRISTALLOGRAPHIC DATA OF 24a**

Suitable single crystals of the X, and Z compounds were mounted on glass fibre for data collection on a Bruker Kappa APEX II CCD diffractometer. Data were collected at 298(2) K using Cu Kα radiation (λ = 1.54178 Å) and ω scan technique, and were corrected for Lorentz and polarization effects. Structure solution, refinement and data output were carried out with the SHELXTLTM program package. The structure was solved by direct methods combined with difference Fourier synthesis and refined by full-matrix least-squares procedures, with anisotropic thermal parameters in the last cycles of refinement for all non-hydrogen atoms. H atoms of SP3 hybridized carbons were located directly in a difference Fourier map and freely refined. The rest of the hydrogen atoms were positioned geometrically.

CCDC 976522 contain the supplementary crystallographic data for this paper and can be obtained free of charge via www.ccdc.cam.ac.uk/conts/retrieving.html (or from the Cambridge Crystallographic Data Centre, 12, Union Road, Cambridge CB2 1EZ, UK; fax: +44-1223-336033; or deposit@ccdc.cam.ac.uk).

*Crystal data for compound* ***24a***: C_18_H_19_BrO_3_, *M* = 363.24, triclinic, space group P1 (No. 1), *a* = 6.9678(2) Å, *b* = 7.3292(2) Å, *c* = 9.1211(3) Å, *α* = 97.468(2)°, *β* = 92.051(2)°, *γ* = 115.800(2)°, *V* = 413.58(2) Å^3^, *Z* = 1, D_c_ = 1.458 mg/m^3^, *m* = (Cu-K_α_) = 3.468 mm-1, F(000) = 186. 2936 reflections were collected at 4.92 ≤ 2θ ≤ 66.81 and merged to give 1912 unique reflections (R_int_ = 0.0242), of which 1852 with I > 2σ (I) were considered to be observed. Final values are R = 0.0290, *w*R = 0.0744, GOF = 1.048, max/min residual electron density 0.217 and -0.219 e. Å^-3^

*ORTEP diagram for compound* ***24a***:


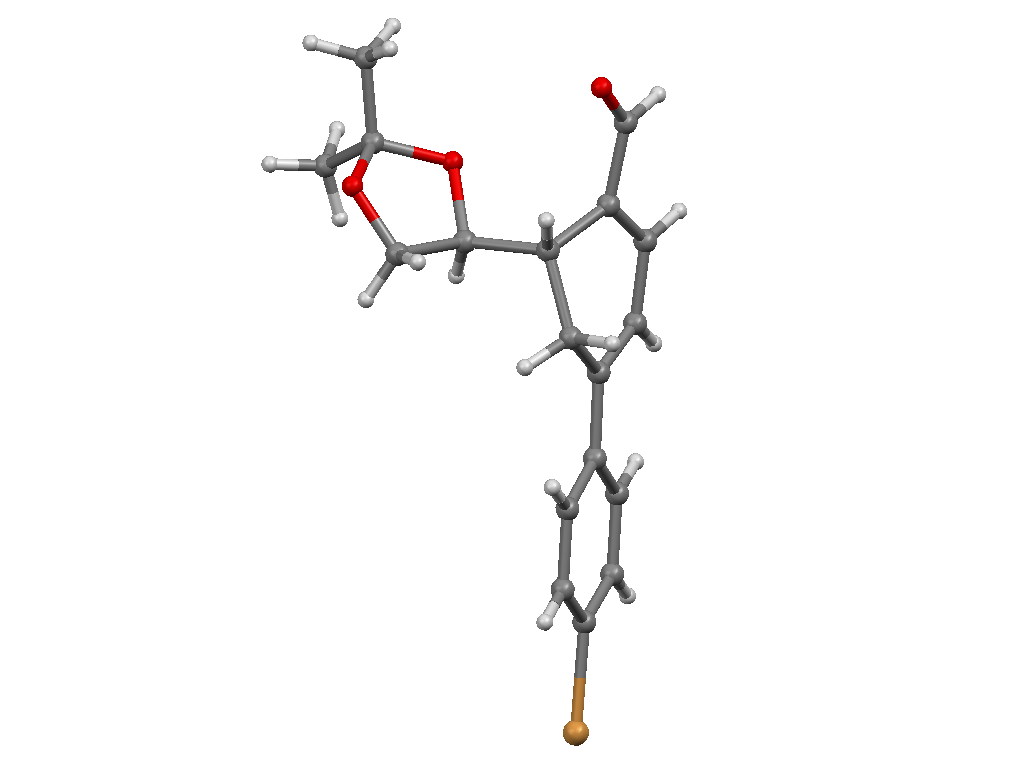


*Packing of compound* ***24a*** *along b axis*:


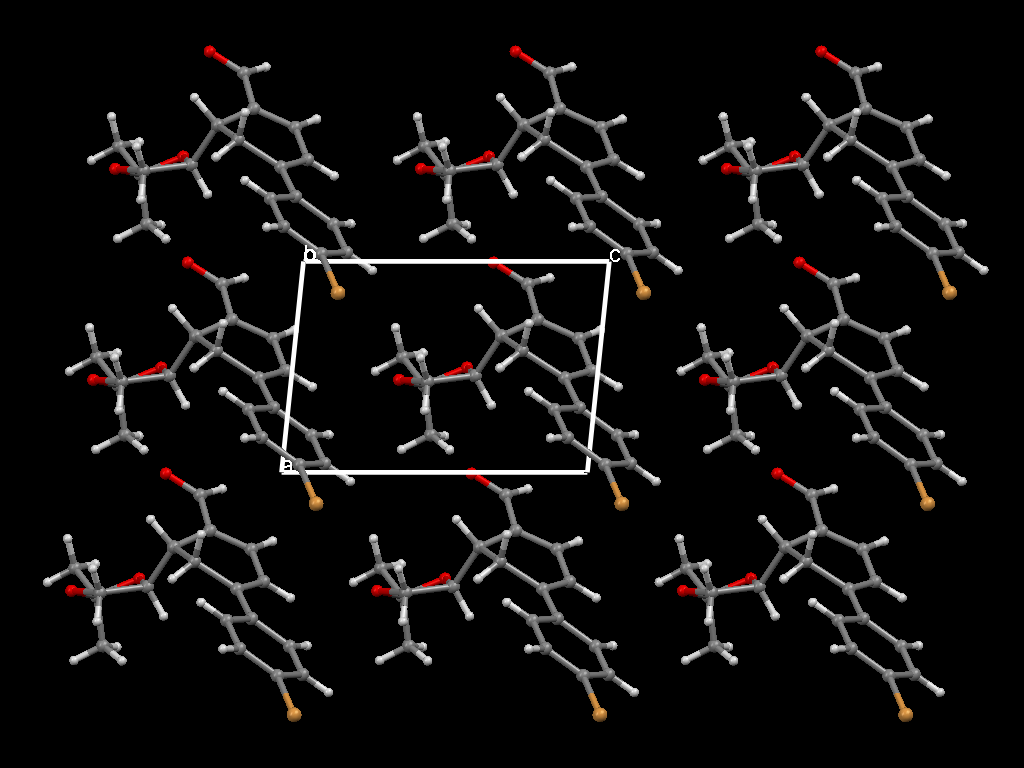

Supplement: S3 File — (DOCX) [file pone.0192113.s003.docx]
