# Supplementary material for: Diastereoselective synthesis of chiral 1,3-cyclohexadienals
Source: PLoS One. 2018 Feb 13;13(2):e0192113. doi: 10.1371/journal.pone.0192113 (PMC5810990; doi:10.1371/journal.pone.0192113)
Supplement: S4 File — (DOCX) [file pone.0192113.s004.docx]

**Diastereoselective Synthesis of Chiral 1,3-Cyclohexadienals**

Aitor Urosa^1¶^, Ignacio E. Tobal^1¶^, Angela P. de la Granja^1^, M. Carmen Capitán^1^, R. F. Moro^1^, Isidro S. Marcos^1^, Narciso M. Garrido^1^, Francisca Sanz^2^, Emilio Calle^3^ and David Díez^1^*

^1^ Departamento de Química Orgánica, Facultad de Ciencias Químicas, Universidad de Salamanca, Salamanca. Spain.

^2^ Servicio de Difracción de Rayos X, Universidad de Salamanca, Salamanca, Spain.

^3^ Departamento de Química Física, Facultad de Ciencias Químicas, Universidad de Salamanca, Salamanca. Spain.

*E-mail: [ddm@usal.es](mailto:ddm@usal.es)

¶ These authors contributed equally to this work.

**SUPPORTING INFORMATION 4**

**UV-Vis spectra**

**UV-VIS ABSORBANCE DATA**

**UV-Vis Spectra of 4a**

**
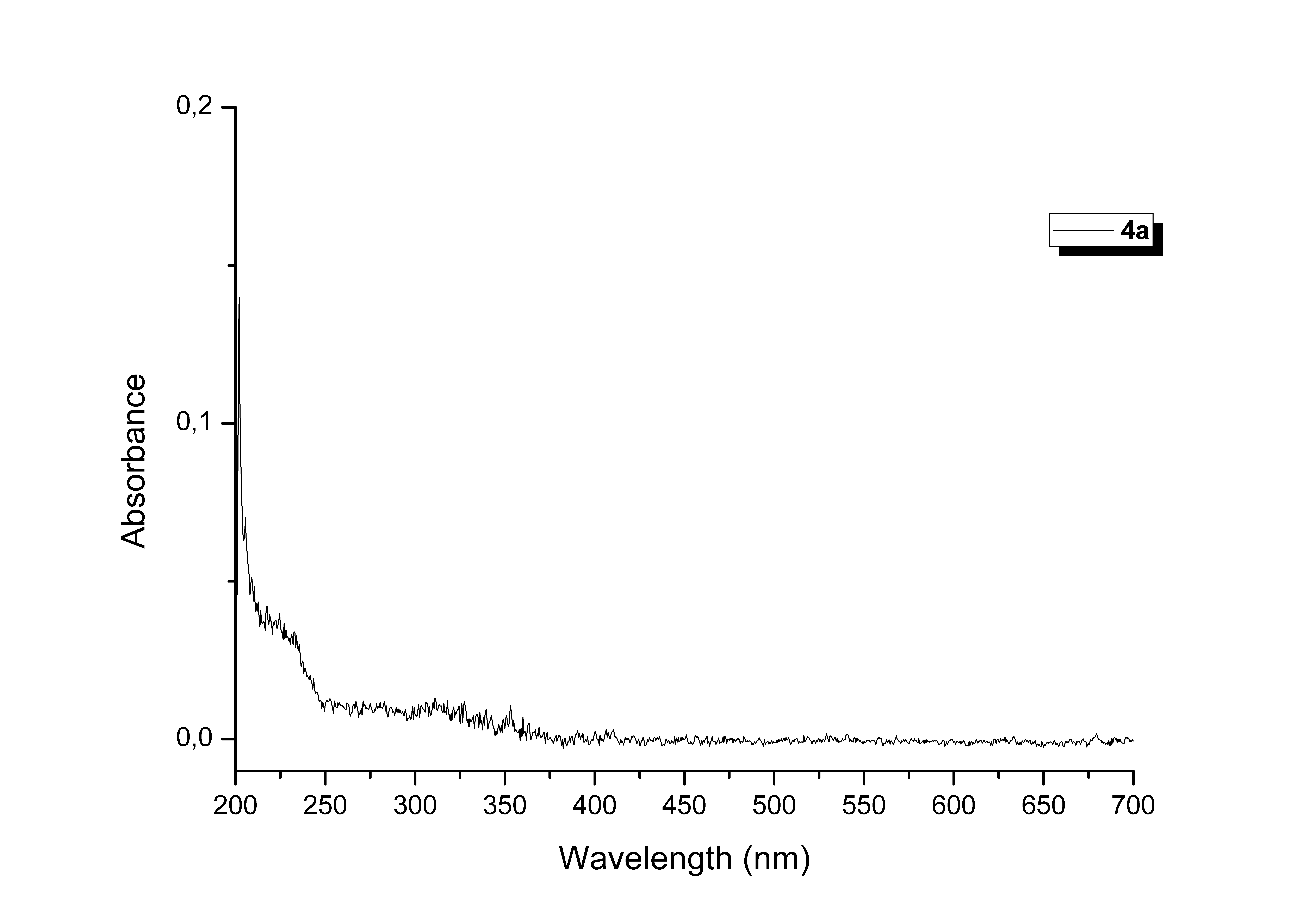
**

**UV-Vis Spectra of 20b**

**
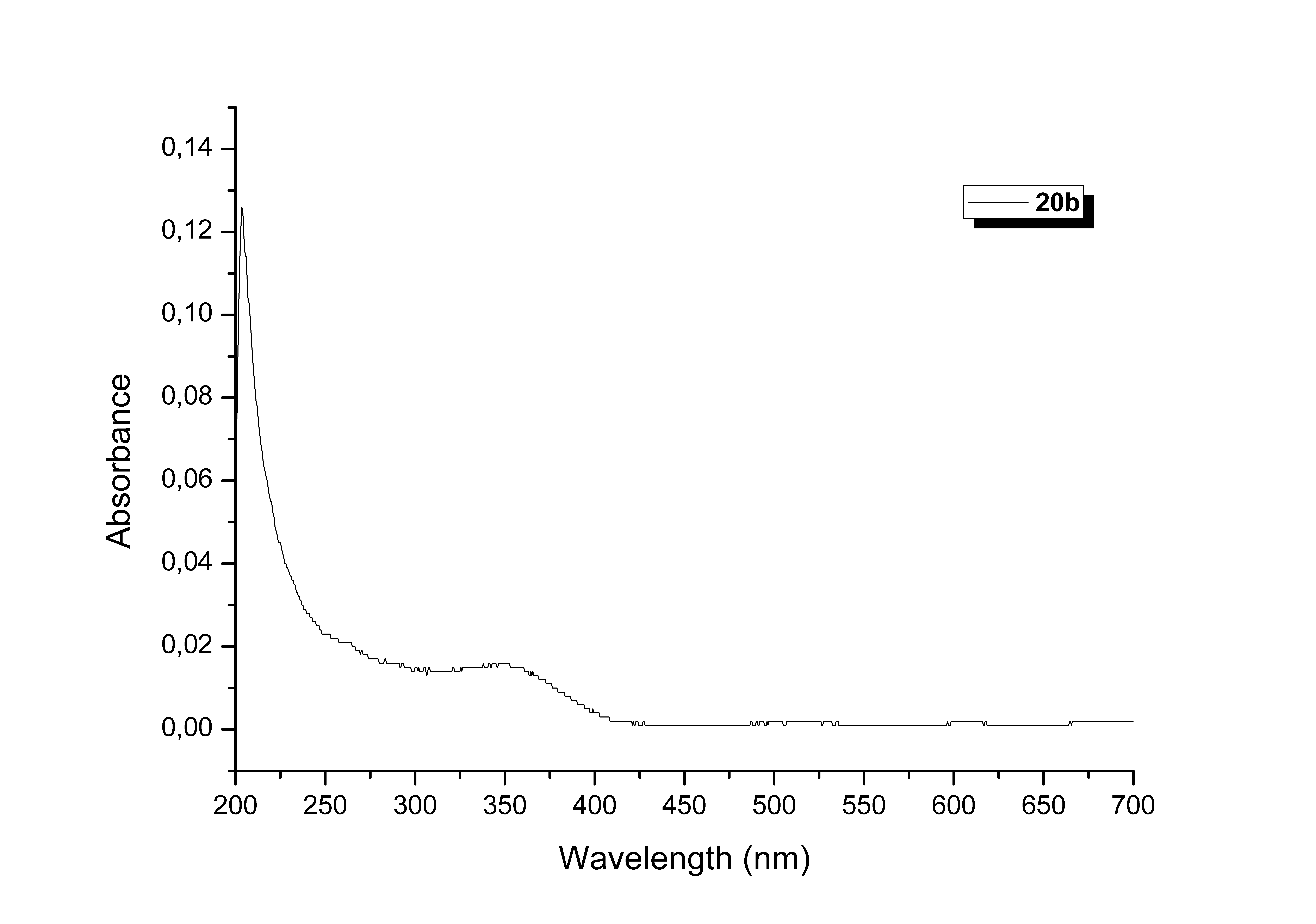
**

**UV-Vis Spectra of 21b**

**
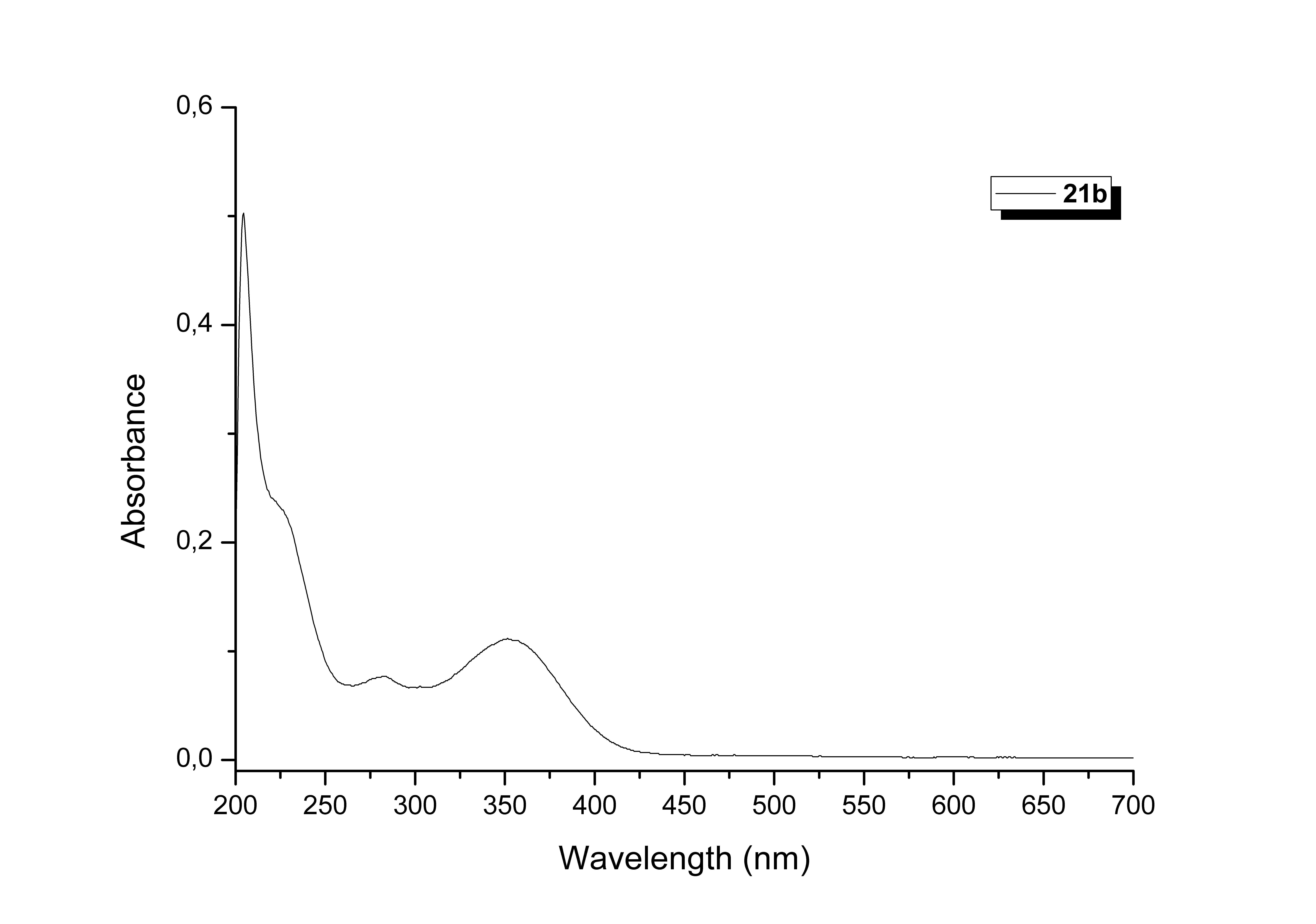
**

**UV-Vis Spectra of 22b**

**
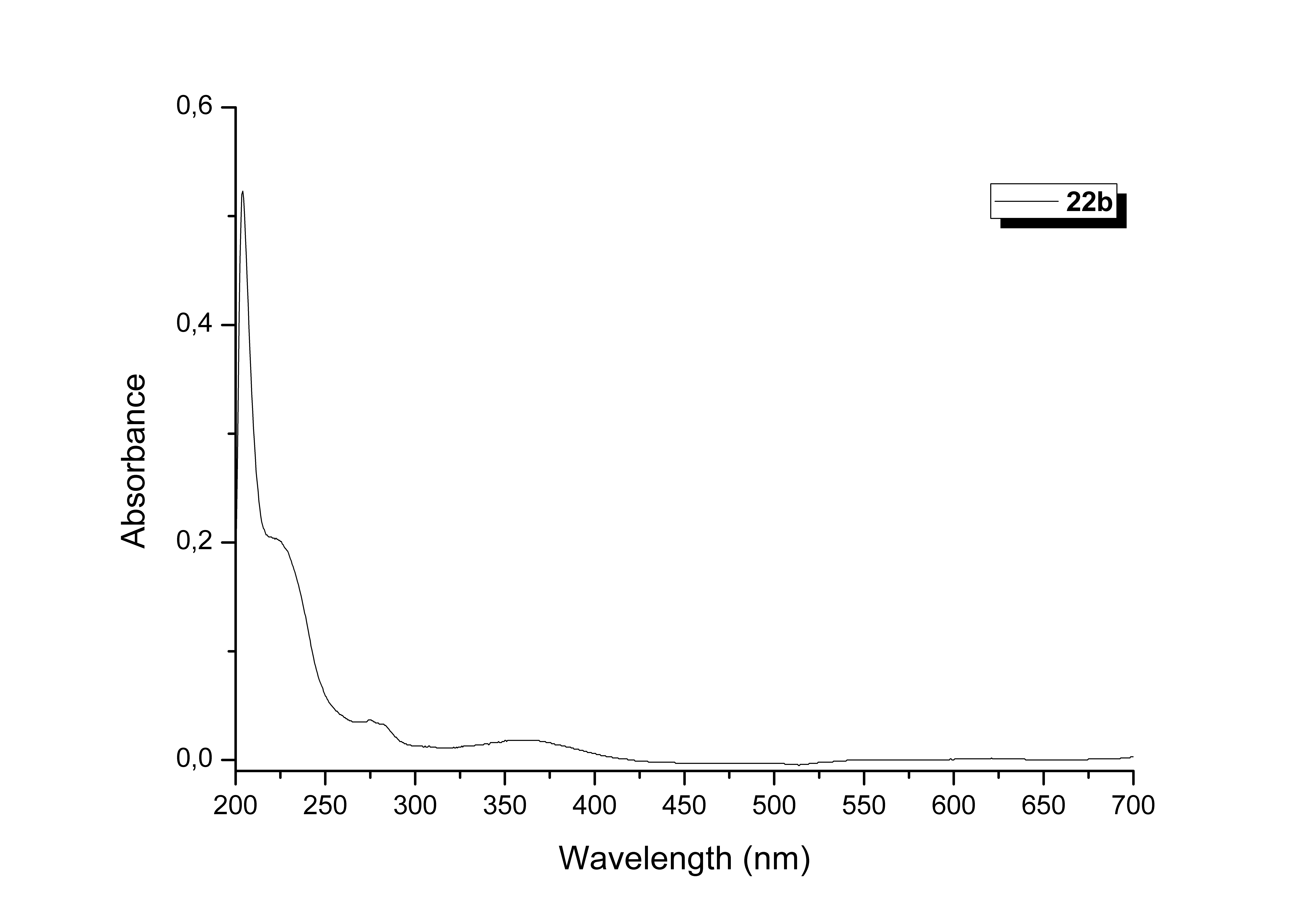
**

**UV-Vis Spectra of 23a**

**
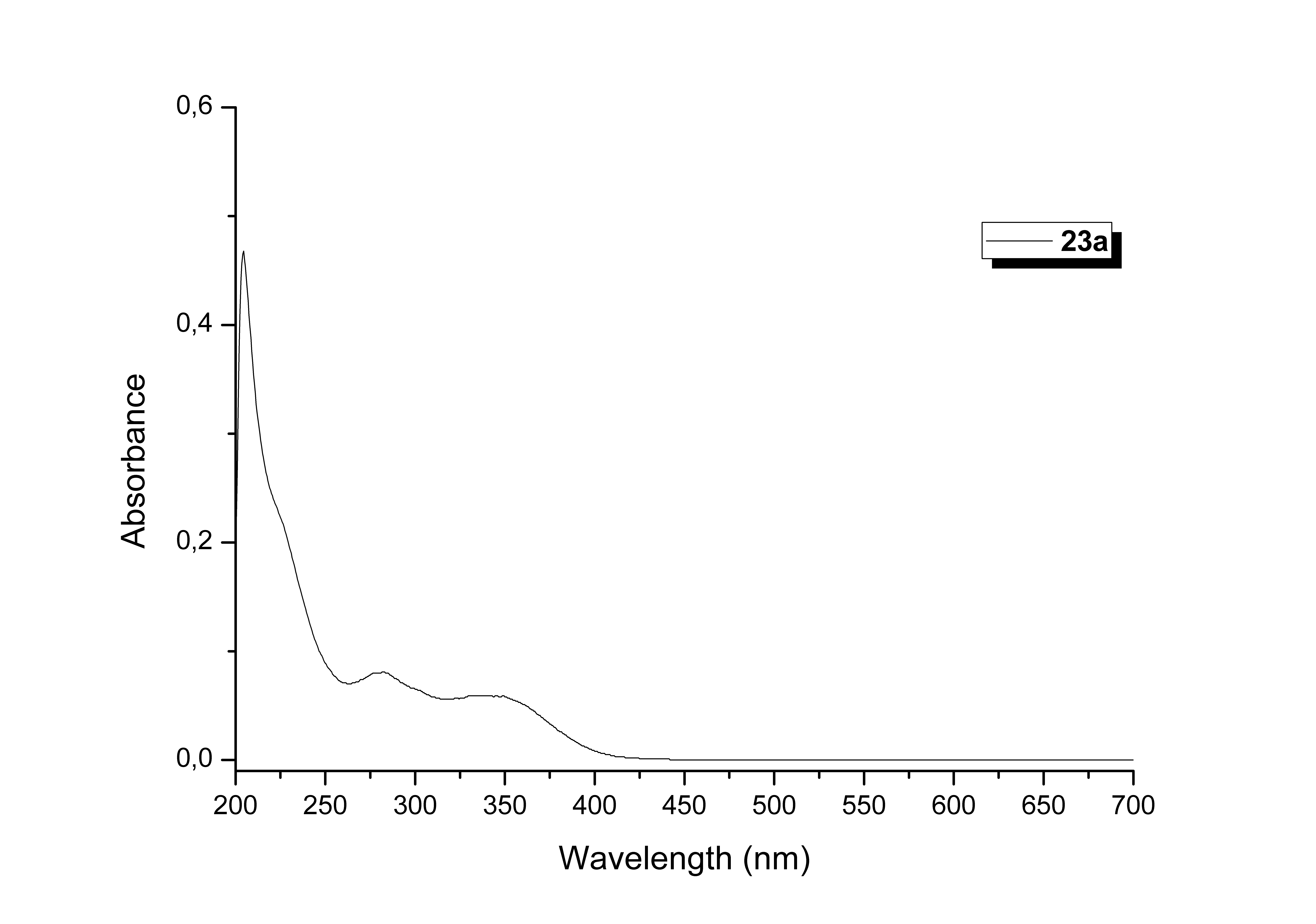
**

**UV-Vis Spectra of 23b**

**
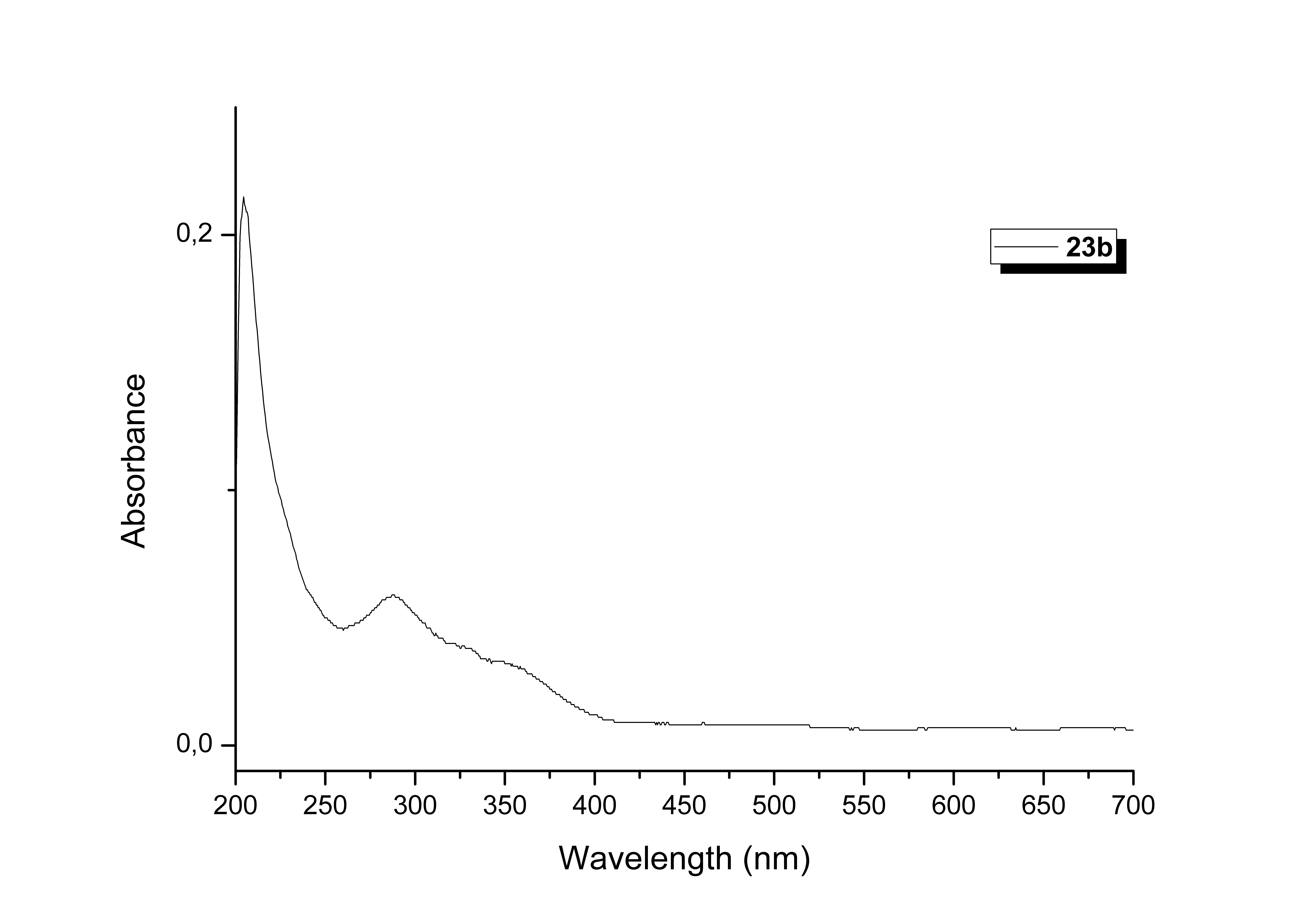
**
